# O–B ← N perturbed polycyclic aromatic hydrocarbons: a straightforward synthesis strategy, and their photophysical and optical waveguide properties

**DOI:** 10.1039/d5sc05407a

**Published:** 2025-09-29

**Authors:** Jinying Zhao, Qihang Yang, Weibin Chen, Nuo Xu, Qing Zhang, Wenhao Zhao, Geng-Geng Luo, Qiuhong Cui, Jianhua Huang

**Affiliations:** a College of Materials Science and Engineering, Huaqiao University Xiamen 361021 China huangjianhua@hqu.edu.cn; b Department of Materials Science and Engineering, School of Physical Sciences and Engineering, Beijing Jiaotong University Beijing 100044 China qhcui@bjtu.edu.cn; c Department of Materials Chemistry, Huzhou University Huzhou 313000 China

## Abstract

Polycyclic aromatic hydrocarbons (PAHs) containing O–B ← N groups in the backbone have demonstrated excellent optical properties but two-dimensionally (2D) extended O–B ← N perturbed PAHs have been rarely reported due to synthetic challenges. Moreover, the applications of these O–B ← N perturbed PAHs have been primarily limited to electroluminescent devices, highlighting the urgent need to explore novel functions of this class of heteroaromatic molecules. This work develops a straightforward synthesis strategy toward O–B ← N perturbed PAHs through 2–3 steps, creating a series of tetra-cycle, octa-cycle, and deca-cycle fused molecules. Photophysical characterization and theoretical simulations of these O–B ← N perturbed PAHs reveal several distinct properties, *e.g.*, high fluorescence quantum yields (70%), aggregation induced emission (AIE) effects, and anti-Kasha emission. Then, through classic solvent diffusion or volatilization methods, these O–B ← N perturbed PAHs further self-assemble into 1D microstructures, *e.g.*, rod or sheet shaped microwires in solutions. These microwires were, for the first time, subjected to optical waveguide measurements, revealing low optical loss coefficients ranging from 10^−2^ to 10^−3^ dB μm^−1^. This work develops an efficient synthesis strategy for 2D extended O–B ← N perturbed PAHs and demonstrates their pioneering applications in optical waveguides, demonstrating the great potential of these O–B ← N perturbed PAHs for micro/nanophotonics.

## Abstract 2

A straightforward strategy with 2–3 steps is developed to synthesize a series of O–B ← N perturbed polycyclic aromatic hydrocarbons, which self-assemble into one-dimensional microwires and exhibit excellent optical waveguide performance.

## Graphical abstract

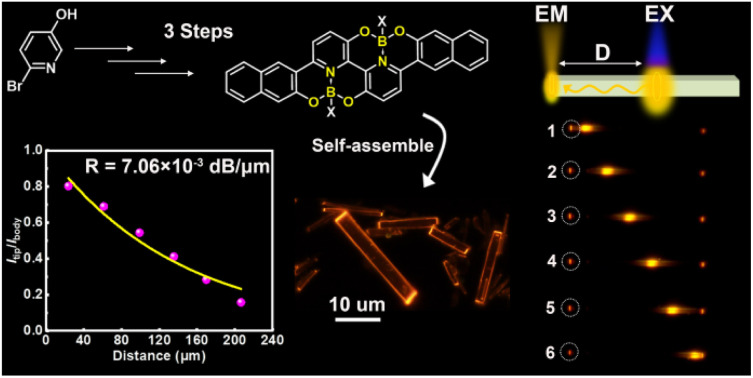

## Introduction

**Fig. 1:**
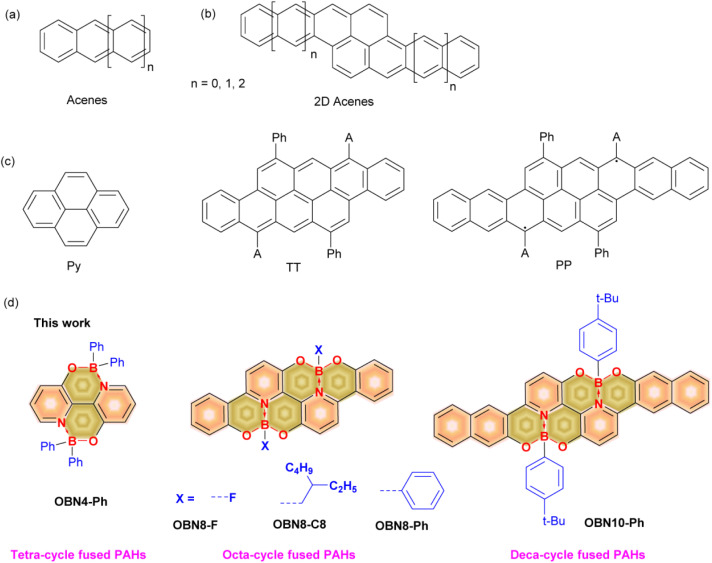
Chemical structures of acenes (a), 2D acenes (b), Py, TT, and PP (c) and O–B ← N perturbed Py, TT, and PP (d).

Acenes, *e.g.*, naphthalene, anthracene, tetracene, and pentacene, are a class of typical polycyclic aromatic hydrocarbons (PAHs) featuring linearly fused benzene rings (Fig. 1a).^1,2^ To regulate their optoelectronic properties and enhance stability, two-dimensionally (2D) extended acenes,^3,4^*e.g.*, two linear acenes fused at the zigzag edge in various manners, have also been developed (Fig. 1b).^5–8^ On the other hand, introducing main group elements, *e.g.*, B, N, O, P, and S, into the skeletons of PAHs has also been demonstrated an effective pathway to tailor their physicochemical properties and enhance stability.^9^ In particular, boron is an ideal element for doping PAHs due to its similar hybridization and strong electron-deficient nature compared to carbon.^10–12^ As such, developing boron-doped PAHs through novel chemistry has been an active area of research in recent years.^13–25^ Moreover, along with O and N, fragments of O–B–O, N–B–N, O–B ← N, and N–B ← N have also been doped into PAHs.^26–40^ Among them, the O–B ← N segment, with a covalent bond between oxygen–boron and a coordination bond between boron–nitrogen, is a useful tool to adjust the energy levels, bandgaps, and photophysical properties of PAHs.^41–43^ On one hand, the electron deficient B ← N fragment and electron-rich O would cooperatively regulate the energy levels and spectra of carbon-based PAHs. On the other hand, introducing polar O–B ← N into carbon-based PAHs would enrich intermolecular interactions.^44^ Additionally, substituents on the tetra-coordinated boron can effectively inhibit strong π–π interactions and enhance solid-state emission.^45^ In previous studies, several O–B ← N embedded conjugated molecules with relatively small conjugation were revealed and applied as emission layers or electron transport layers in organic light emission diodes (OLEDs).^45–50^ Recently, our group reported an O–B ← N embedded unit and applied it to active layers and cathode interlayers of organic photovoltaics (OPVs).^44,51^ Although O–B ← N perturbed backbones with a single boron-center or small conjugation have been feasibly accessed, synthesis of O–B ← N perturbed 2D extended PAHs is still a challenge due to solubility and stability issues originating from the rigid backbones and moisture-sensitive nature of tetracoordinated boron, respectively. Specifically, O–B ← N perturbed 2D extended acenes with two linear acenes fused at the zigzag edge have been rarely reported.

These boron-doped polycyclic heteroaromatic molecules have been applied in various organic electronic devices, for example, OLEDs, OPVs, organic field effect transistors (OFETs), and photodetectors through solution-processing or vacuum evaporation techniques.^34,35,52–54^ Due to backbone rigidity and tailorable weak intermolecular interactions, these polycyclic heteroaromatic molecules can be conveniently assembled into micro- or nano-scaled crystals in appropriate solvents.^55^ In comparison to organic films with amorphous or semi-crystalline morphology, organic crystals, especially single crystals, exhibit fewer unfavorable grain boundaries and lower defect density, which are beneficial to the transport of charge carriers and photons.^56,57^ While numerous studies have been focused on the charge transport performance of molecular crystals based on boron-doped polycyclic heteroaromatic molecules,^36,58^ their photon transport performance, namely, optical waveguide behavior, has not been evaluated. This is particularly true for molecular crystals prepared using boron-doped PAHs, especially O–B ← N perturbed PAHs, although excellent light emission properties have been demonstrated for these molecules.

Herein, we develop a straightforward strategy to synthesize O–B ← N perturbed 2D acenes, *i.e.*, pyrene (Py), tetracenotetracene (TT), and pentacenopentacene (PP). Pyrene is a classic 2D acene whereas TT and PP were reported in 2019 by Frigoli *et al.* (Fig. 1c).^59^ This work reveals a simple and efficient approach toward the synthesis of O–B ← N perturbed Py, TT, and PP for the first time, obtaining a series of tetra-, octa-, and deca-cycle fused heteroaromatic molecules (Fig. 1d). Unique photophysical properties *e.g.*, high photoluminescence quantum yields, aggregation induced emission (AIE), and the anti-Kasha emission phenomenon have been found for these O–B ← N perturbed 2D acenes. Furthermore, 1D microcrystals with rod, sheet, and spindle shapes were prepared using these O–B ← N perturbed 2D acenes in solutions. The optical waveguide properties of the microcrystals were evaluated and low loss coefficients (*R*) of 10^−3^ dB μm^−1^ were observed, indicating that these O–B ← N perturbed PAHs are promising for micro/nanophotonic devices.

## Results and discussion

**Scheme 1:**
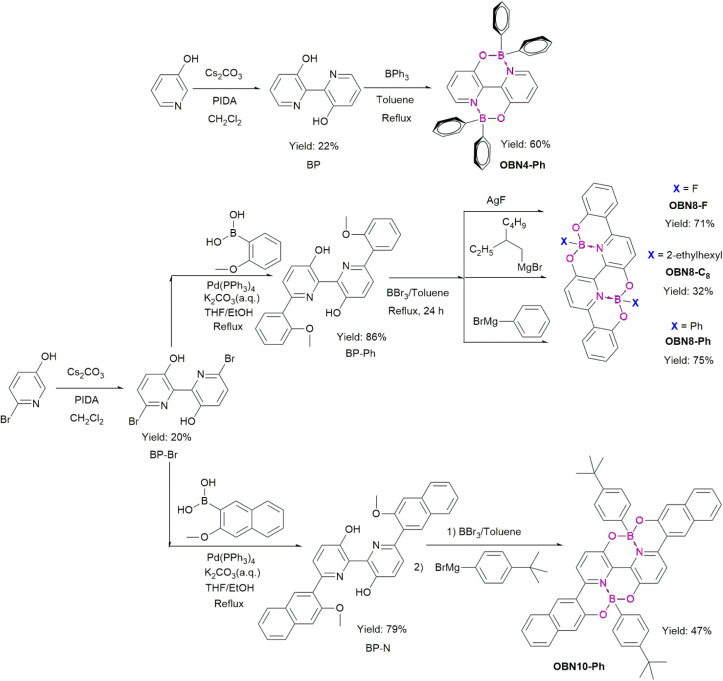
Synthetic routes toward O–B ← N perturbed PAHs.

The synthesis strategy toward these O–B ← N perturbed PAHs is simple and straightforward, generally including 2–3 steps, that is, a metal-free catalyzed C–C coupling reaction, a Pd-catalyzed cross-coupling reaction, and an electrophilic borylation reaction (Fig. S1). As shown in Scheme 1, starting from pyridin-3-ol, the metal-free catalyzed C–C coupling reaction occurs in the presence of Cs_2_CO_3_ and phenyliodine diacetate (PIDA) to obtain [2,2′-bipyridine]-3,3′-diol (BP),^60^ which was further subjected to electrophilic borylation, leading to a phenyl substituted tetra-cycle fused PAH, *i.e.*, OBN4-Ph. To extend the conjugation, 6-bromopyridin-3-ol was selected as the starting material and the same metal-free homocoupling reaction was performed to produce BP–Br. Then, based on BP–Br, Pd-catalyzed crossing-coupling reactions were applied using (2-methoxyphenyl)boronic acid or (3-methoxynaphthalen-2-yl)boronic acid, generating BP–Ph and BP–N, respectively. Finally, cyclization was realized by one-pot electrophilic borylation, leading to three octa-cycle fused PAHs, namely, OBN8-F, OBN8-C8, and OBN8-Ph and a deca-cycle fused PAH, *i.e.*, OBN10-Ph. All the final products exhibit good solubility in common organic solvents except for OBN8-F due to the small volume of the anchored fluorine (F), offering limited solubilization effects. Theoretically, *cis*- and *anti*-isomers are accessible for OBN8-F, OBN8-C8, OBN8-Ph, and OBN10-Ph. However, the ^1^H NMR spectra show only one set of peaks in the aromatic region, indicating that one isomer is preferentially obtained under the current synthesis and post-treatment processes. We calculated the relative electron energy of the *cis* and *anti*-isomers and found that for the phenyl and fluorine substituted OBN8-F, OBN8-Ph, and OBN10-Ph, the *cis*-isomers are more stable whereas for the alkyl substituted OBN8-C8, the *anti*-isomer is more stable (Fig. S11). Thermogravimetric analysis indicates high decomposition temperatures (*T*_d_, 5% weight loss) of 300–450 °C for these molecules (Fig. S7), indicating excellent thermal stability. The stability of these O–B ← N perturbed PAHs was further evaluated by monitoring the UV-Vis absorption spectra in solutions when they are exposed to air. For the phenyl substituted OBN4-Ph, OBN8-Ph, and OBN10-Ph, the spectral curves remain unchanged upon exposure to air for 72–96 h, indicating excellent air stability (Fig. S10). For OBN8-C8, the absorption spectra sharply decline within 10 h and the half-life time was estimated to be 3.4 h (Fig. S8a and S9). However, when the solution was protected by N_2_, OBN8-C8 shows good stability (Fig. S8b). Interestingly, OBN8-F shows gradually enhanced absorption spectra (Fig. S10b), which can be interpreted by the gradually increased concentration due to the slow dissolution of strongly aggregated OBN8-F. These results suggest that most of the O–B ← N perturbed PAHs have excellent thermal and air stability except for the alkyl substituted OBN8-C8.

**Fig. 2:**
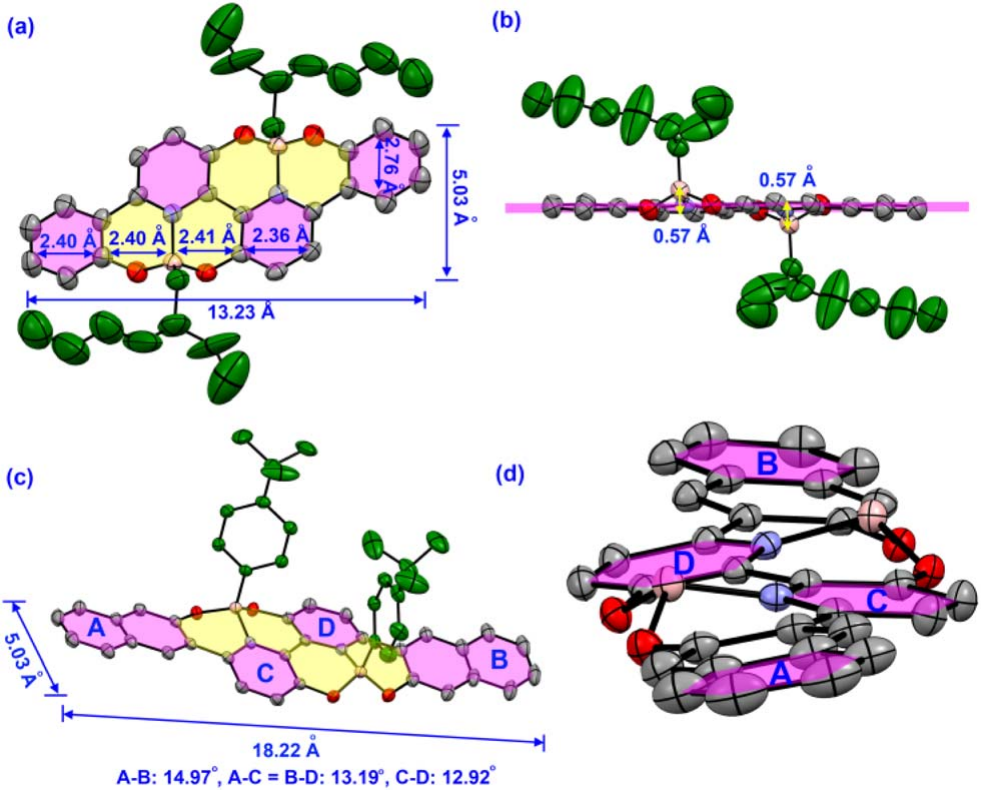
Single crystal structures of OBN8-C8 (a and b) and OBN10-Ph (c and d) determined by X-ray crystallography. The substituents of OBN10-Ph are omitted for clarity in (d).

Single crystals of OBN8-C8 and OBN10-Ph were successfully obtained and X-ray crystallographic tests were performed (Tables S1 and S2). Interestingly, OBN8-C8 exhibits *anti*-configuration, whereas the *cis*-isomer is separated for OBN10-Ph (Fig. 2). Stereochemistry in single crystals is consistent with the relative electronic energies (Fig. S11). For the octa-cycle fused OBN8-C8, the backbone is a 2D extended nanobelt with a length of 13.23 Å and a width of 5.03 Å (Fig. 2a). All the backbone atoms stay in the same plane except for the two borons, which slightly depart from the skeleton by 0.57 Å due to the sp^3^-hybridization of the tetracoordinated boron (Fig. 2b). OBN10-Ph displays an increased length of 18.22 Å and slightly twisted skeleton, with dihedral angles of A–B, A–C, and C–D in the range of 12–15° (Fig. 2c and d). Considering the tetrahedral geometry of boron, the *anti*-configuration would lead to a co-planar backbone, whereas *cis*-substitution on boron would give rise to a twisted backbone. Usually, a twisted backbone can be energetically more favorable than co-planar ones. The steric hindrance of bulky 2-ethylhexyl would trigger *anti*-substitution and an unfavorable co-planar backbone in OBN8-C8 whereas phenyl-substituted OBN10-Ph would favor a *cis*-configuration due to the relatively smaller volume of tetra-butylbenzene. The B ← N bond lengths are *ca*. 1.60 Å for OBN8-C8 (Fig. S2) and OBN10-Ph (Fig. S5), similar to the values revealed in the literature.^37,44^ OBN8-C8 shows slipped face-to-face stacking with a C–C interaction distance of 3.36 Å (Fig. S3). The backbone of OBN8-C8 adopts typical herringbone packing style in the single crystal (Fig. S4). OBN10-Ph exhibits alternate stacking of front and back sides and abundant C–H and C–C interactions can be observed in single crystals (Fig. S6). In general, compact π–π stacking modes are absent in these O–B ← N perturbed 2D PAHs due to the steric hindrance of the side groups, which would partially avoid fluorescence (FL) quenching in solid states.

**Fig. 3:**
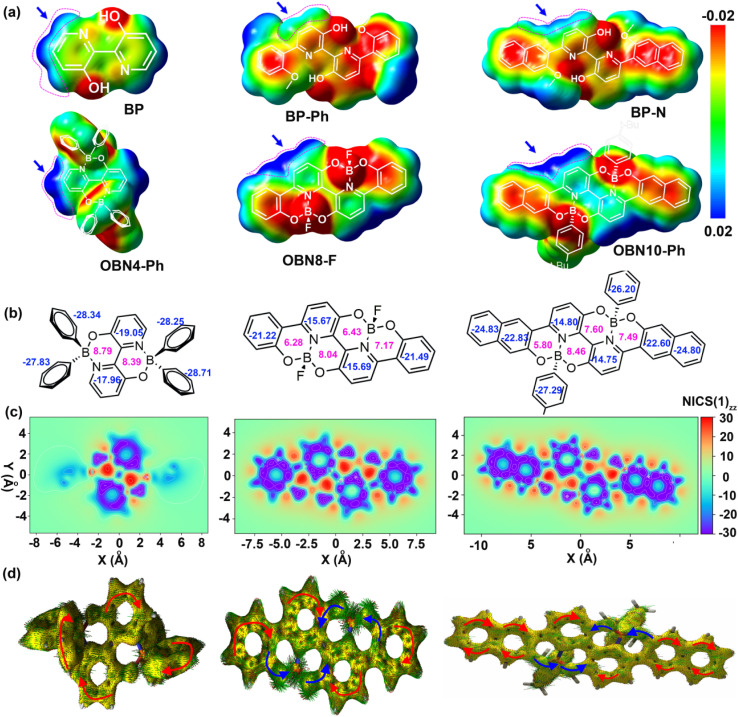
ESP maps of OBN4-Ph, OBN8-F, OBN10-Ph, and three precursors (a) and NICS(1)_*zz*_ plots (b), 2D-ICSS plots (c), and AICD plots (d) of OBN4-Ph, OBN8-F, and OBN10-Ph.

To get insight into the electronic structure and aromaticity, density functional theory (DFT) was adopted to simulate these O–B ← N perturbed PAHs. The electrostatic potential (ESP) maps of the O–B ← N perturbed PAHs and their precursors of BP, BP–P, and BP–N were calculated. As shown in Fig. 3a and S12, the hydroxyl (OH) and pyridine surfaces of the three precursors show strongly negative potential (red color) due to the electron-rich nature of oxygen and nitrogen. After incorporating tetracoordinated boron, the oxygen surface retains a negative potential whereas the potential on the surfaces and edges of pyridine becomes distinctly positive (green to blue), which can be attributed to strong electron donation from nitrogen to the unoccupied orbital of boron. However, for BP–Ph and BP–N, the potential on the bilaterally linked phenyl and naphthalene is not obviously changed after introducing boron into the backbone. These results suggest that the tetracoordinated boron only reduces the surface potential of the pyridine unit but has little effect on the other units of these O–B ← N perturbed PAHs, which is different from the previous B ← N embedded molecules,^61^ where the introduction of boron was found to remarkably change the potential of the whole skeleton from electron-rich to electron-deficient. This can be attributed to the strong electron-donating nature of oxygen, which greatly weakens the electron-accepting ability of boron. The ESP area distribution reveals an extremely positive potential of 25–30 kcal mol^−1^ for the O–B ← N perturbed PAHs (Fig. S13),^62^ corresponding to the electron-deficient edge of pyridine induced by the tetracoordinated boron.

Aromaticity of these O–B ← N perturbed PAHs was evaluated by nuclear independent chemical shifts (NICS), two dimensional iso-chemical shielding surface (2D-ICSS) maps, and anisotropy of the induced current density (AICD) analysis.^63,64^ As shown in Fig. 3b and S14, the two pyridine cycles exhibit apparent aromaticity with minus NICS(1)_*zz*_ and the NICS(1)_*zz*_ values of O–B ← N embedded cycles are in the range of +5 to +8 ppm, suggesting anti-aromaticity. As the conjugation is extended, the NICS(1)_*zz*_ values gradually decrease from the inner phenyl (*ca*. −22 ppm) to the marginal phenyl (*ca*. −24 ppm), demonstrating enhanced aromaticity. Interestingly, the phenyl substituted on the boron exhibits the strongest aromaticity with NICS(1)_*zz*_ values ranging from −26 to −28 ppm, elucidating that the aromaticity of the linked phenyl is negligibly impacted by the boron. These results illustrate that embedding O–B ← N into the backbone would readily destroy the aromaticity but the aromaticity of side phenyl linked to boron is not suppressed. The 2D-ICSS plots solidly support the NICS results with strong aromaticity for the pyridine and other phenyls (blue) but anti-aromaticity for the O–B ← N embedded cycles (red) (Fig. 3c). The AICD plots of OBN4-Ph show clockwise ring current for the pyridine and the phenyls linked on the boron, supporting the aromaticity (Fig. 3d). For OBN8-F and OBN10-Ph, a clockwise ring current appears on the pyridine and other phenyls but a counterclockwise ring current can be observed over the O–B ← N embedded cycles, consistent with the NICS and 2D-ICSS results.

**Fig. 4:**
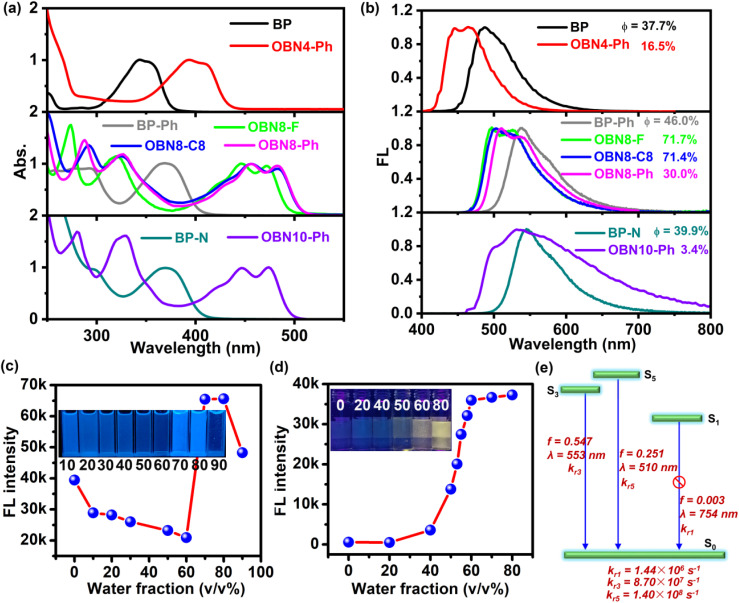
UV-Vis absorption (a) and FL (b) spectra of the three precursors and the corresponding O–B ← N perturbed PAHs in diluted chloroform solutions (10^−5^ M). The variation of FL intensity of OBN4-Ph (c) and OBN10-Ph (d) in THF/H_2_O solvent with different water fractions. The insets are the solution photos under ultraviolet light radiation (365 nm). The schematic illustration of primary transitions for OBN10-Ph from excited states to the ground state (S_0_) simulated by b3lyp/6-31g (d, p) (e), *k*_r_ represents radiative rates.

Photophysical properties of the five O–B ← N perturbed PAHs and the three precursors were evaluated by recording the UV-Vis absorption and FL spectra in diluted chloroform. As shown in Fig. 4a, in contrast to the precursors, the O–B ← N perturbed PHAs show red-shifted absorption due to the introduction of electron-deficient boron. For octa-cycle fused PAHs (OBN8-F, OBN8-C8, and OBN8-Ph) and deca-cycle fused PAHs (OBN10-Ph), two strong absorption bands appear in the range of 250–380 nm, verifying that multiple transitions are allowed in the ultraviolet region, which may be ascribed to the π–π* or *n*–π* transitions. The lower energy absorption bands at 400–500 nm can be attributed to the intramolecular charge transfer (ICT) transitions. The fluorine substituted OBN8-F exhibits slightly blue-shifted ICT peaks in comparison to those of alkyl- and phenyl-substituted OBN8-C8 and OBN8-Ph, which can be explained by the additional ICT effects from the electron-rich alkyl and phenyl groups donating to the electron-deficient boron.^50^ From OBN4-Ph to OBN8-Ph, the bathochromic absorption can be related to the extended conjugation. However, from octa-cycle fused OBN8-Ph to deca-cycle fused OBN10-Ph, the ICT absorption is mildly blue-shifted, which can be related to the twisted backbone of OBN10-Ph, as clarified in the single crystal data. The optical bandgaps calculated from the films absorption (Fig. S25) are 2.78 eV for OBN4-Ph and *ca*. 2.40 eV for OBN8-C8, OBN8-Ph, and OBN10-Ph (Table 1).

**Table 1:** Photophysical properties of the three precursors and five O–B ← N perturbed PAHs in chloroform solutions and in films

	Absorption				Emission					
	*λ* ^s^_m_ (nm)^a^	*λ* ^f^_m_ (nm)^b^	*λ* ^f^_edge_ (nm)^c^	*E* ^opt^_g_ (eV)^d^	*λ*_ex_ (nm)^e^	*λ* ^FL^_m_ (nm)^f^	*Φ* (%)^g^	Δ*ν* (nm)^h^	*τ* (ns)^i^	*k*_nr_ (10^8^ s^−1^)^j^
BP	343	354	420	2.95	345	488	37.69	145	3.44	1.81
OBN4-Ph	394/410	401/423	446	2.78	390	465	16.58	71	2.42	3.45
BP–Ph	367	373	433	2.86	373	538	46.06	171	2.71	1.99
OBN8-F	447/472	—	—	—	469	498	71.67	51	5.91	0.48
OBN8-C8	456/482	464/494	521	2.38	469	502	71.38	46	5.72	0.50
OBN8-Ph	456/483	463/492	520	2.38	469	510	29.94	54	2.95	2.37
BP–N	370	373	435	2.85	369	545	39.92	175	2.00	3.00
OBN10-Ph	447/474	455/484	519	2.39	469	531	3.38	84	0.69	14.00

a Absorption maximum in solutions.

b Absorption maximum in films.

c Absorption edges in films.

d Optical bandgaps calculated by 1240/*λ*^f^_edge_.

e Excitation wavelength.

f Emission peaks in solutions.

g Absolute fluorescence quantum yields measured using an integrating sphere.

h Stokes shifts calculated by *λ*^FL^_m_ − *λ*^s^_m_.

i FL life-times.

j Non-radiative decay rates calculated by (1 − *Φ*)/*τ*.

As shown in the FL spectra (Fig. 4b), the emission peaks exhibit significant hypsochromic shifts from the precursors to the O–B ← N perturbed PAHs. Therefore, sharply different Stokes shifts (Δ*ν*) between the precursors and the corresponding O–B ← N perturbed PAHs can be deduced. As summarized in Table 1 and plotted in Fig. S26, the Δ*ν* values suddenly decrease from 140–170 nm for precursors to 50–80 nm for O–B ← N perturbed PAHs. In contrast to the precursors of BP, BP–Ph, and BP–N with rotatable bonds, the reduced Stokes shifts of O–B ← N perturbed PAHs can be interpreted by the rigid backbones locked by the boron.

Fluorescence quantum yields (*Φ*) in solutions were measured using an integrating sphere. Interestingly, the variation of *Φ* values from precursors to O–B ← N perturbed PAHs is closely related to the backbones and substituents (Fig. S27). From BP–Ph to fluorine-substituted OBN8-F and alkyl-substituted OBN8-C8, the *Φ* values remarkably increased from 46.0% to 71.7% and 71.4% due to the increased backbone rigidity, leading to decreased non-radiative decay. Otherwise, when the substituents on the boron are phenyl, the O–B ← N perturbed PAHs display decreased *Φ* values in comparison to their precursors. For example, from BP to OBN4-Ph, BP–Ph to OBN8-Ph, and BP–N to OBN10-Ph, the *Φ* values decreased from 37.7% to 16.5%, 46% to 30.0%, and 39.9% to 3.4%, respectively. The FL life times (*τ*) were measured (Fig. S28) and the rates of non-radiative decay (*k*_nr_) were calculated by (1 − *Φ*)/*τ*. From the precursors to the phenyl substituted backbones, the *k*_nr_ values apparently increase (Fig. S29). Therefore, the decreased *Φ* values in solutions upon anchoring phenyl to the backbone are attributable to the freely rotatable phenyl,^65^ resulting in enhanced non-radiative decay.

We also measured the FL spectra and *Φ* values of these O–B ← N perturbed PAHs dispersed in PMMA films (1% w/w) (Fig. S30) as well as in their crystal forms (Fig. S31 and Table S11). Interestingly, the *Φ* values significantly increase from solutions to PMMA blends for OBN4-Ph (16.5% *vs.* 45.5%) and slightly for OBN10-Ph (3.4% *vs.* 4.2%), which can be explained by the inhibited rotation of phenyls in PMMA films, giving rise to promoted radiative decay. Moreover, the *Φ* values in crystals are also greater than those in solutions for OBN4-Ph (45.0% *vs.* 16.5%) and OBN10-Ph (8.3% *vs.* 3.4%). These results demonstrate that OBN4-Ph and OBN10-Ph presumably have aggregation induced emission (AIE) effects. This drives us to further confirm the AIE properties of OBN4-Ph and OBN10-Ph by monitoring the FL intensity in THF/H_2_O mix solvents with different water fractions (*f*_water_). As shown in Fig. S32a and 4c, the emission intensity of OBN4-Ph gradually decreases when the *f*_water_ increases from 0% to 60% but suddenly increases by 3 fold when *f*_water_ increases to 70%. For OBN10-Ph, the emission intensity monotonically increases from *f*_water_ = 0% to 80%, exhibiting typical AIE effects (Fig. S32b and 4d). Photographs of the solutions under ultraviolet light radiation (365 nm) also support the AIE properties of OBN4-Ph and OBN10-Ph (inset in Fig. 4c and d).

Time-dependent density functional theory (TD-DFT) calculations were performed to predict the transitions from the ground state to the excited states (Fig. S15 and Tables S3–S10). In general, the predicted transitions and oscillator strengths (*f*) agree well with the experimentally measured spectra except for some deviations observed in the higher energy transitions. We analyzed frontier orbital transitions from S_0_ to S_1_, S_2_, S_3_, S_4_, and S_5_ and schematically illustrated these processes, as shown in Fig. S16–S23. Interestingly, all the molecules exhibit allowed S_0_ → S_1_ transitions with high *f* values of *ca.* 0.05–0.60 except for OBN10-Ph, whose *f* value of S_0_ → S_1_ is 0.0 (Fig. S23), suggesting a forbidden transition from the ground to the first excited state. This result hints that the radiative process of OBN10-Ph from S_1_ to S_0_ also may be forbidden, following the so-called anti-Kasha emission feature.^66^ To further verify our guess, the excited states of OBN10-Ph were simulated. As expected, the *f* value of S_1_ → S_0_ (*λ* = 754 nm) is extremely low (0.003) whereas S_3_ → S_0_ (*λ* = 553 nm) and S_5_ → S_0_ (*λ* = 510 nm) show high *f* values of 0.547 and 0.251 (Fig. 4e), respectively. More importantly, the radiative rate of *k*_r1_ (1.44 × 10^6^ s^−1^) is significantly smaller than those of *k*_r3_ (8.70 × 10^7^ s^−1^) and *k*_r5_ (1.40 × 10^8^ s^−1^) by at least one order of magnitude. These theoretical calculations preliminarily prove the anti-Kasha radiation of OBN10-Ph. To further confirm this inference, the excitation-wavelength-dependent emission spectra of OBN10-Ph in chloroform solution were measured, which is a powerful experiment to affirm the anti-Kasha emission.^66^ As the excitation wavelength varies from 330 to 450 nm, remarkable changes of emission spectra are observed (Fig. S33a) and the excitation-wavelength-independent peaks at 533 and 523 nm can be ascribed to transitions of S_3_ → S_0_ and S_5_ → S_0_, solidly supporting the anti-Kasha emission. For comparison, OBN8-Ph was also subjected to the same measurements (Fig. S33b), showing emission spectra independent of the excitation wavelength, consistent with regular radiation following the classic Kasha rule. High internal conversion rates (*k*_ic_) of up to 10^11^ s^−1^ were calculated between S3/S5 and S1 for OBN10-Ph (Fig. S24), much faster than the FL radiation rates (*ca*. 10^9^ s^−1^). This is different from the traditional anti-Kasha mechanism, which requires a large energy gap and small *k*_ic_ between S1 and adjacent excited states.^67,68^ Temperature-dependent FL spectra of OBN10-Ph show enhanced intensity from 80 K to 130 K (Fig. S34), evidently supporting the thermal activation processes from S_1_ to higher electronic states.^15,26^ Further elevating the temperature to 280 K gives rise to decreased FL intensity, which could be ascribed to the enhanced non-radiative decay at high temperatures. For comparison, OBN8-Ph was also subjected to temperature-resolved spectra tests and monotonically decreased intensity was observed as the temperature increased from 80 K to 300 K (Fig. S35), excluding thermal activation processes. These results evidently show that the anti-Kasha emission of OBN10-Ph can be attributed to the prohibited emission from S_1_, the fast internal conversion rates from S_3_/S_5_ to S_1_, and thermal repopulation processes from S_1_ to higher electronic states, *e.g.*, S_3_ and S_5_, all of which lead to the strong emission from S_3_ and S_5_.

The phosphorescence spectra of the five O–B ← N perturbed PAHs in PMMA films (1.0 wt%) were recorded under 77 K with lifetimes (*τ*_p_) in the range of 1–33 ms (Fig. S36 and S37). Cyclic voltammetry (CV) curves of these O–B ← N perturbed PAHs were measured in chloroform solutions. Electrochemical properties of OBN8-F could not be measured due to its poor solubility in solvents. As shown in Fig. S38, OBN4-Ph and OBN8-C8 exhibit reversible reduction peaks whereas OBN8-Ph displays quasi-reversible oxidation peaks. OBN10-Ph gives irreversible redox peaks due to its limited solubility. As the conjugation extends from OBN4-Ph to OBN8-Ph and OBN10-Ph, the reduction potentials (−1.26 *vs.* −0.78 *vs.* −0.60 V) gradually increase, indicating enhanced electron affinity.

Considering the excellent photoluminescence (PL) properties and extended skeleton conjugation, we selected OBN8-C8, OBN8-Ph, and OBN10-Ph to prepare microstructures using solvent diffusion or volatilization methods (Fig. S39–S41). Interestingly, well-defined microcrystals can be obtained with spindle, rod, and sheet shapes, respectively, for OBN8-C8, OBN8-Ph, and OBN10-Ph (Fig. S42–S44).

**Fig. 5:**
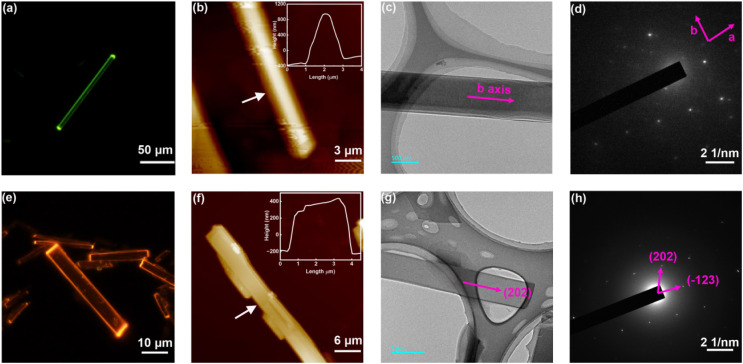
FL microscopy images (a and e), AFM images (b and f), TEM images (c and g), and SAED patterns (d and h) of OBN8-Ph and OBN10-Ph microcrystals.

For OBN8-Ph and OBN10-Ph, upon ultraviolet excitation, the two end points of 1D microcrystals exhibit bright luminescence spots whereas the bodies are almost non-luminescent (Fig. 5a and e). It's obvious that the 1D microcrystals of OBN8-Ph and OBN10-Ph are capable of absorbing the excitation light and propagating the light wave to the end points, which is the typical feature of optical waveguides. Atomic force microscopy (AFM) images reveal a thickness of *ca*. 1.2 μm for OBN8-Ph microcrystals, confirming the rod shape (Fig. 5b). OBN10-Ph microcrystals show a small thickness of *ca*. 0.5 μm, supporting the sheet shape (Fig. 5f). Transmission electron microscopy (TEM) and selected area electron diffraction (SAED) were adopted to further examine the micromorphology and crystalline properties of the microwires. Consistent with the AFM images, the TEM images of both OBN8-Ph and OBN10-Ph microwires present smooth surfaces, which are beneficial for light wave propagation (Fig. 5c and g). The bright and uniformly distributed diffraction dots observed in the SAED patterns of OBN8-Ph and OBN10-Ph microwires demonstrate single crystalline nature throughout the whole microstructure (Fig. 5d and h). According to the single crystal XRD data, the microwires of OBN10-Ph were grown along the (202) direction. The well-defined 1D microstructures and excellent crystalline properties of these microwires are advantageous to transport exciton polaritons (EPs),^69^ a kind of mixed light-matter quasi-particles formed by the coupling of excitons and photons, which is a critical characteristic for optical waveguides.

**Fig. 6:**
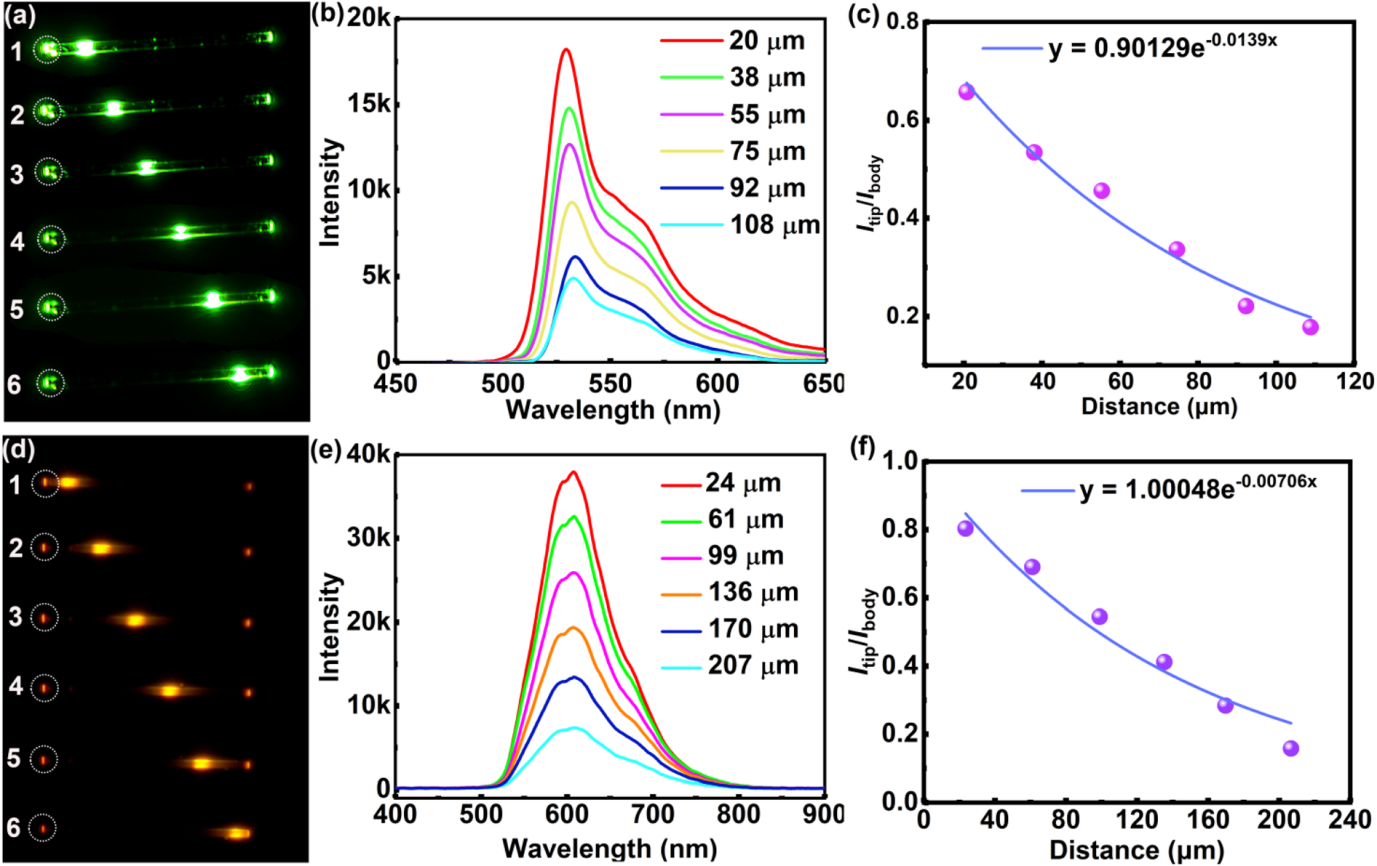
Microarea PL images of OBN8-Ph and OBN10-Ph microwires obtained by exciting the same microrod at six different positions (a and d); PL spectra collected at the left tips of OBN8-Ph and OBN10-Ph microwires by exciting at different positions (b and e); plots of *I*_tip_/*I*_body_*versus* propagating distance (*D*) and the fitted curves of OBN8-Ph and OBN10-Ph microwires (c and f).

The optical waveguide performance of the OBN8-Ph and OBN10-Ph microwires was evaluated by locally exciting the microwires at different positions and recording the spatially resolved PL spectra as a function of propagation distance. As shown in Fig. 6a and d, the brightness at the left tips (points 1 to 6) of both OBN8-Ph and OBN10-Ph microwires gradually decreases as the excitation positions spatially depart from the left ends. On the other hand, the emission intensities at the right tips gradually increase as the excitation points shift from left to right. Distance-dependent PL spectra were collected from points 1 to 6 by increasing the propagation distance of the source light (Fig. 6b and e). Typical light waveguide behavior is observed for OBN8-Ph and OBN10-Ph microwires with decreased intensities of PL spectra as the excitation points move from left to right. The decay of PL intensity with respect to the propagation distances (*D*) can be quantitatively determined by the ratio of PL intensity at the emitting ends (*I*_tip_) to the excitation intensity (*I*_body_). By plotting *I*_tip_/*I*_body_*versus D* and single-exponentially fitting the plots using *I*_tip_/*I*_body_ = *A* exp(−*RD*), the optical loss coefficient (*R*) can be deduced. As shown in Fig. 6c and f, low *R* values of 1.39 × 10^−2^ and 7.06 × 10^−3^ dB μm^−1^ are estimated for OBN8-Ph and OBN10-Ph microwires, respectively, which are comparable or better than those of the 1D microcrystals prepared from luminescent organic conjugated molecules based on perylene diimide, perylene, fluorene, *etc.* (Table S12).^70–75^ In general, the impressive optical waveguide performance of OBN8-Ph and OBN10-Ph can be related to their excellent PL properties and 2D extended conjugation, enabling spontaneous self-assembly into long range ordered 1D microwires. In contrast to OBN8-Ph, the lower optical loss of OBN10-Ph can be readily ascribed to the extended conjugation and distinct AIE properties. These results suggest that O–B ← N perturbed PAHs are promising candidates to prepare microstructures with excellent optical waveguide performance, which are crucial components for micro/nanophotonic devices.

## Conclusions

A series of O–B ← N perturbed polycyclic aromatic hydrocarbons (PAHs) with tetra-cycle (OBN4-Ph), octa-cycle (OBN8-F, OBN8-C8, and OBN8-Ph), and deca-cycle (OBN10-Ph) fused backbones were synthesized using a straightforward strategy through 2–3 steps, and were structurally identified by ^1^H, ^13^C, ^11^B NMR, and MALDI-TOF MS. X-ray crystallography revealed excellent skeleton co-planarity for octa-cycle fused OBN8-C8 and a slightly twisted backbone for deca-cycle fused OBN10-Ph. The tetrahedral configuration of boron inhibits compact π–π stacking due to the steric hindrance of side groups linked to boron. Theoretical simulations showed the anti-aromatic nature of the O–B ← N embedded cycles. Distinct photophysical properties, *e.g.*, high fluorescence quantum yields, aggregation induced emission (AIE) effects, anti-Kasha emission were demonstrated among these O–B ← N perturbed PAHs. 1D microwires with rod and sheet shapes were prepared respectively for OBN8-Ph and OBN10-Ph using solvent diffusion or volatilization methods, and were characterized by FL microscopy, AFM, TEM, and SAED. Optical waveguide behavior of the OBN8-Ph and OBN10-Ph microwires was estimated by exciting the microwires at different sites in the body and collecting the photoluminescence spectra at the tips. Low optical loss coefficients of *ca.* 10^−2^ and 10^−3^ dB μm^−1^ were observed for OBN8-Ph and OBN10-Ph microwires, respectively, indicating the great potential of these O–B ← N perturbed PAHs for micro/nanophotonics.

## Author contributions

J. Z.: investigation, data curation, and formal analysis. Q. Y.: investigation, data curation, and formal analysis. W. C.: software and validation. N. X.: investigation. Q. Z.: software. W. Z.: investigation. G. L.: methodology. Q. C.: supervision and funding acquisition. J. H.: writing – conceptualization, original draft, review & editing, funding acquisition.

## Conflicts of interest

There are no conflicts to declare.

## Supplementary Material

SC-016-D5SC05407A-s001

SC-016-D5SC05407A-s002

## Data Availability

CCDC 2472866 and 2472869 (OBN8-C8 and OBN10-Ph) contain the supplementary crystallographic data for this paper.^76*a*,*b*^ The data supporting this article have been included as part of the supplementary information (SI). Supplementary information: including materials, experimental methods, molecular synthesis, X-ray crystallography, stability measurements, detailed theoretical simulations, preparation and optical waveguide tests of microcrystals, NMR, and HRMS spectra is available. See DOI: https://doi.org/10.1039/d5sc05407a.
