# Supplementary material for: O–B ← N perturbed polycyclic aromatic hydrocarbons: a straightforward synthesis strategy, and their photophysical and optical waveguide properties
Source: Chem Sci. 2025 Sep 29;16(43):20445–56. doi: 10.1039/d5sc05407a (PMC12498248; doi:10.1039/d5sc05407a)
Supplement: SC-016-D5SC05407A-s001 [file SC-016-D5SC05407A-s001.pdf]

Supplementary Information (SI) for Chemical Science.
This journal is © The Royal Society of Chemistry 2025

Supplementary Information for

O-B←N Perturbed Polycyclic Aromatic Hydrocarbons: A Straightforward Synthesis Strategy, Photophysical, and Optical Waveguide Properties

Jinying Zhao,[a] Qihang Yang, [b] Weibin Chen,[a] Nuo Xu,[a] Qing Zhang,[c] Wenhao Zhao,[b] Geng-Geng Luo,[a] Qiuhong Cui,[b] and Jianhua Huang*[a]*

[a] College of Materials Science and Engineering, Huaqiao University, Xiamen 361021, China

[b] Department of Materials Science and Engineering, School of Physical Sciences and Engineering, Beijing Jiaotong University, Beijing 100044, China

[c] Department of Materials Chemistry, Huzhou University, Huzhou 313000, China

*E-mail: qhcui@bjtu.edu.cn; huangjianhua@hqu.edu.cn

Contents

1. Materials, instruments, and measurements 1
2. Materials synthesis 2
3. X-ray crystallography 8
4. Stability measurements 12
5. Theoretical simulations. 14
6. Photophysical properties 24
7. Synthesis, characterization, and optical waveguide tests of crystals 32
8. NMR spectra and HRMS 35
9. References 51

1. Materials, instruments, and measurements

All commercially available chemicals were used without further purification unless otherwise noted. Tetrahydrofuran and toluene were distilled freshly from sodium benzophenone ketyl under nitrogen before use. Anhydrous ethyldiisopropylamine and anhydrous Dichloromethane were purchased from commercial sources and used as received. Unless otherwise stated, all reactions were carried out under N_2 atmosphere using standard Schlenk-line techniques. Column chromatography was performed with silica gel (200-300 mesh). Analytical thin-layer chromatography (TLC) was performed on 0.2 mm silica gel-coated glass sheets with F254 indicator. All yields given referred to isolated yields. Nuclear Magnetic Resonance (NMR) spectra were recorded on Bruker Avance III 500 MHz NMR spectrometers at 298 K. Chemical shifts were reported in ppm. Coupling constants (*J* values) were reported in Hertz. ^{1}H NMR chemical shifts were referenced to TMS (0 ppm), $CDCl_3$ (7.26 ppm). ^{13}C NMR chemical shifts were referenced to $CDCl_3$ (77.00 ppm). The ^{11}B NMR spectra samples were tested in the quartz NMR tube with boron content lower than 0.1 ppm. Mass spectra were performed on a Bruker Daltonics Flex matrix-assisted laser desorption ionization time of flight mass spectrometer (MALDI-TOF-MS). Thermogravimetric analysis (TGA) was recorded on DTG-50H at a heating rate of 10 ºC/min under N_2 condition. The decomposition temperature (T_d) corresponded to 5 % loss of weight.

Absorption and emission spectra of solution were recorded in a conventional quartz cell (10 mm light path) on a standard, commercial spectrometer. The solution concentration in conventional quartz cell was 1.0 $\times 10^{-5}$ M. The film samples were fabricated by spin-coating the CF solutions onto quartz substrates. Crystal samples were prepared by re-crystallization from DCM/MeOH mixed solvent and transferred from the solution to quartz substrates. The concentration of pure film spin coating solution is 1 mg/mL in chloroform. PMMA distributed samples were spin-coated from the blend solution of O-B←N perturbed PAHs and PMMA (10 mg PMMA and 0.1 mg O-B←N molecules dissolved in 1 mL chloroform). Absorption spectra were recorded on a PerkinElmer Lambda 1050 Spectrometer. Photoluminescence spectra were recorded on an Edinburgh FLS1000 spectrometer. Fluorescence decays were measured, employing EPL as light source, by time-correlated single-photon counting (TCSPC) technique then fitted using exponential convolution function to determine the lifetimes of prompt fluorescence. If the lifetimes of prompt fluorescence are less than 10 ns, the liquid sample used the aqueous solution of silicon dioxide, and the solid sample used the sample itself to carry out IRF (instrument response function) to eliminate the inaccurate of the instrument itself. Using microsecond lamp as light source, the phosphorescence decay was measured in multi-channel scaling (MCS) technique, and the phosphorescence lifetime was determined by fitting with

exponential convolution function. Absolute photoluminescent quantum yield was determined using an integrating sphere with Xe lamp as light source. The Oxford accessories were used for variable temperature fluorescence spectrum and phosphorescence test, and the coolant was liquid nitrogen. The phosphorescence spectrum was determined by microsecond lamp.

Cyclic voltammetry (CV) was carried out in 0.1 M n-Bu_4NPF_6 deoxygenated solution in dichloromethane using a three-electrode configuration (glassy carbon as working electrode, Pt as counter electrode, and saturated calomel electrode as pseudo-reference) and a CHI660e electrochemical workstation. The solutions were bubbled with argon for 5 min prior to the test. The ferrocene/ferrocenium (Fc/Fc^+) couple was served as external reference. The materials were dissolved in the electrolyte at a concentration of 1 mg/mL for measurements. The scanning rate was 100 mV/s. The working electrode was polished with a 0.05 μm alumina paste and washed with water, ethanol and dichloromethane before use.

2. Materials synthesis

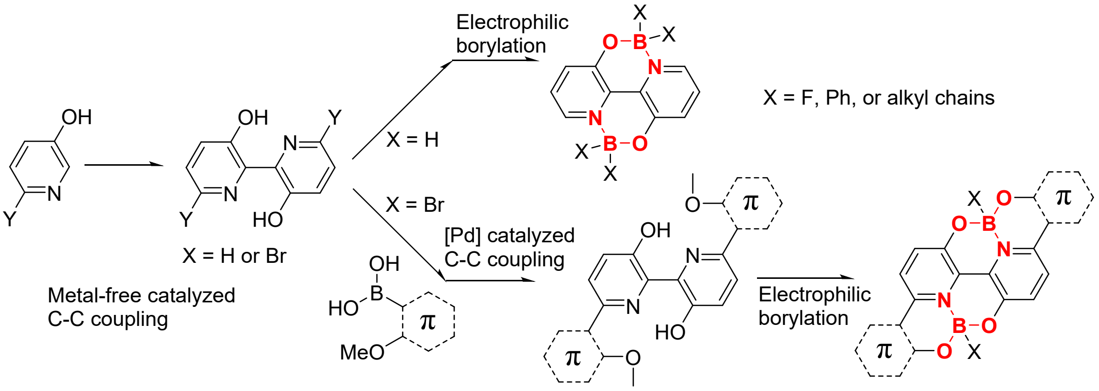

Figure S1. Synthesis strategy toward the O-B←N perturbed PAHs.

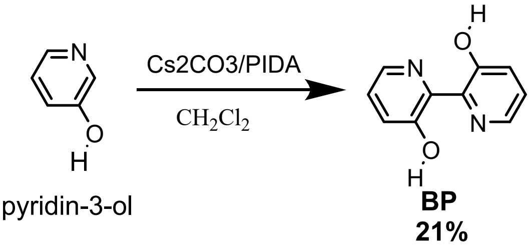

Synthesis of BP

To a stirred solution of the pyridin-3-ol (500 mg, 5.26 mmol) in CH_2Cl_2 (8 mL) were added Cs_2CO_3 (286 mg, 0.88 mmol) and PIDA (565 mg, 1.75 mmol). The mixture was stirred at room temperature for 2 h and then concentrated in vacuo. Purification of the residue was performed by column chromatography (petroleum ether/ ethyl acetate = 10:1). The resultant was recrystallized from CH_2Cl_2/hexane (1:4)

to obtain pale yellow crystals (106 mg, 21% yield). ^{1}H NMR (500 MHz, $CDCl_3$, δ, ppm): 14.74 (br, 1H), 8.09 (d, J = 5.0 Hz, 1H), 7.49 (d, J = 10 Hz, 1H), 7.32 (dd, J_1 = 10, J_2 = 5 Hz, 1H).

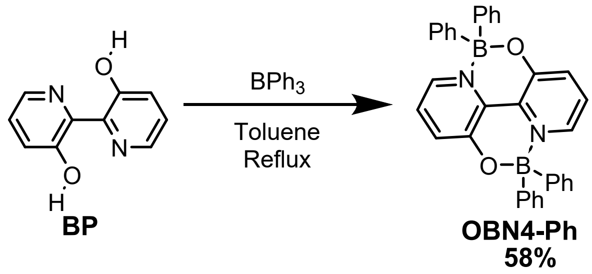

Synthesis of OBN4-Ph

To a stirred solution of BP (50 mg, 0.27 mmol) in toluene (10 mL), BPh_3 (167 mg, 0.69 mmol) was added dropwise. Next, the solution was sealed and stirred at 110 °C or further 12 h. The mixture was cooled to room temperature and the solvent was removed. Then, the product was extracted with CH_2Cl_2 and saturated ammonium chloride solution. The organic layer was dried over anhydrous $MgSO_4$, filtrated and evaporated using a rotary evaporator. The solid was purified by silica gel column chromatography (petroleum ether/dichloromethane = 5: 1). The resultant was further recrystallized from CH_2Cl_2/hexane (1:2) to obtain white crystals (92 mg, 58% yield). ^{1}H NMR (500 MHz, $CDCl_3$, δ, ppm): 7.76 (d, J = 10 Hz, 2H), 7.52 (d, J = 5.0 Hz, 2H), 7.40 (dd, J = 10 Hz, J_2 = 5 Hz, 2H), 7.23–7.00 (m, 20H). ^{13}C NMR (500 MHz, $CDCl_3$, δ, ppm): 156.01, 135.22, 133.46, 133.12, 132.64, 132.26, 127.52, 127.22, 126.87. ^{11}B NMR (500 MHz, $CDCl_3$) δ 6.83 ppm. MALDI-TOF MS *m/z*: $[M + H]^+$ calcd for $C_{34}H_{26}B_2N_2O_2$, 517.2180; found, 517.1243.

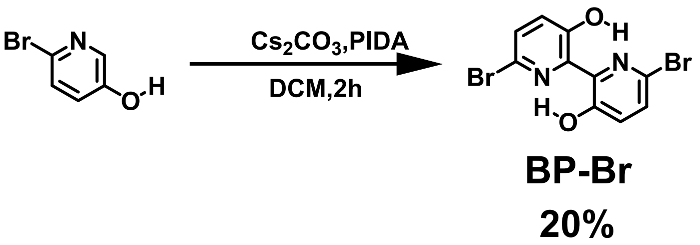

Synthesis of BP-Br

To a stirred solution of the 2-Hydroxy-5-bromopyridine (500 mg, 2.87 mmol) in CH_2Cl_2 (8 mL) were added Cs_2CO_3 (156 mg, 0.48 mmol) and PIDA (309 mg, 0.96 mmol). The mixture was stirred at room temperature for 2 h and then concentrated in vacuo. The residue was purified by column chromatography (petroleum ether/ethyl acetate = 10:1). The resultant was further recrystallized from CH_2Cl_2/hexane (1:4) to obtain pale white crystals (98 mg, 20% yield). ^{1}H NMR (500 MHz，CD_3Cl) δ 12.87 (s, 2H), 7.43 (d, J = 10 Hz, 2H), 7.34 (d, J = 10 Hz, 2H).

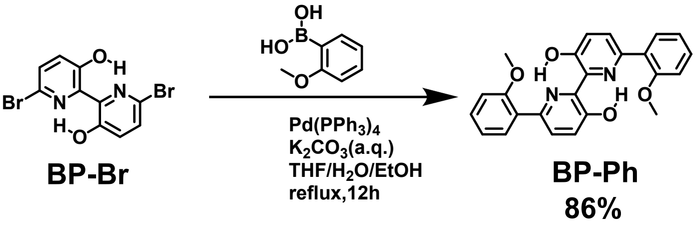

Synthesis of BP-Ph

To a stirred solution of BP-Br (250 mg, 0.72 mmol) in THF (10 mL), C_2H_5OH (2 mL) and water (10 mL) were added dropwise. K_2CO_3 (1.50 g, 10.8 mmol), $Pd(PPh_3)_4$ (25mg, 0.022 mmol), 2-Methoxyphenylboronic acid (264 mg, 1.73 mmol) were added in sequence and the mixture was heated to 70 °C for 12 h. The mixture was cooled to room temperature and the solvent was removed. Then, the product was extracted with CH_2Cl_2 and water. The organic layer was dried over anhydrous $MgSO_4$, filtrated and evaporated using a rotary evaporator. The resultant was recrystallized from CH_2Cl_2/hexane (1: 4) obtain yellow crystals (273 mg, 86% yield). ^{1}H NMR (500 MHz，$CDCl_3$, δ, ppm): 14.91 (s, 2H), 7.74 (d，J = 10 Hz，2H)，7.65 (dd，J_1 = 7.6，J_2 = 1.7 Hz，1H), 7.45 (d，J = 10 Hz，2H), 7.41 – 7.37 (m, 2H), 7.09 (t，J = 7.5 Hz，2H)， 7.05 (d，J = 10 Hz，2H), 3.91 (s, 6H).

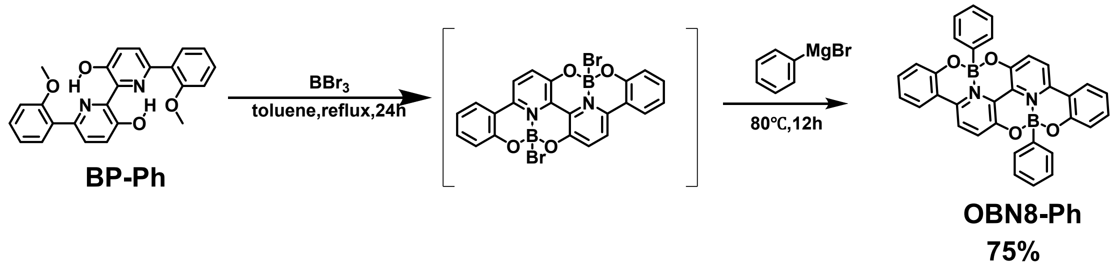

Synthesis of OBN8-Ph

To a stirred solution of BP-Ph (100 mg, 0.23 mmol) in toluene (40 mL), BBr_3 (341 mg, 1.36 mmol) was added dropwise. Next, the solution was sealed and stirred at 110 ºC or further 24 h. A tawny solution was formed. After cooling to room temperature, then, PhMgBr (1 mol/L, 4.1 mL, 4.09 mmol) was added dropwise under N_2 atmosphere. The resulting mixture was stirred at 80 ºC for further 12 h. The mixture was cooled to room temperature and the solvent was removed. Then, the product was extracted with CH_2Cl_2 and saturated ammonium chloride solution. The organic layer was dried over anhydrous $MgSO_4$, filtrated and evaporated using a rotary evaporator. The resultant was recrystallized from CH_2Cl_2/hexane (1: 2) to obtain yellow crystals (92 mg, 75% yield). ^{1}H NMR (500 MHz, $CDCl_3$, δ, ppm): 7.94 (d, J = 10 Hz, 2H), 7.77 (d, J = 10 Hz, 2H), 7.55 (d, J = 5 Hz, 2H), 7.33 (t, J = 10 Hz, 2H), 7.18-6.98 (m, 12H), 6.89 (t, J = 10 Hz, 2H). ^{13}C NMR (500 MHz, $CDCl_3$, δ, ppm): 155.66, 151.20, 141.00, 133.74, 133.23, 131.18, 128.33, 127.66, 127.52,

124.85, 123.76, 120.66, 120.46, 117.49. ^{11}B NMR (500 MHz, $CDCl_3$) δ 6.15 ppm. MALDI-TOF MS *m/z*: $[M + H]^+$ calcd for $C_{34}H_{22}B_2N_2O_4$, 545.1766; found, 544.9012.

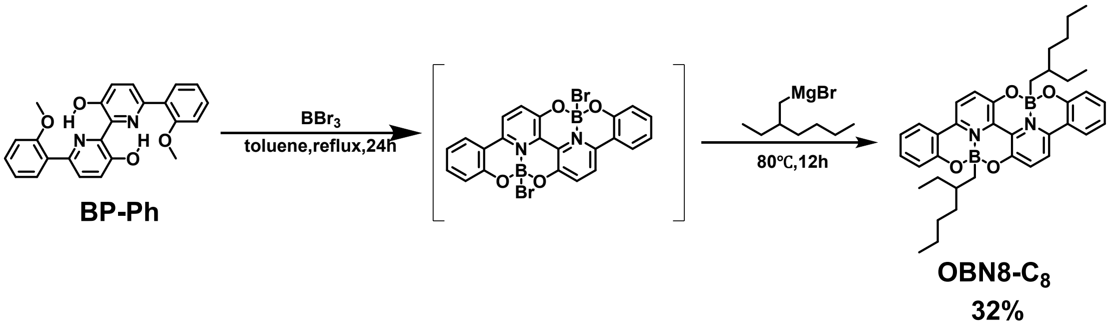

Synthesis of OBN8-C$_8$

To a stirred solution of BP-Ph (100 mg, 0.23 mmol) in toluene (40 mL), BBr_3 (341 mg, 1.36 mmol) was added dropwise. Next, the solution was sealed and stirred at 110 ºC or further 24 h. A tawny solution was formed. After cooling to room temperature, then, (2-Ethylhexyl) magnesium bromide (1 mol/L, 4.1 mL, 4.09 mmol) was added dropwise under N_2 atmosphere. The resulting mixture was stirred at 80 ºC for further 12 h. The mixture was cooled to room temperature and the solvent was removed. Then, the product was extracted with CH_2Cl_2 and saturated ammonium chloride solution. The organic layer was dried over anhydrous $MgSO_4$, filtrated and evaporated using a rotary evaporator. The solid was purified by silica gel column chromatography (petroleum ether/dichloromethane =8: 1). The resultant was recrystallized from CH_2Cl_2/hexane (1: 8) to obtain yellow crystals (45 mg, 32% yield). Anti-isomers are separated, verified by the X-ray crystallography. ^{1}H NMR (500 MHz, $CDCl_3$, δ, ppm): 7.89 (d, J = 10 Hz, 2H), 7.69 (d, J = 10 Hz, 2H), 7.61 (d, J = 5 Hz, 2H), 7.38 (t, J = 10 Hz, 2H), 7.08 (d, J = 10 Hz, 2H), 6.97 (t, J = 10 Hz, 2H), 1.31 (m, 2H), 1.22-0.82 (m, 16H), 0.76 (m, 6H), 0.59 (m, 6H), 0.38 (m, 2H), 0.29 (m, 2H). ^{13}C NMR (500 MHz, $CDCl_3$, δ, ppm): 156.94, 151.92, 140.83, 133.35, 132.29, 127.62, 124.49, 122.92, 120.87, 120.05, 118.08, 35.49, 35.29, 35.33, 35.24, 29.10, 28.86, 28.51, 28.25, 23.18, 23.11, 14.25, 11.13, 10.90. ^{11}B NMR (500 MHz, $CDCl_3$) δ 9.82 ppm. MALDI-TOF MS *m/z*: $[M + H]^+$ calcd for $C_{38}H_{46}B_2N_2O_4$, 617.3644; found, 618.2483.

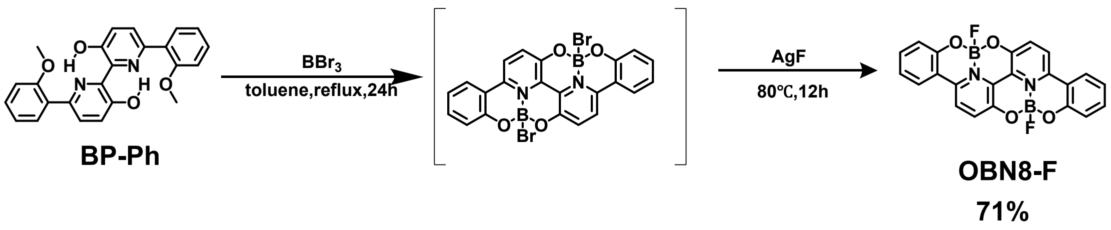

Synthesis of OBN8-F

To a stirred solution of BP-Ph (100 mg, 0.23 mmol) in toluene (40 mL), BBr_3 (341 mg, 1.36 mmol) was added dropwise. Next, the solution was sealed and stirred at 110 ºC or further 24 h. A tawny solution was formed. After cooling to room temperature, then, AgF (519 mg, 4.09 mmol) was added dropwise under N_2 atmosphere. The resulting mixture was stirred at 80 ºC for further 12 h. The mixture was cooled to room temperature and the solvent was removed. Then, the product was extracted with CH_2Cl_2 and saturated ammonium chloride solution. The organic layer was dried over anhydrous $MgSO_4$, filtrated and evaporated using a rotary evaporator. The solid was purified by silica gel column chromatography (petroleum ether/dichloromethane =1: 2). The resultant was recrystallized from CH_2Cl_2/hexane (1: 4) to obtain yellow crystals (69 mg, 71% yield). ^{1}H NMR (500 MHz, DMSO, δ ppm): 8.69 (d, J = 10 Hz, 2H), 8.26 (d, J = 10 Hz, 2H), 8.22 (d, J = 5 Hz, 2H), 7.55 (t, J = 10 Hz, 2H), 7.16 (m, 4H). ^{11}B NMR (500 MHz, $CDCl_3$) δ 1.82 ppm. HRMS (MALDI-TOF) *m/z*: $[M]^+$ calcd for $C_{22}H_{12}B_2F_2N_2O_4$, 428.0951; found, 427.9991.

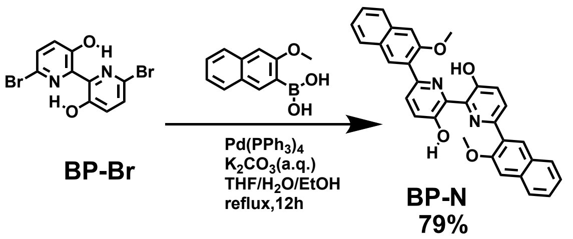

Synthesis of BP-N

To a stirred solution of BP-Br (250 mg, 0.72 mmol) in THF (10 mL), C_2H_5OH (2 mL) and water(10 mL) were added dropwise. K_2CO_3 (1.50 g, 10.8 mmol), $Pd(PPh_3)_4$ (25 mg, 0.022 mmol) , 3-Methoxynaphthalene-2-boronic acid (350.3 mg, 1.73 mmol) were added in sequence and the mixture was heated to 70 °C for 12 h. The mixture was cooled to room temperature and the solvent was removed. Then, the product was extracted with CH_2Cl_2 and water. The organic layer was dried over anhydrous $MgSO_4$, filtrated and evaporated using a rotary evaporator. The resultant was recrystallized from CH_2Cl_2/hexane (1:4) to obtain yellow crystals (285 mg, 79% yield). ^{1}H NMR (500 MHz, $CDCl_3$, δ ppm): 14.94 (s, 2H), 8.08 (s, 2H), 7.87 (d, J = 10 Hz, 2H), 7.80 (d, J = 10 Hz, 4H), 7.50 (d, J = 10 Hz, 4H), 7.39 (t, J = 7.5 Hz, 2H), 7.30 (s, 2H), 4.05 (s, 6H).

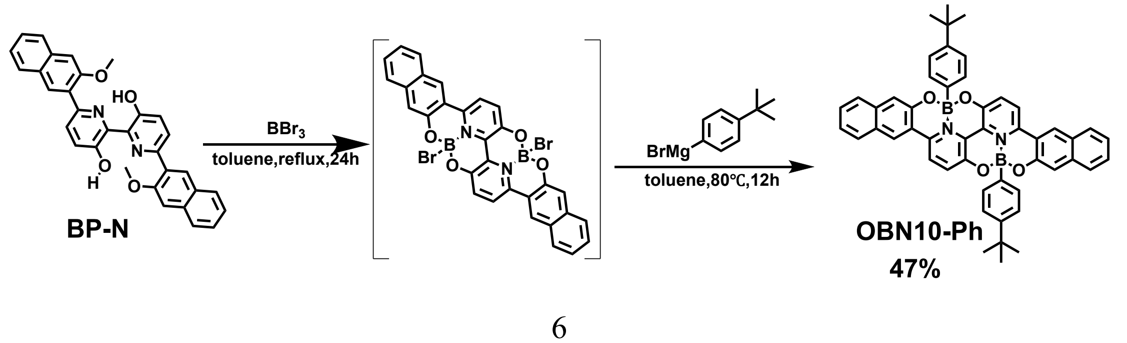

Synthesis of OBN10-Ph

To a stirred solution of BP-N (150 mg, 0.30 mmol) in toluene (60 mL), BBr_3 (450 mg, 1.80 mmol) was added dropwise. Next, the solution was sealed and stirred at 110 ºC or further 24 h. A tawny solution was formed. After cooling to room temperature, then, 4-tert-Butylphenylmagnesium bromide (0.5 mol/L, 10.8 mL, 5.4 mmol) was added dropwise under N_2 atmosphere. The resulting mixture was stirred at 80 ºC for further 12 h. The mixture was cooled to room temperature and the solvent was removed. Then, the product was extracted with CH_2Cl_2 and saturated ammonium chloride solution. The organic layer was dried over anhydrous $MgSO_4$, filtrated and evaporated using a rotary evaporator. The solid was purified by silica gel column chromatography (petroleum ether/dichloromethane = 1: 2). The resultant was recrystallized from CH_2Cl_2/hexane (1:4) to obtain yellow crystals (107 mg, 47% yield). Cis-isomers are separated, verified by the X-ray crystallography. ^{1}H NMR (500 MHz, $CDCl_3$, δ ppm): 8.13 (d, *J* = 10 Hz, 2H), 8.07 (s, 2H), 7.82 (d, *J* = 10 Hz, 2H), 7.67 (dd, *J* = 12.6, 8.3 Hz, 4H), 7.45 (s, 2H), 7.39 (t, *J* = 8.0 Hz, 2H), 7.24 (d, *J* = 5 Hz, 2H), 7.04 (s, 8H), 1.16 (s, 18H). ^{13}C NMR (500 MHz, $CDCl_3$, δ ppm): 151.98, 151.51, 150.17, 140.62, 136.82, 133.11, 131.09, 128.51, 128.45, 128.13, 128.05, 126.50, 126.48, 125.97, 124.84, 124.82, 124.33, 124.14, 119.47, 115.16, 34.31, 31.30, 31.28, 31.25. ^{11}B NMR (500 MHz, $CDCl_3$) δ 6.26 ppm. MALDI-TOF MS *m/z*: $[M]^+$ calcd for $C_{50}H_{42}B_2N_2O_4$, 756.3331; found, 756.5713.

3. X-ray crystallography

X-ray crystallography data were collected on Bruker D8 Venture Diffractometer. Single crystals of OBN8-C8 and OBN10-Ph were obtained by slow diffusion of MeOH to the CH_2Cl_2 solutions for 20-30 days. The diffraction data was collected in 293 K for OBN8-C8 and 150 K for OBN10-Ph on a Bruker D8 Venture single crystal diffractometer with Mo Kα (λ = 0.71703 Å) for OBN8-C8 and Cu Kα (λ = 1.54178 Å) for OBN10-Ph.

Table S1. Crystallographic data of OBN8-C8 (CCDC: 2472866)

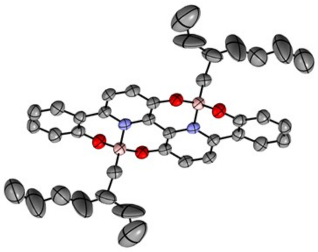

Name	OBN10-Ph
Formula	$C_{19} H_{23} B N O_2$
Radiation	MoKα (λ = 0.71703 Å)
Formula weigh	308.19
Temperature	293 K
Crystal system	monoclinic
Space group	*I 1 2/a 1*
a(Å)	18.654(3)
b(Å)	5.8046(9)
c(Å)	31.238(6)
α(°)	90
β(°)	95.121(18)
γ(°)	90
Volume (Å^3)	3369.0(10)
Z	8
Density (g/cm^3)	1.215
μ/mm^{-1}	0.077
F(000)	1320
h, k, l	23, 6, 42
θ_{max}	22.631
Completeness to theta	0.983
Max. and min. transmission	1.000, 0.563
R_1 (I>2σ(I))	0.1212
wR_2 (I>2σ(I))	0.3022

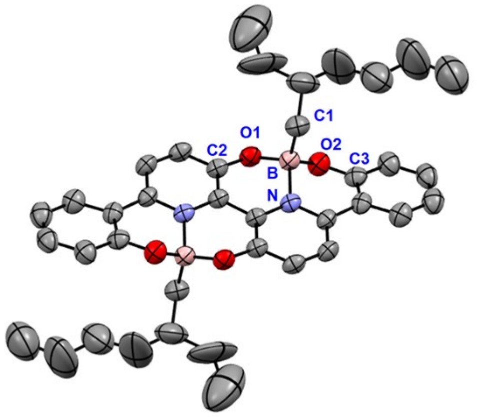

Figure S2. Selected bond length (Å) and angles (°) of OBN8-C8: B-O1, 1.468, B-O2, 1.454, B-N, 1.625, B-C1, 1.599, O1-C2, 1.328, O2-C3, 1.345, O1-B-N, 108.25, O2-B-N, 105.85, O1-B-C1, 113.36, O2-B-C1, 116.74, B-O1-C2, 119.47, B-O2-C3, 116.91.

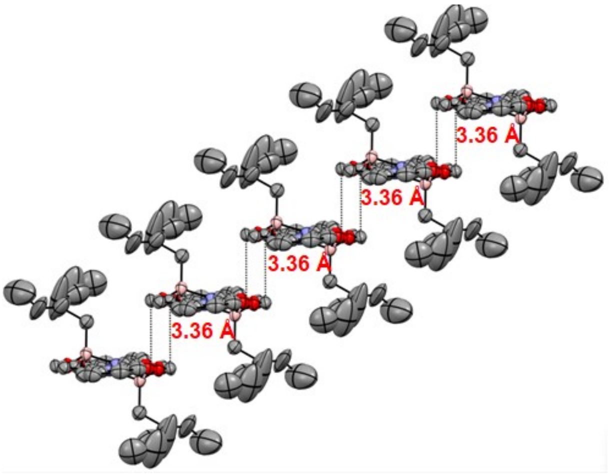

Figure S3. Slipped stacking of OBN8-Ph with layer-to-layer distance of 3.36 Å.

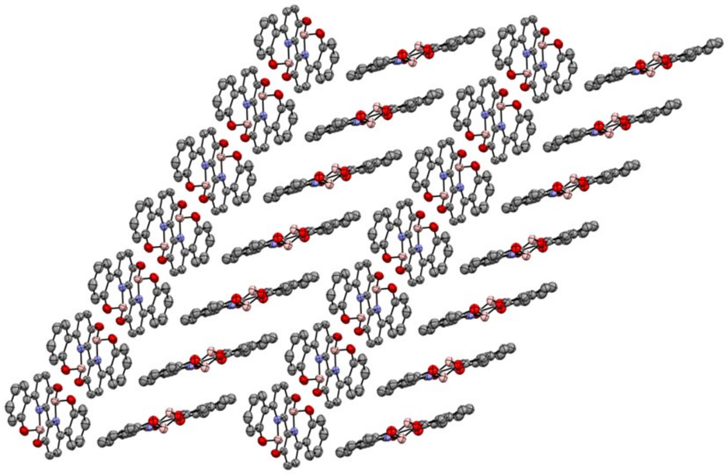

Figure S4. long-distance stacking mode of OBN8-C8 in single crystal.

Table S2. Crystallographic data of OBN10-Ph (CCDC: 2472869)

Name	OBN10-Ph•0.5CH_2Cl_2
Formula	$C_{50.5}$ H_{43} B_2 N_2 O_4Cl
Radiation	CuKα (λ = 1.54178 Å)
Formula weigh	789.94
Temperature	150 K
Crystal system	triclinic
Space group	*P*
a(Å)	14.1640 (3)
b(Å)	17.1362 (4)
c(Å)	18.8082 (4)
α(°)	97.5230 (10)
β(°)	100.3440 (10)
γ(°)	113.7920 (10)
Volume (Å^3)	4002.98 (16)
Z	4
Density (g/cm^3)	1.326
μ/mm^{-1}	1.244
F(000)	1676
h, k, l	17, 21, 23
θ_{max}	74.531
Completeness to theta	0.983
Max. and min. transmission	0.754, 0.663
R_1 (I>2σ(I))	0.0549
wR_2 (I>2σ(I))	0.1585

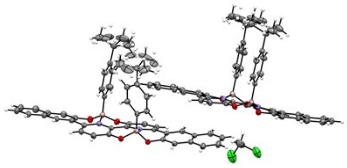

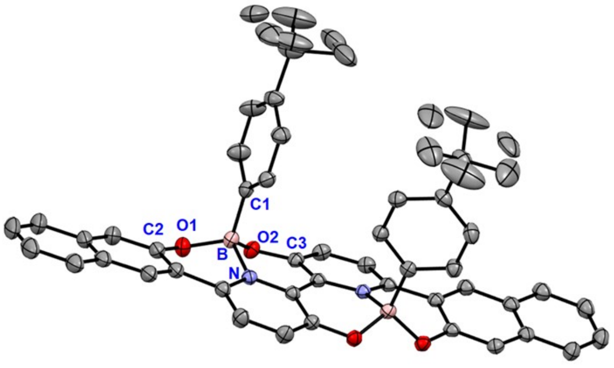

Figure S5. Selected bond length (Å) and angles (°) of OBN10-Ph: B-O1, 1.445, B-O2, 1.476, B-N, 1.609, B-C1, 1.610, O1-C2, 1.353, O2-C3, 1.327, O1-B-N, 107.59, O2-B-N, 107.98, O1-B-C1, 116.27, O2-B-C1, 111.14, B-O1-C2, 116.79, B-O2-C3, 118.18.

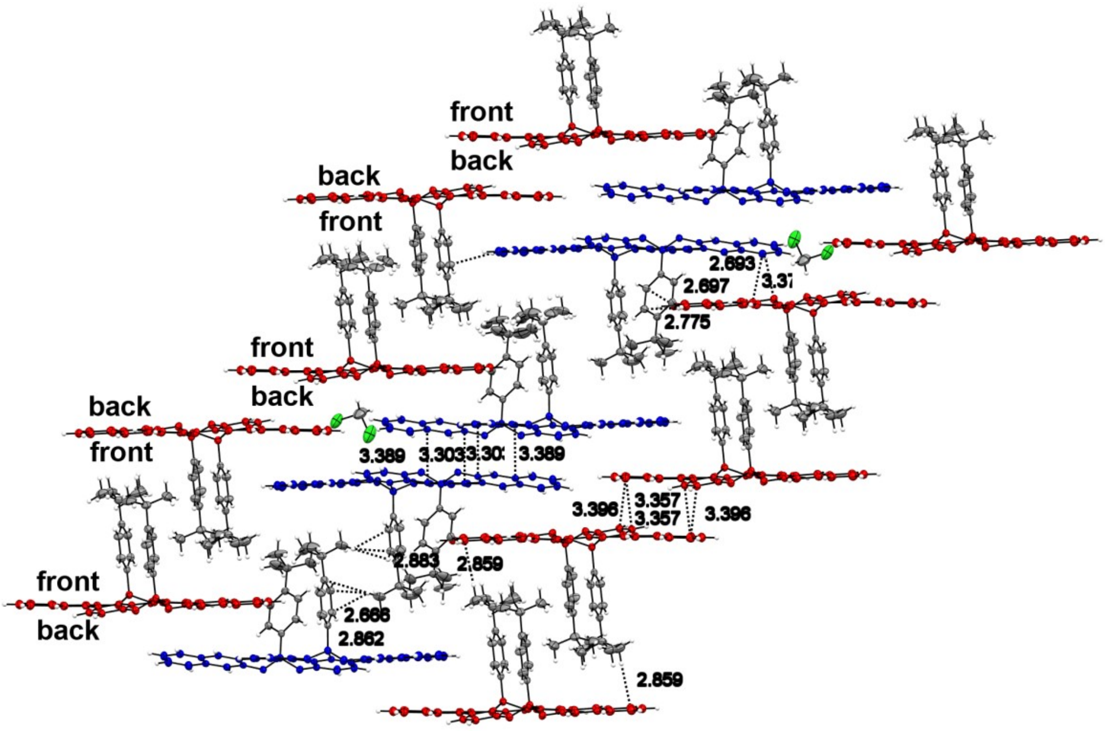

Figure S6. Packing and interactions of OBN10-Ph in single crystals.

4. Stability measurements

The stability of the O-B←N perturbed PAHs were estimated by thermogravimetric analysis (TGA) and the monitoring the UV-Vis absorption spectra. The TGA was tested under N_2 atmosphere and the UV-Vis absorption spectra were monitored in air or N_2 atmosphere. The half-life time of OBN8-C8 was calculated by fitting the absorption strength at 456 nm *vs* the time. The gradually increased absorption of OBN8-F can be related to its strong aggregation in solution and the aggregates were slowly dissolved to the solution, leading to increased concentration.

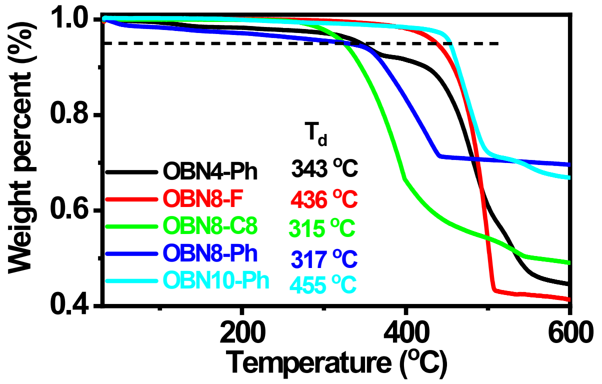

Figure S7. TGA curves of the three O-B←N perturbed PAHs.

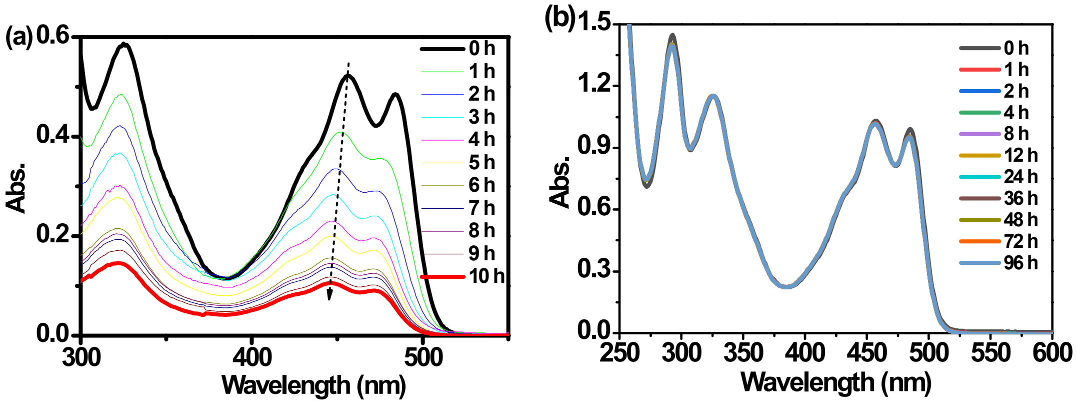

Figure S8. UV-vis absorption spectra of OBN8-C8 in $CHCl_3$ under air (a) and N_2 atmosphere (b).

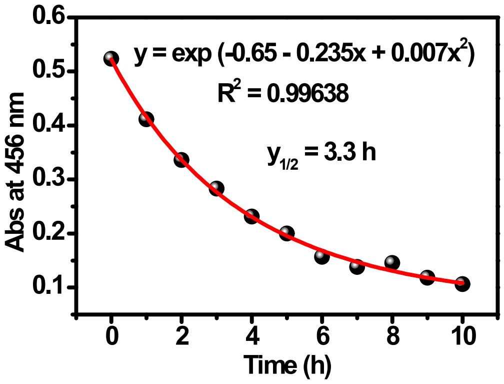

Figure S9. Strength decay of absorption at 456 nm and determination of half-life time of OBN8-C8 in air.

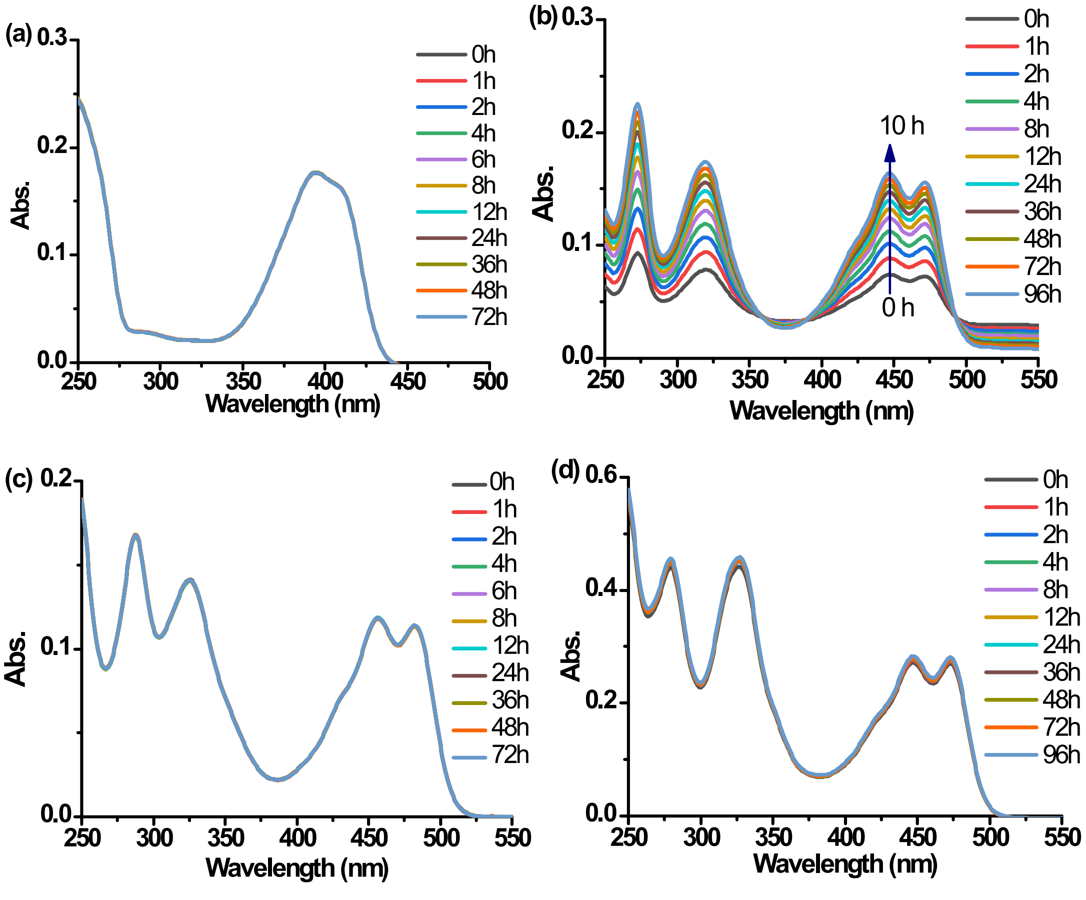

Figure S10. UV-vis absorption spectra of OBN4-Ph (a), OBN8-F (b), OBN8-Ph (c), and OBN10-Ph (d) in $CHCl_3$ under air.

5. Theoretical simulations.

Molecular geometry optimization, HOMO/LUMO analysis, nuclear independents chemical shifts (NICS), and two-dimensional iso-chemical shielding surface (2D-ICSS), the electrostatic potential distribution maps were performed using Gaussian 16 at the B3LYP/6-31G(d,p) level. [1] Electronic transitions and oscillator strengths was performed using Gaussian 16 at the B3LYP/6-31G* TD level. Anisotropy of the induced current density (ACID) plot was calculated via the method developed by Herges. [2] The ESP area distributions were calculated using Multiwfn 3.8. [3] Analysis and visualization of the results were done with the Multiwfn 3.8 and py.Aroma 4 software. [4] [5]

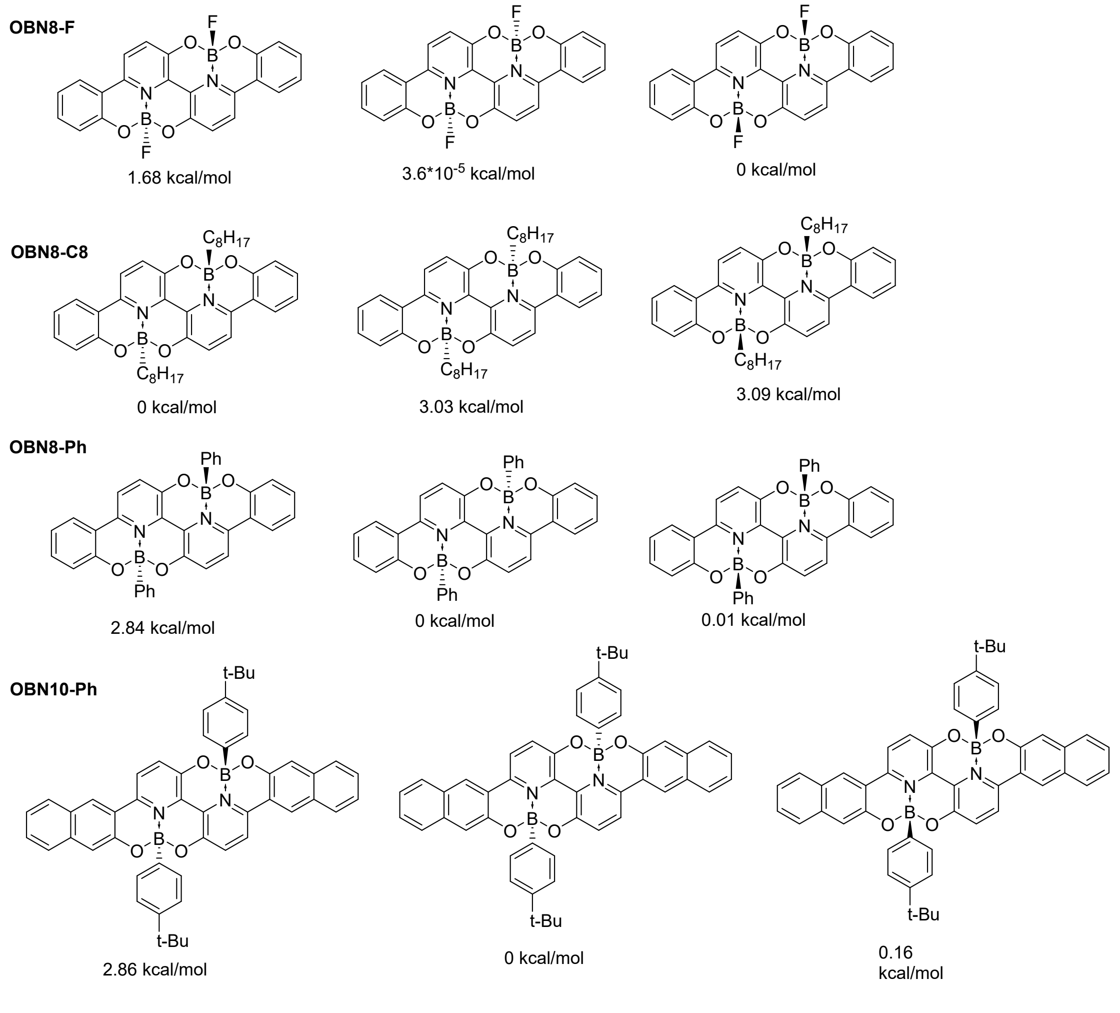

Figure S11. Relative electron energy of anti- and cis- isomers for OBN8-F, OBN8-C8, OBN8-Ph, and OBN10-Ph.

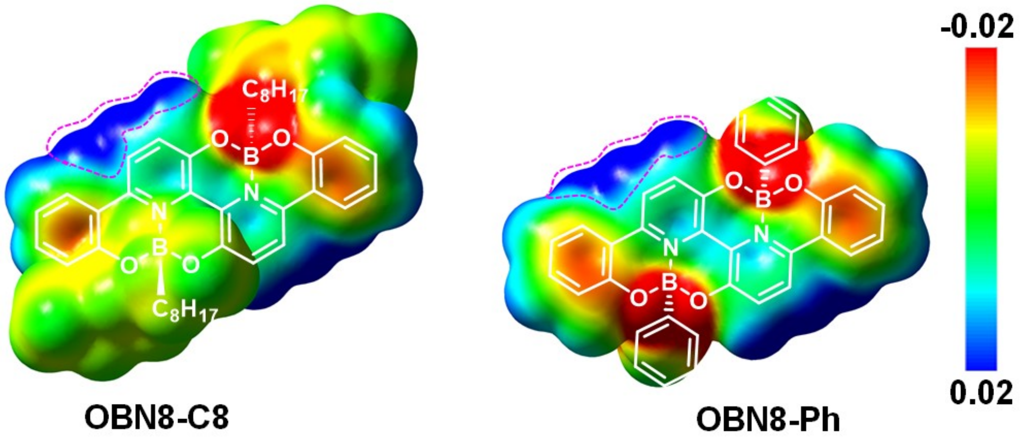

Figure S12. ESP maps of OBN8-C8 and OBN8-Ph

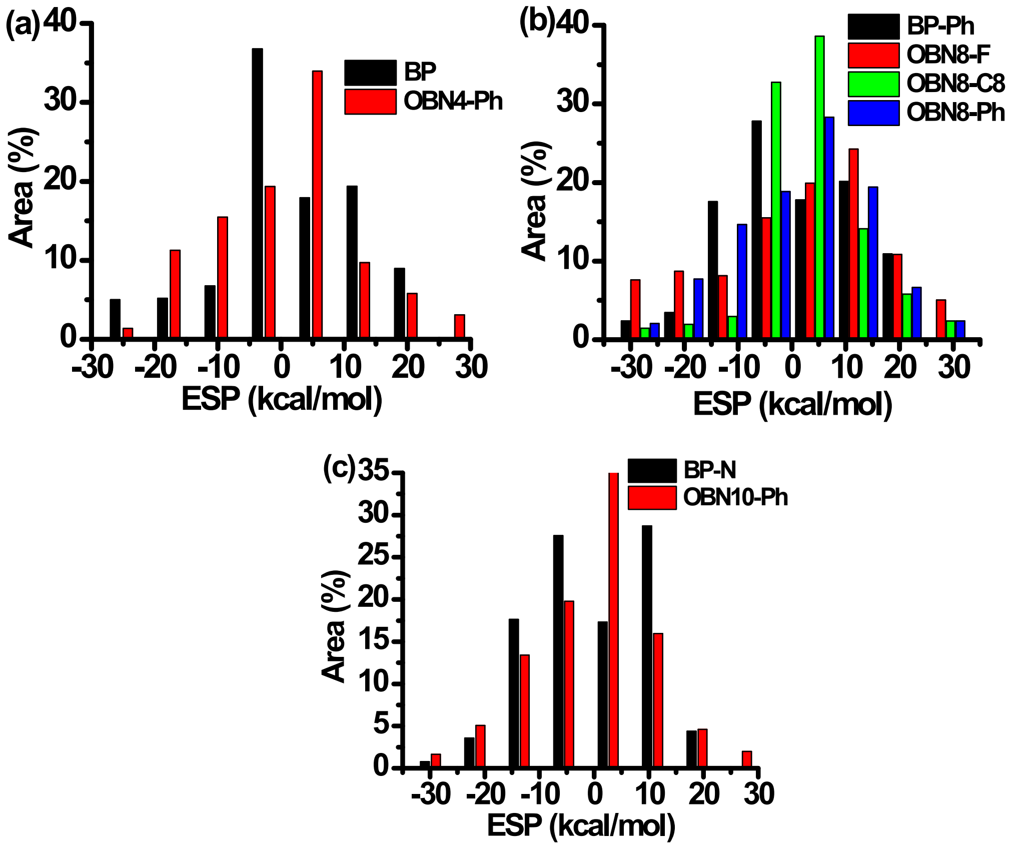

Figure S13. ESP area distribution of BP/OBN4-Ph (a), BP-Ph/OBN8-F/OBN8-C8/OBN8-Ph (b), and BP-N/OBN10-Ph (c)

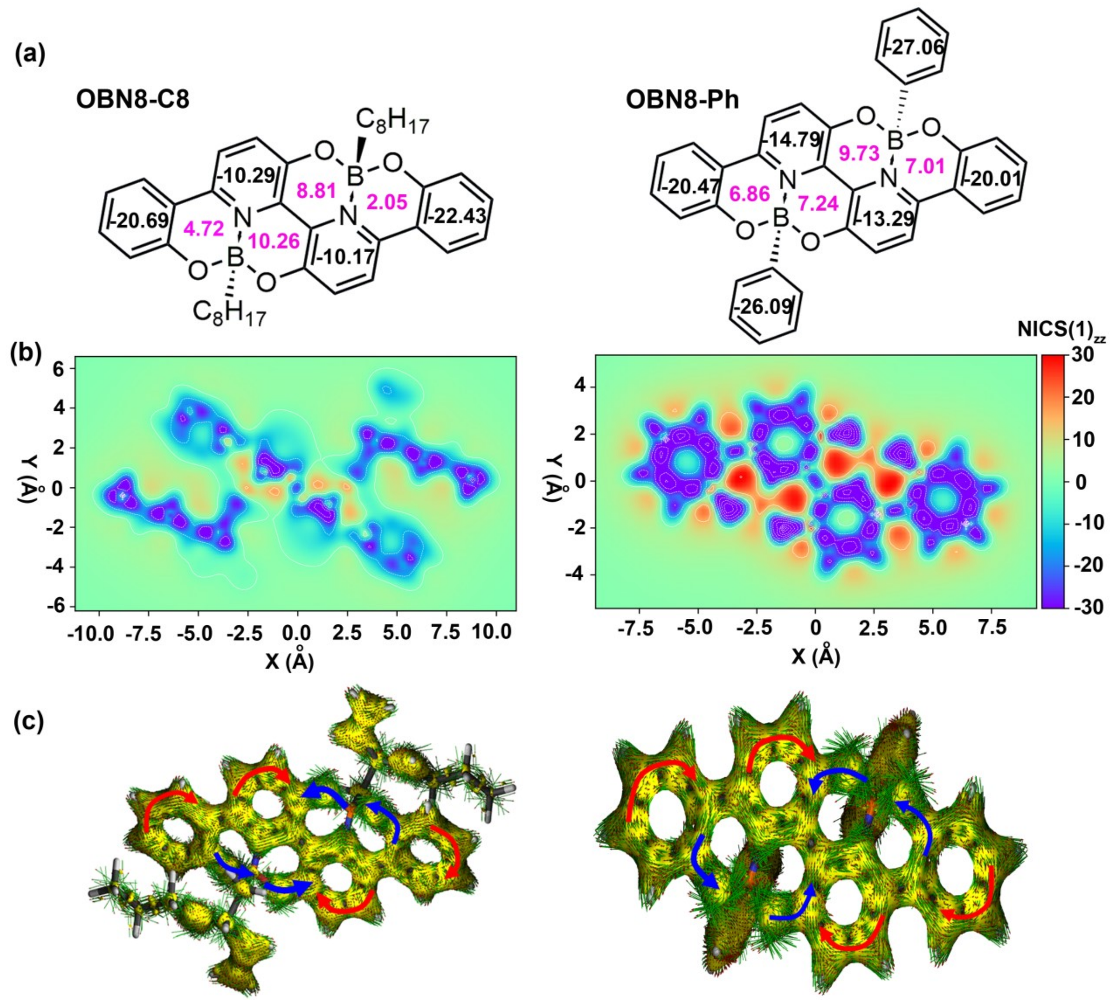

Figure S14. $NICS(1)_{zz}$ plots (b), 2D-ICSS plots (b), and AICD plots (c) of OBN8-C8 and OBN8-Ph.

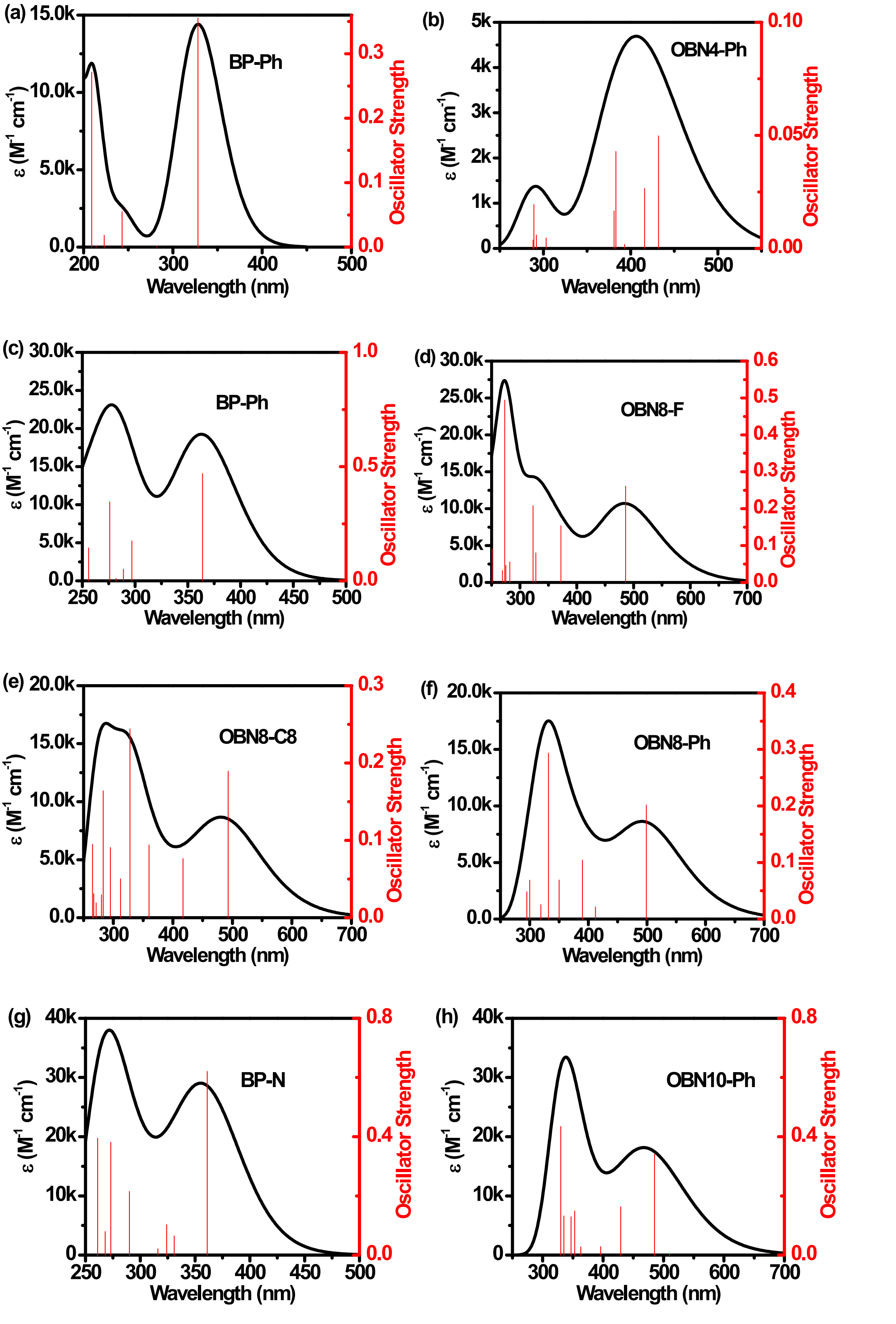

Figure S15. Simulated absorption spectra and the oscillator strengths (*f*) of the three precursors (a, c, and g) and five O-B←N perturbed PHAs (b, d, e, f, and h).

Table S3. Major electronic transitions of BP simulated by TDDFT methods.

Excited state	Energy(eV)	Wavelength(nm)	Osc. Strength	Major contributions
1	3.7765	328.31	0.3556	H→L(97.2 %)
5	5.0985	243.18	0.055	H→L+2(75.6%), H-3→L(20.6 %)
11	5.9093	209.81	0.2716	H-1→L+1(36.3%), H-3→L(34.4%), H→L+2(14.1%) H-1→L+3(10.6%)
17	6.7058	184.89	0.527	H-3→L+2(37.3%), H-1→L+3(28.9%), H-5→L+1(18.1%) H-1→L+1(5.3%), H-8→L(4.1 %)
19	6.9309	178.89	0.0421	H-3→L+2(49.8%), H-1→L+3(25.7%), H-5→L+1(21.1%)

Table S4. Major electronic transitions of BP-Ph simulated by TDDFT methods.

Excited state	Energy(eV)	Wavelength(nm)	Osc. Strength	Major contributions
1	3.4081	363.8	0.4698	H→L(96.6 %)
4	4.1729	297.12	0.1744	H→L+2(80.1%), H-2→L(13.8 %）
8	4.4895	276.7	0.3456	H-1→L+1(82.9%) H-2→L+2(7.1 %)
10	4.8416	256.08	0.1447	H→L+4(30.7%),-2→L+2(27.2%) H-3→L+1(18.2%),H-1→L+1(11.0%)
16	5.0869	243.73	0.0637	H-5→L(52.4%) H→L+4(27.3 %) H-2→L+2(10.0%) H-4→L(3.6%)
17	5.1204	242.4	0.0798	H-3→L+1(70.8%) H-2→L+2(19.6 %)

Table S5. Major electronic transitions of BP-N simulated by TDDFT methods.

Excited state	Energy(eV)	Wavelength(nm)	Osc. Strength	Major contributions
1	3.4304	361.43	0.6207	H→L(93.8 %)
5	3.8211	324.48	0.1028	H-→L+2(74.6 %), H-2→L(20.4 %)
12	4.2679	290.51	0.2145	H-3→L+1(62.4 %), H-5→L(15.3 %) H-2→L+2(10.2 %), H-1→L+3(2.3 %)
15	4.5342	273.44	0.381	H→L+4(68.0 %), H-4→L(18.5 %) H-3→L+1(4.1 %)
16	4.6181	268.47	0.0795	H-4→L(60.9 %), H→L+4(20.5 %) H-5→L(10.3 %)
18	4.7494	261.05	0.3949	H-4→L+2(38.3 %), H-1→L+3(25.3 %) H-2→L+4(13.4 %), H-3→L+1(8.5 %) H-6→L+1(5.3 %)

Table S6. Major electronic transitions of OBN4-Ph simulated by TDDFT methods.

Excited state	Energy(eV)	Wavelength(nm)	Osc. Strength	Major contributions
1	2.8719	431.72	0.0498	H→L(94.5 %)
3	2.9824	415.71	0.0265	H-2→L(96.4 %)
7	3.2332	383.47	0.0429	H-6→L(82.1 %), H-5→L(12.7 %)
9	3.2537	381.05	0.0166	H-8→L(93.5 %)
15	4.2905	288.98	0.0194	H-10→L(72.5 %) H-3→L+1(12.3 %) H-11→L(6.5 %)

Table S7. Major electronic transitions of OBN8-F simulated by TDDFT methods.

Excited state	Energy(eV)	Wavelength(nm)	Osc. Strength	Major contributions
1	2.5506	486.1	0.261	H→L(98.2 %)
3	3.3356	371.7	0.1538	H-2→L(96.2 %)
7	3.8321	323.54	0.2081	H-1→L+1(71.8 %) H→L+2(18.5 %)
13	4.5332	273.5	0.4941	H-3→L+1(48.0 %) H-2→L+2(23.5 %) H-4→L(14.6 %) H→L+2(5.9 %)
17	4.936	251.16	0.091	H-1→L+3(88.7 %) H-2→L+2(3.2 %)
19	5.2388	236.66	0.2262	H-7→L(70.0 %) H-6→L(9.2 %) H-4→L+2(8.8 %) H-5→L+1(3.3 %)

Table S8. Major electronic transitions of OBN8-C8 simulated by TDDFT methods.

Excited state	Energy(eV)	Wavelength(nm)	Osc. Strength	Major contributions
1	2.514	493.17	0.1894	H→L(97.9 %)
5	3.44	360.42	0.0939	H-1→L+1(95.4 %)
8	3.7738	328.54	0.244	H→L+2(90.4 %)
12	4.1953	295.53	0.0907	H-2→L+2(88. %) H-1→L+3(3.3 %)
13	4.378	283.15	0.164	H-3→L+1(88.6 %) H-12→L(2.5 %)
20	4.6762	265.14	0.0948	H-8→L(52.9 %), H-9→L(19.3 %) H-1→L+3(10.3 %) H-3-→L+2(5.5 %) H-10→L(2.6 %)

Table S9. Major electronic transitions of OBN8-Ph simulated by TDDFT methods.

Excited state	Energy(eV)	Wavelength(nm)	Osc. Strength	Major contributions
1	2.4860	499.36	0.1931	H→L(98.0 %)
6	3.1693	391.20	0.1342	H-5→L(98.9%)
9	3.5818	346.15	0.0685	H-1→L+1(96.2 %)
11	3.7333	332.11	0.2350	H→L+2(91.3%)
13	3.9291	315.55	0.0301	H-2→L+1(94.0%)
19	4.1748	296.98	0.0446	H-3→L+2(90.5 %)

Table S10. Major electronic transitions of OBN10-Ph simulated by TDDFT methods.

Excited state	Energy(eV)	Wavelength(nm)	Osc. Strength	Major contributions
1	2.4318	519.84	0.0000	H→L(97.0 %)
3	2.5996	476.94	0.3413	H-2→L(95.1%)
5	2.9112	425.89	0.1561	H-4→L(97.8 %)
14	3.5146	352.77	0.1486	H-2→L+2(72.3%) H-3→L+1(15.9 %)
17	3.7364	331.83	0.0509	H-8→L(80.2 %)
18	3.8099	324.07	0.4829	H-5→L+1(87.4 %)

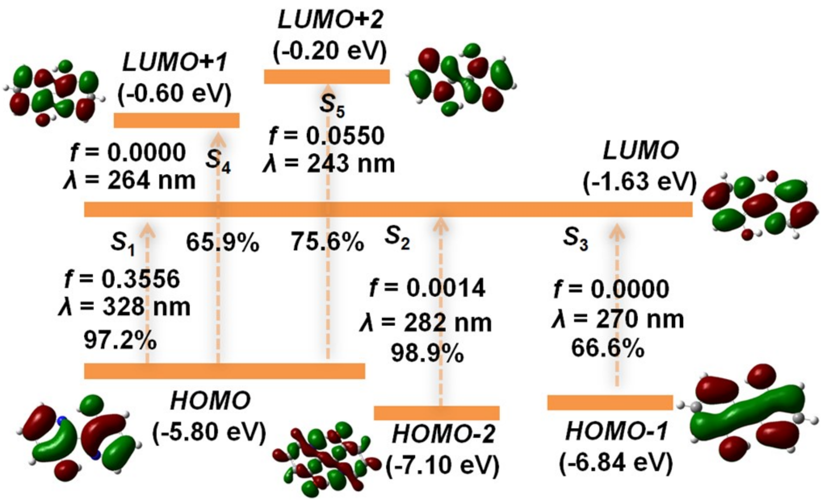

Figure S16. The electronic transitions of BP from S0 to S1, S2, S3, S4, and S5.

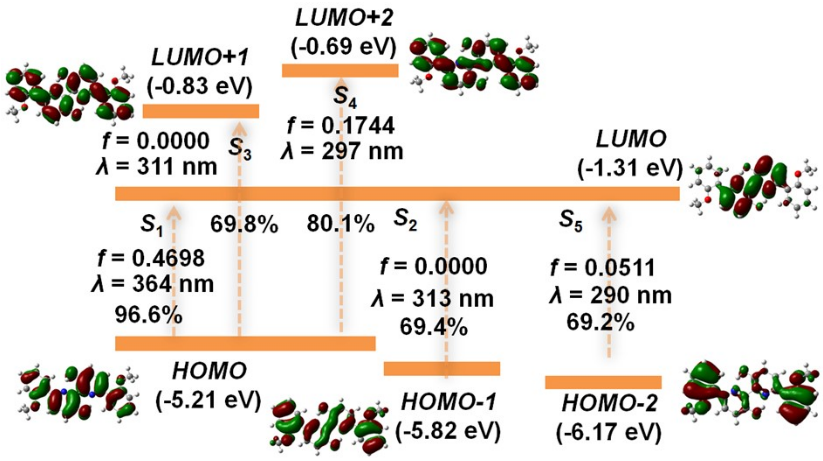

Figure S17. The electronic transitions of BP-Ph from S0 to S1, S2, S3, S4, and S5.

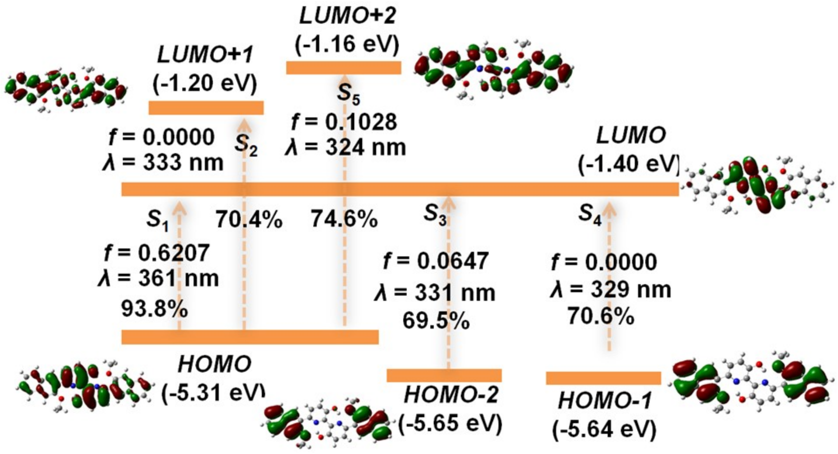

Figure S18. The electronic transitions of BP-N from S0 to S1, S2, S3, S4, and S5.

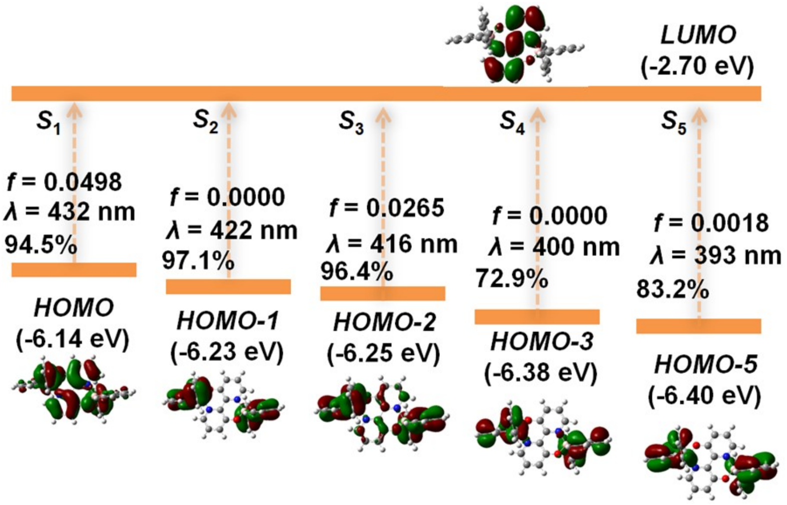

Figure S19. The electronic transitions of OBN4-Ph from S0 to S1, S2, S3, S4, and S5.

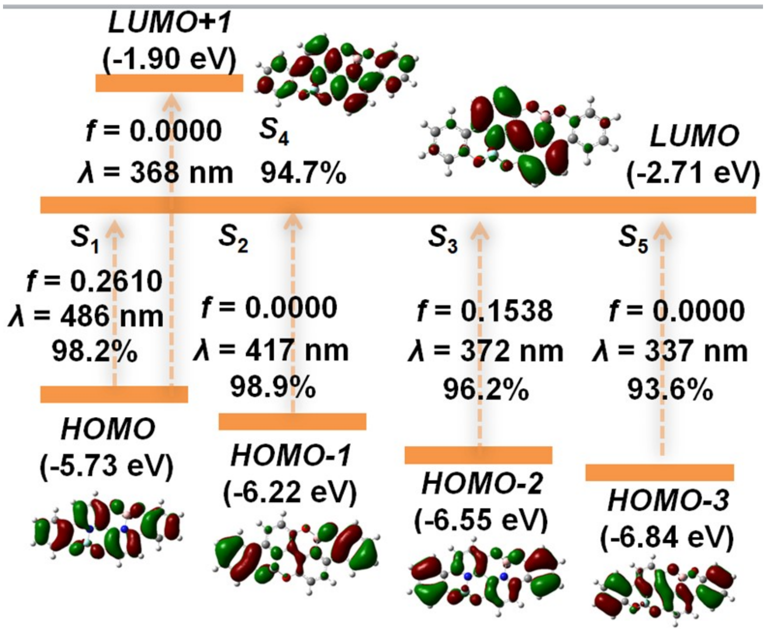

Figure S20. The electronic transitions of OBN8-F from S0 to S1, S2, S3, S4, and S5.

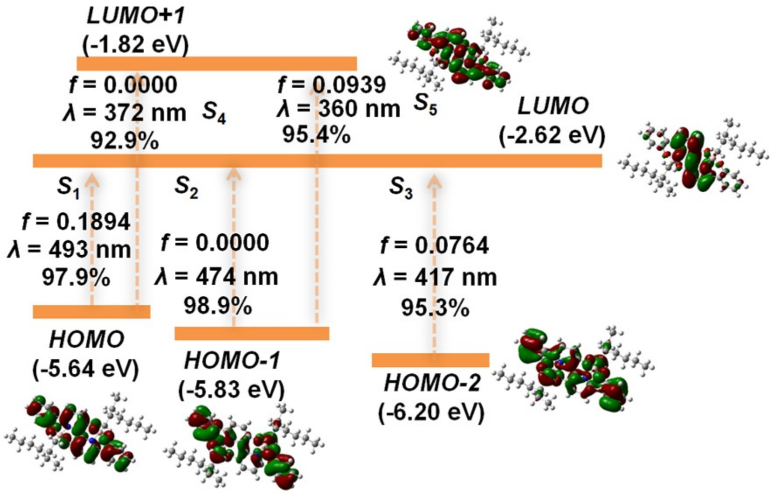

Figure S21. The electronic transitions of OBN8-C8 from S0 to S1, S2, S3, S4, and S5.

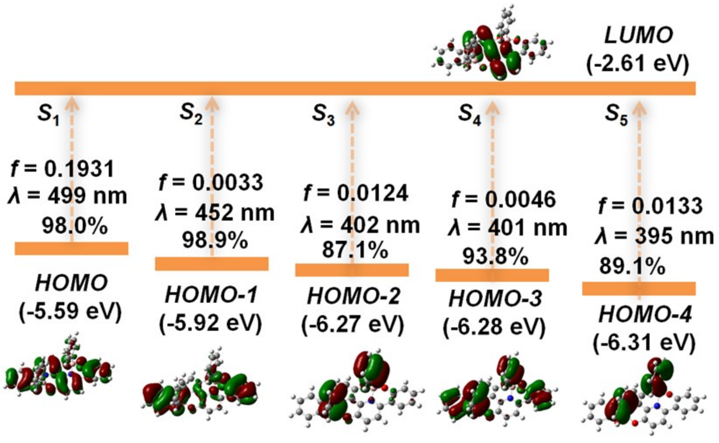

Figure S22. The electronic transitions of OBN8-Ph from S0 to S1, S2, S3, S4, and S5.

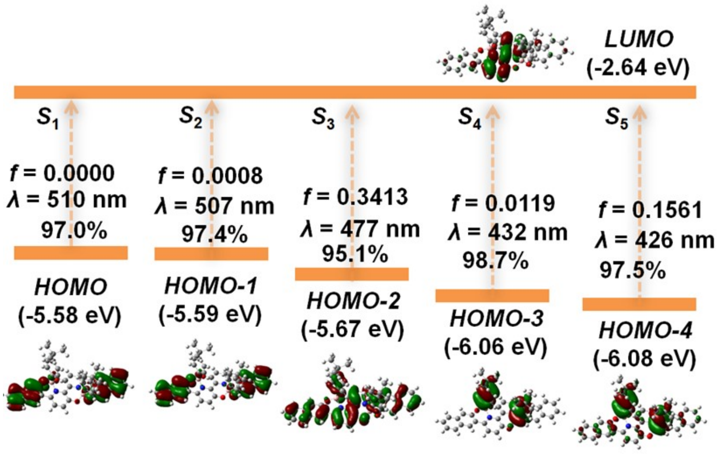

Figure S23. The electronic transitions of OBN10-Ph from S0 to S1, S2, S3, S4, and S5.

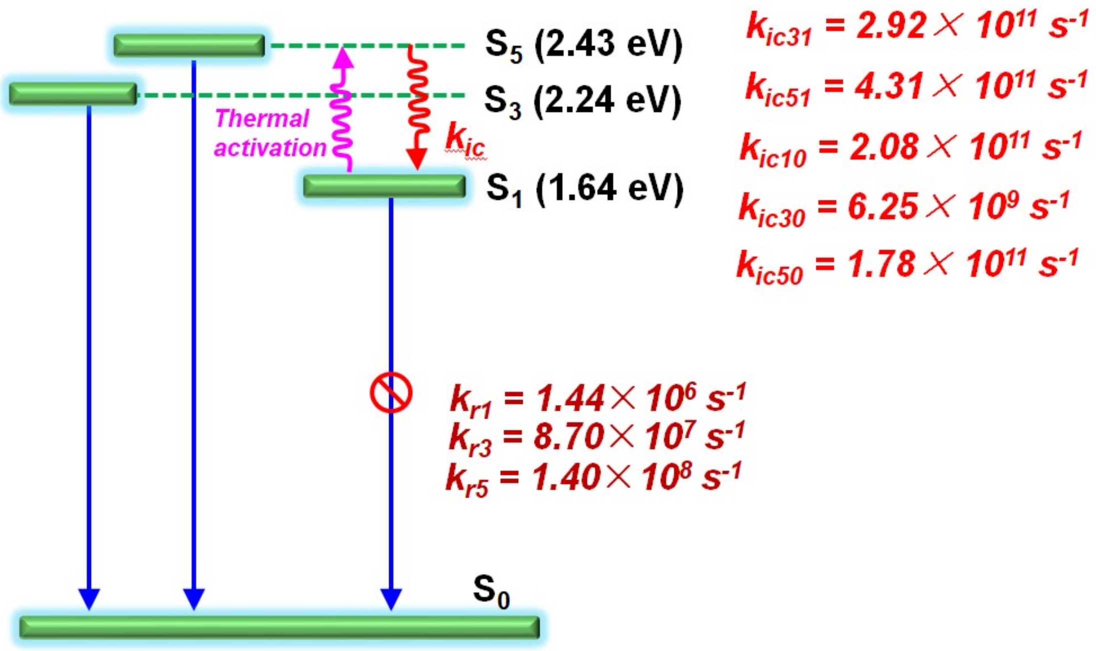

Figure S24. The calculated photophysical processes of OBN10-Ph. k_{ic}: the internal conversion rates; k_{r}: the radiative decay rates.

6. Photophysical properties

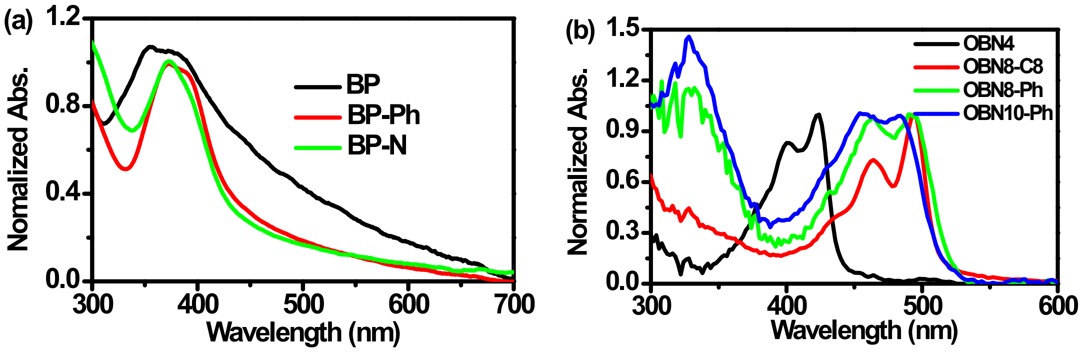

Figure S25. Film absorption of the three precursors (a) and the four O-B←N embedded PAHs (b).

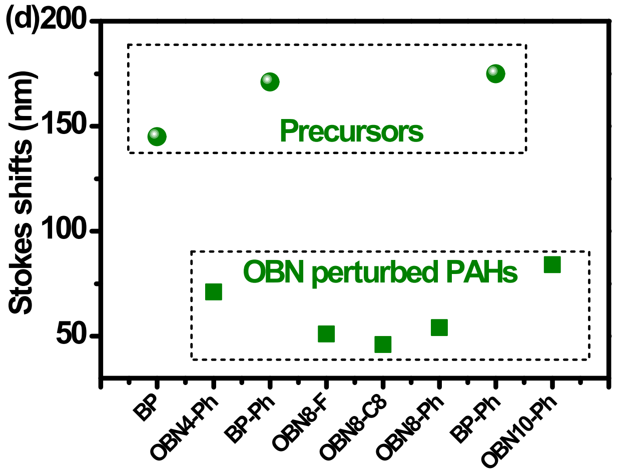

Figure S26. Stokes shifts of the three precursors and five O-B←N perturbed PAHs

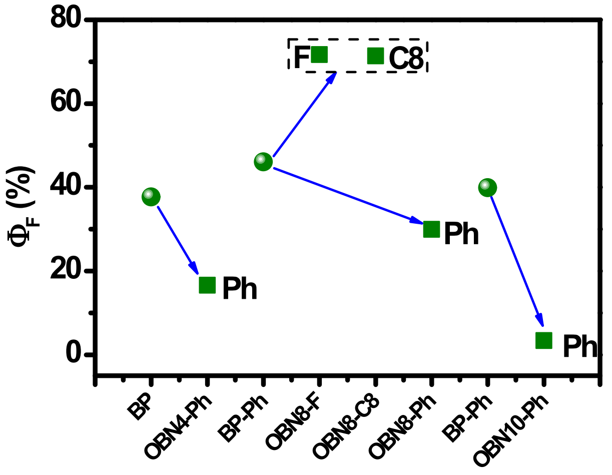

Figure S27. Fluorescence quantum yields (Φ) of the three precursors and five O-B←N perturbed PAHs. The marks are the substituents.

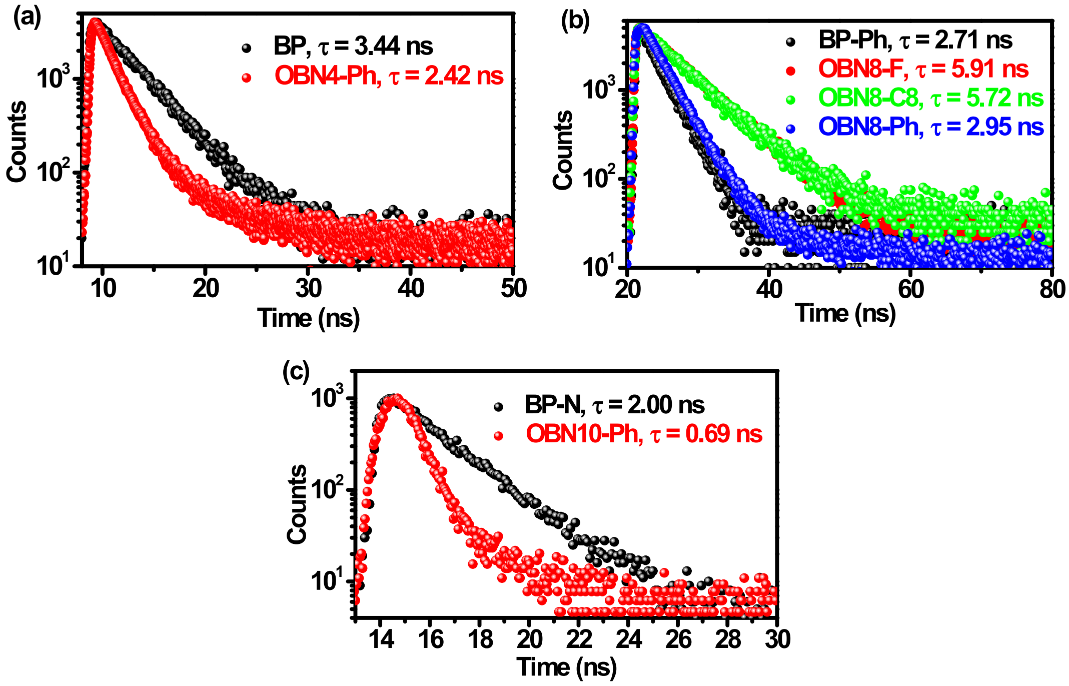

Figure S28. Transient decay fluorescence spectra of BP and OBN4-Ph (a), BP-Ph, OBN8-F, OBN8-C8, and OBN8-Ph (b), and BP-N and OBN10-Ph (c).

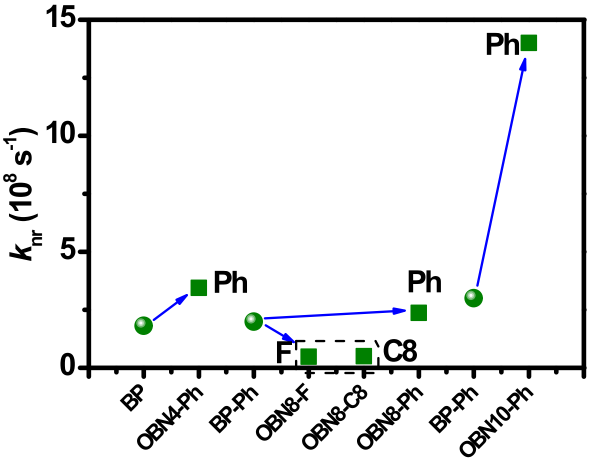

Figure S29. Non-radiative decay rates of the three precursors and five O-B←N perturbed PAHs. The marks are the substituents.

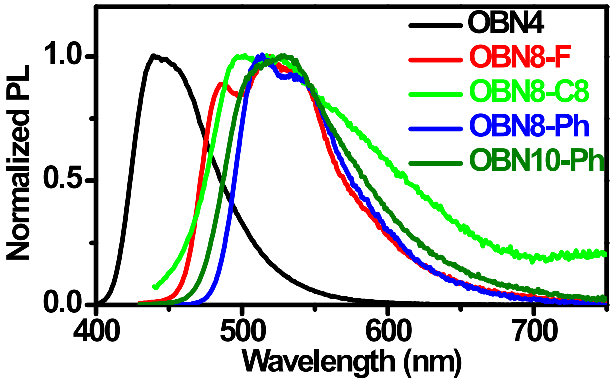

Figure S30. Fluorescence spectra of the five O-B←N embedded molecules distributed in PMMA films (1: 10).

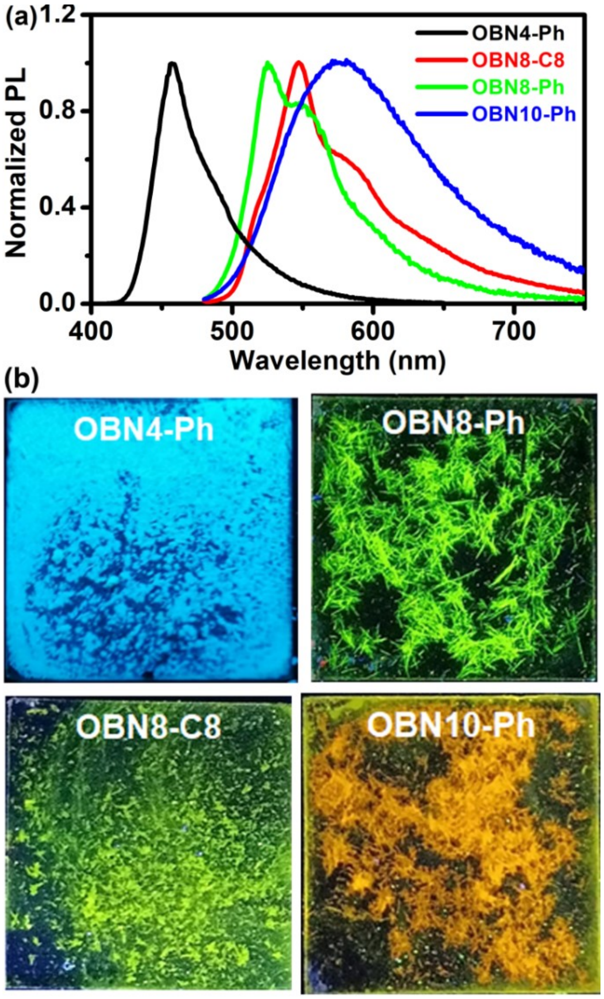

Figure S31. Fluorescence spectra of OBN4-Ph, OBN8-C8, OBN8-Ph, and OBN10-Ph in crystals（a）and photos of the four crystals under 365 nm ultraviolet irradiation (b). Crystal fluorescence of OBN8-F was not obtained due to the poor solubility in common solvents, prohibiting the preparation of crystals from solutions.

Table S11. Fluorescence quantum yields of the five O-B←N perturbed PHAs in solutions, PMMA films, and crystals.

Φ_F	OBN4-Ph	OBN8-F	OBN8-C8	OBN8-Ph	OBN10-Ph
Solution(%)	16.50	71.67	71.38	29.94	3.38
PMMA Film(%)	45.50	51.04	23.64	29.90	4.25
Crystal(%)	45.00	-	0.97	13.65	8.31

Notes: Crystal fluorescence of OBN-F was not obtained due to the poor solubility in common solvents, prohibiting the preparation of crystals from solutions.

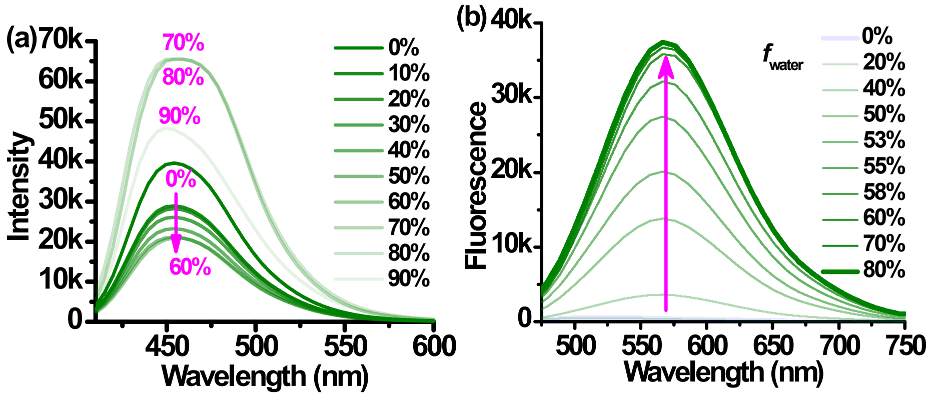

Figure S32. Fluorescence spectra of OBN4-Ph (a) and OBN10-Ph (b) in THF/H_2O mix solvents with different water fractions.

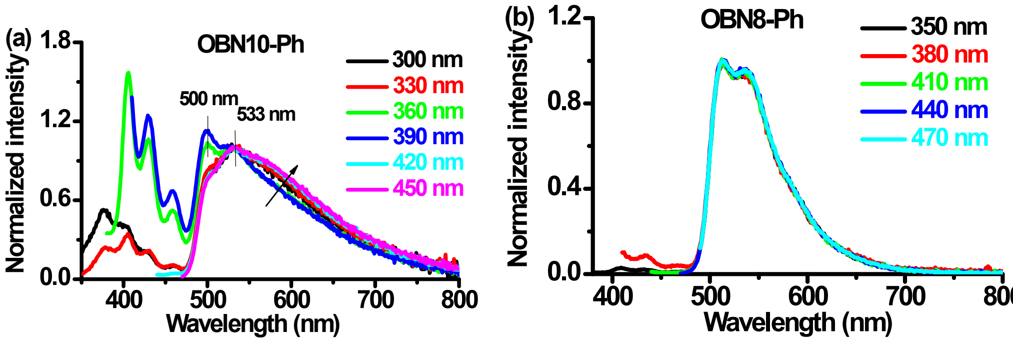

Figure S33. Excitation-wavelength-dependent fluorescence spectra of OBN10-Ph (a) and OBN8-Ph (b).

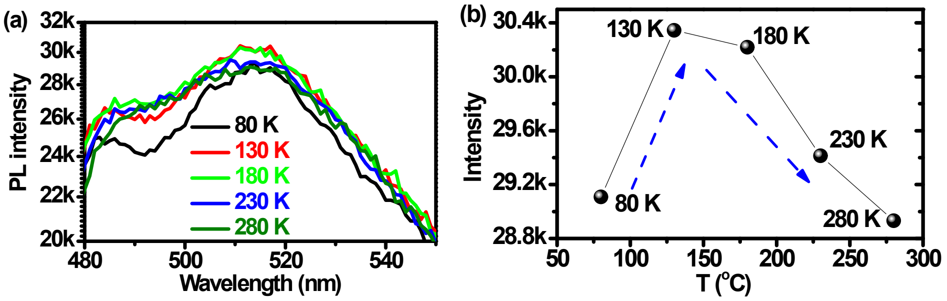

Figure S34. Fluorescence spectra of OBN10-Ph at different temperatures (a) and the plots of maximum FL intensity *vs* temperatures (b). The sample was prepared by distributing OBN10-Ph in PMMA film (1.0 wt%).

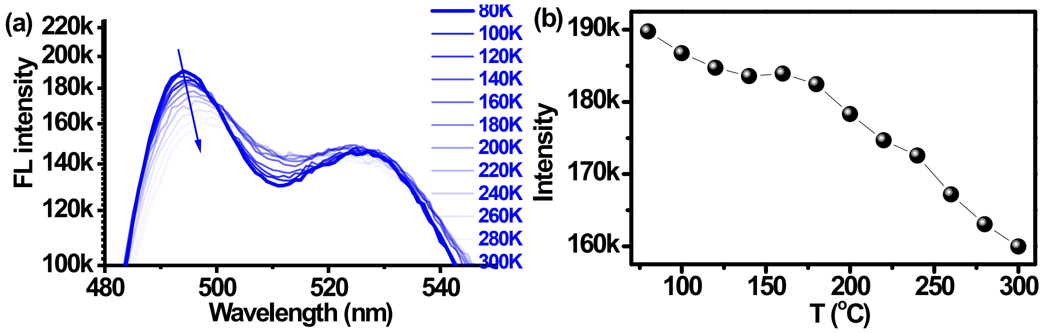

Figure S35. Fluorescence spectra of OBN8-Ph at different temperatures (a) and the plots of maximum FL intensity *vs* temperatures (b). The sample was prepared by distributing OBN10-Ph in PMMA film (1.0 wt%).

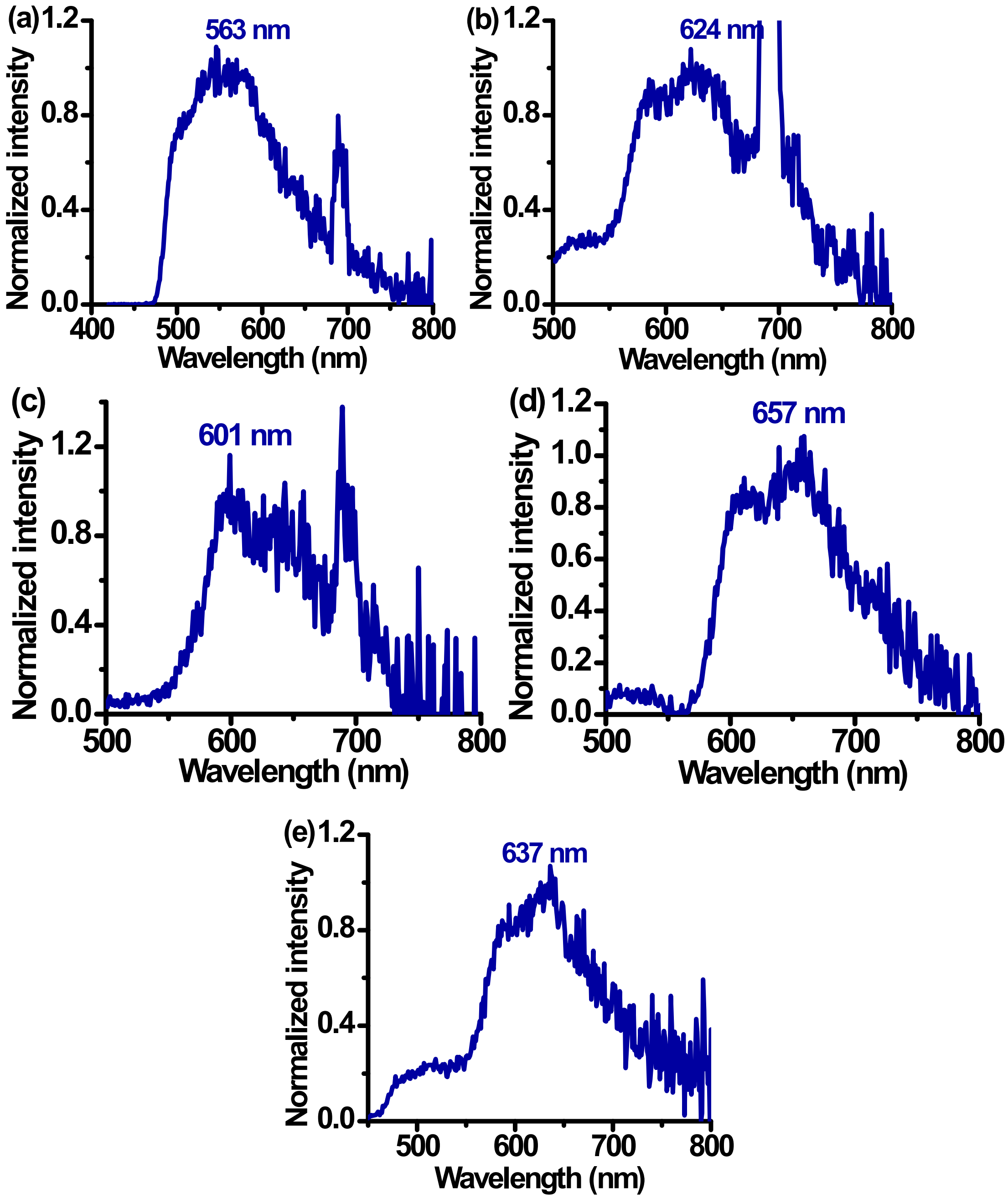

Figure S36. Phosphorescence spectra of OBN4-Ph (a), OBN8-F (b), OBN8-C8 (c), OBN8-Ph (d), and OBN10-Ph (e) by distributed in PMMA films (1.0 wt%) tested in 77 K.

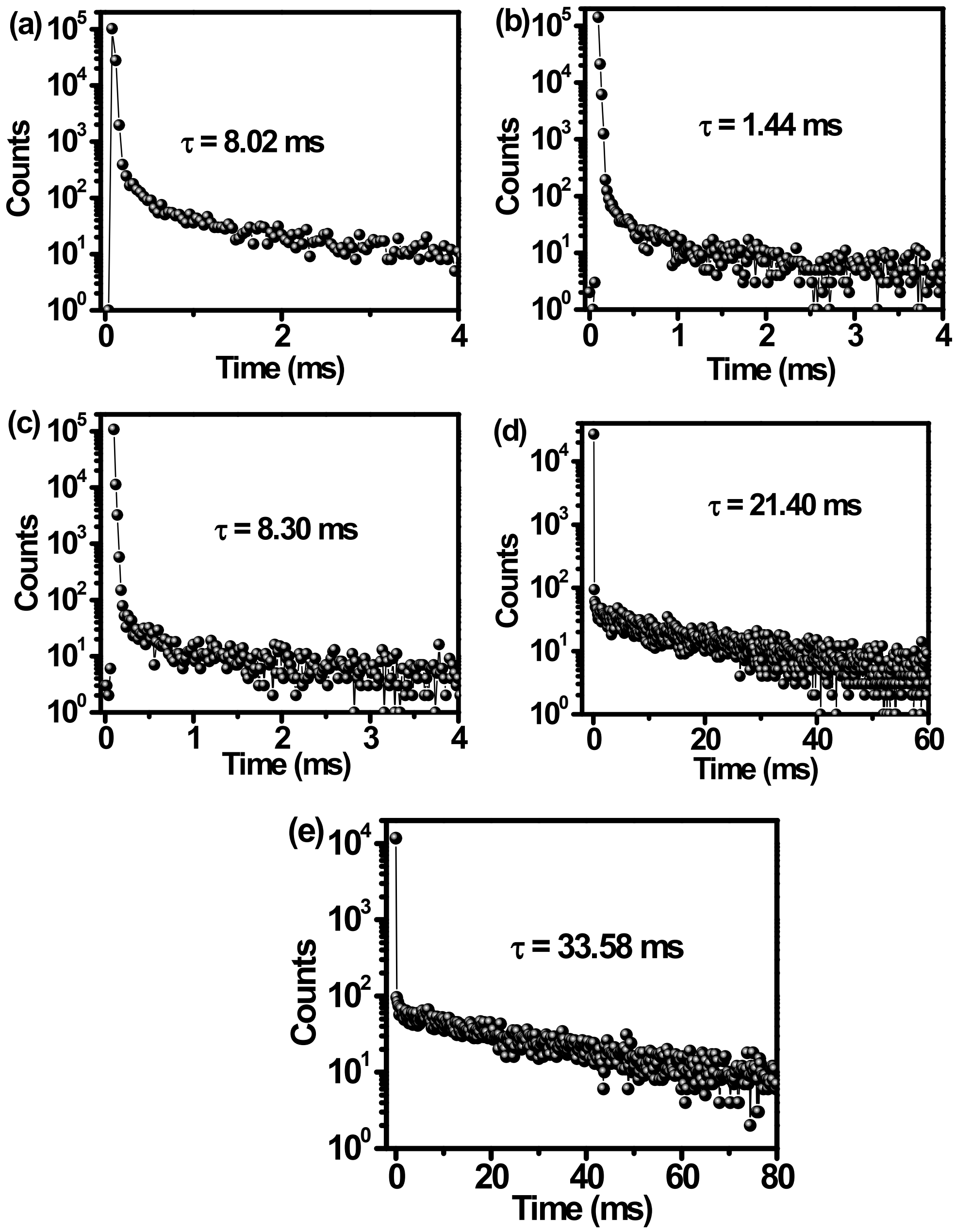

Figure S37. Transient decay phosphorescence spectra of OBN4-Ph (a), OBN8-F (b), OBN8-C8 (c), and OBN8-Ph (d), and OBN10-Ph (e).

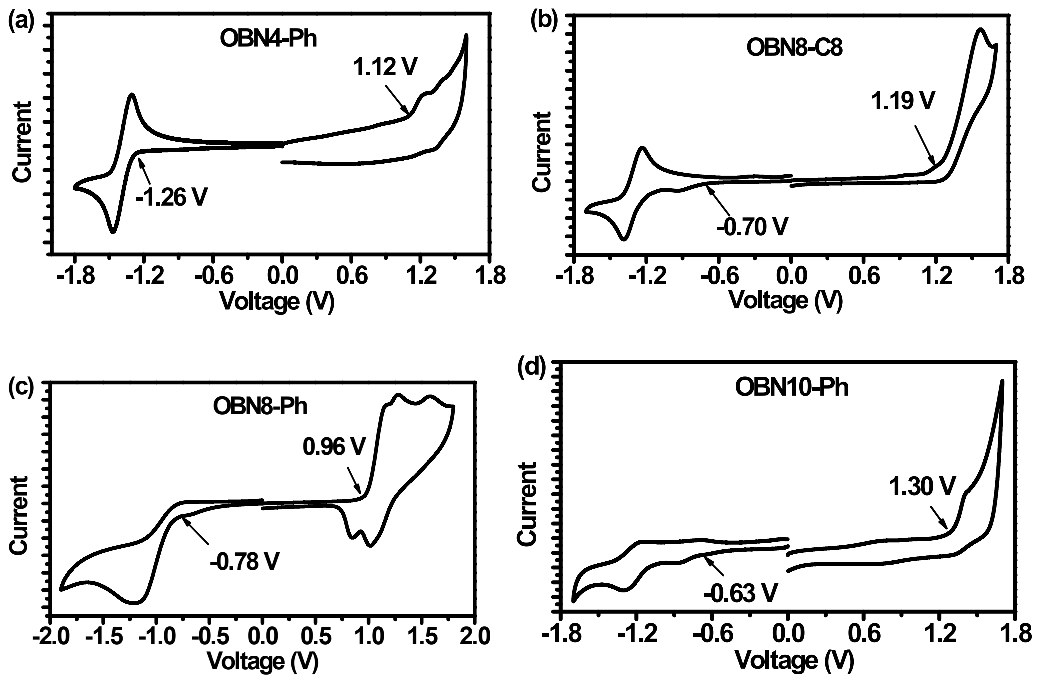

Figure S38. CV curves of OBN4-Ph (a), OBN8-C8 (b), OBN8-Ph (c), and OBN10-Ph (d).

7. Synthesis, characterization, and optical waveguide tests of crystals

The solvent diffusion strategy, namely, reprecipitation method and the solvent volatilization strategy, namely, drop-casting method are selected to grow the crystals.

Preparation of OBN8-Ph micro-crystals. In a glass vial, OBN8-Ph was dissolved in tetrahydrofuran (THF) to prepare a stock solution with a concentration of 0.5 mg/mL, which was treated with ultrasonic waves for 30 minutes. Optical microscope was adopted to confirm the thorough dissolution of OBN8-Ph by observing the clear solution without any undissolved particles. Subsequently, 200 μL of the clear stock solution was quickly injected into 1 mL of *n*-hexane with a pipette gun and the sealed mixture was left at room temperature for 24 hours to obtain the OBN8-Ph single-crystal micro rods.

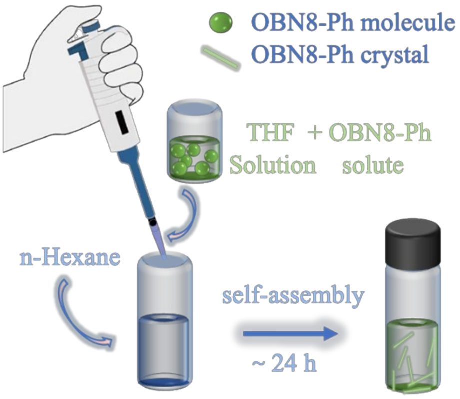

Figure S39. Schematic illustration of preparation processes of OBN8-Ph microrod using the reprecipitation method.

Preparation of OBN10-Ph micro-crystals. In a glass vial, OBN10-Ph was dissolved in THF to formulate a stock solution with a concentration of 0.5 mg/mL, which was treated with ultrasonic waves for 30 minutes. Optical microscope was adopted to confirm the thorough dissolution of OBN8-Ph by observing the clear solution without any undissolved particles. Hexane (2 mL) was added to a clear glass bottle, which was sealed and placed into a water bath preheated to 55°C. The stock solution was heated together in the water bath for 10 minutes. Subsequently, 200 μL of stock solution was quickly injected into hexane using a pipette gun. Then, the water bath temperature was maintained at 55°C for 10 minutes. After that, the heating source of the hydrothermal boiler was turned off and the water temperature was naturally cooled down to room temperature (about 1 °C/min). Finally, it was left to stand for about 24 hours to obtain the sheeted single crystals.

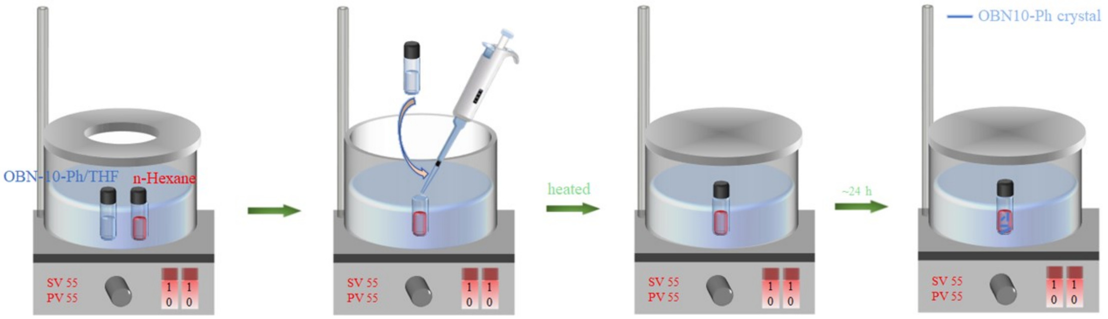

Figure S40. Schematic illustration of preparation processes of OBN10-Ph micro-cuboid using the reprecipitation method.

Preparation of OBN8-C8 micro-crystals. OBN8-C8 was dissolved in ethyl acetate to form a stock solution with a concentration of 1 mg/mL, which was treated with ultrasonic waves for 30 minutes. Optical microscope was adopted to confirm the thorough dissolution of OBN8-C8 by observing the clear solution without any undissolved particles. Then 100 μL of stock solution was transferred using a pipette gun onto an 18 mm × 18 mm carrier slide, which was placed in a Petri dish. Then, 50 mL of the poor solvent n-hexane was added dropwise into the Petri dish. Finally, the petri dish was sealed with a sealing film and waited for the end of the natural evaporation of the solvent to obtain the spindle-shaped single crystals.

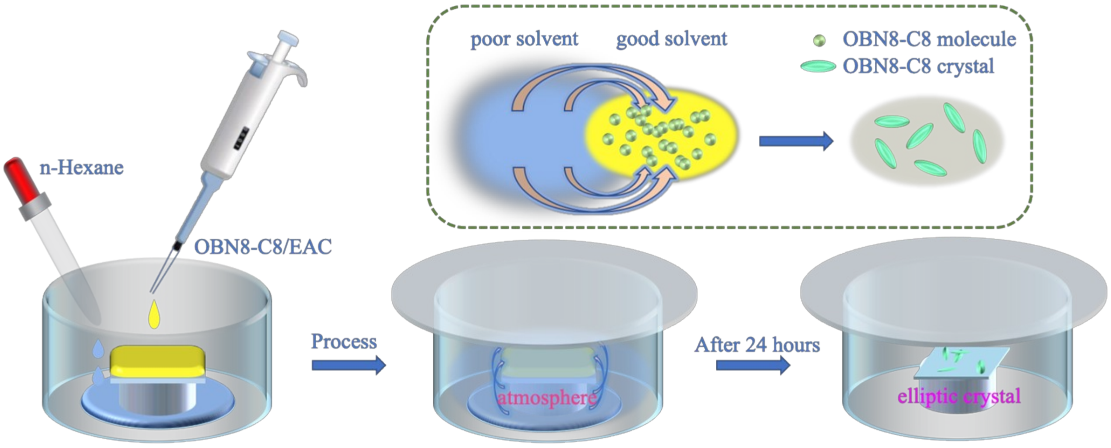

Figure S41. Schematic illustration of preparation processes of OBN8-C8 micro-crystals using the drop-casting method.

Characterization and optical waveguide measurements. Optical microscope images were measured using an Olympus BX53M. The surface morphology and thickness of the OBN8-Ph and OBN10-Ph single crystals were obtained from atomic force microscopy using a Multimode 8HR AFM (BRUKERBRUKER). Transmission electronic microscopy (TEM) and selected area electron diffraction (SAED) of the OBN8-Ph and OBN10-Ph crystals were performed using a JEOL-2100plus (JEOL) and a JEOL-F200 (JEOL), respectively. To obtain the coefficient of optical loss, the crystals were locally excited with a 375 nm laser focused to the diffraction limit. The

excitation laser was filtered through a 375 nm notch filter. The light from the crystals was then guided to the end and recorded by a thermoelectrically cooled CCD (Princeton Instruments, PIX-256 E). PL microscopy images were captured using an inverted microscope (Olympus, BX 43).

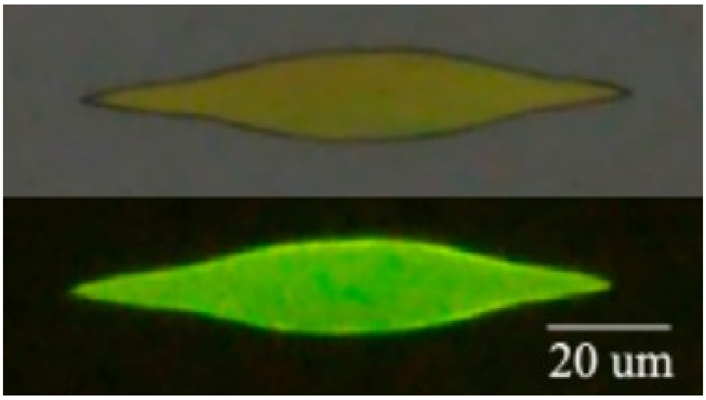

Figure S42. Fluorescence microscopy images of OBN8-C8 microcrystals.

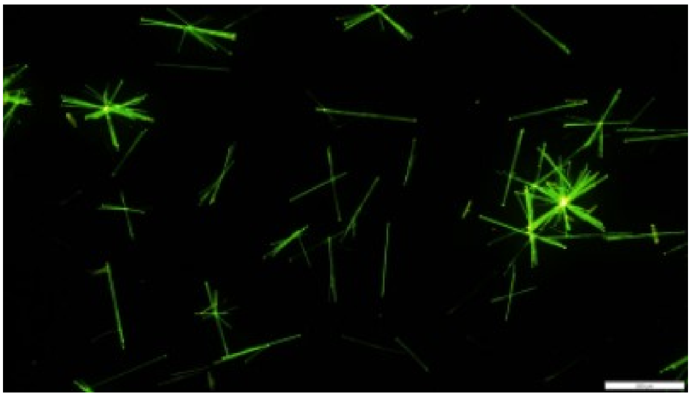

Figure S43. Fluorescence microscopy images of OBN8-Ph microcrystals. Scale bar:100 μm.

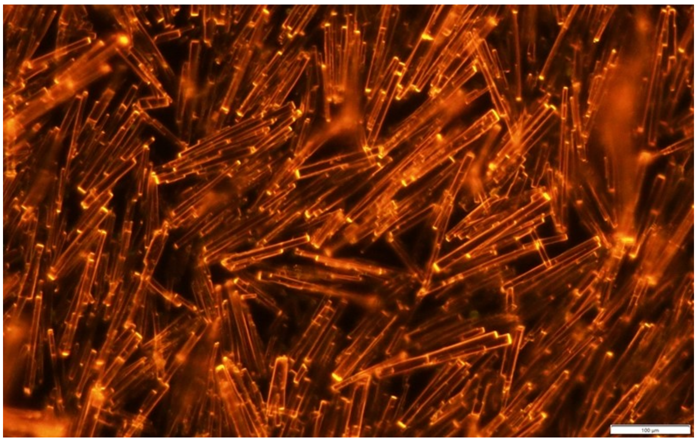

Figure S44. Fluorescence microscopy images of OBN10-Ph microcrystals. Scale bar:100 μm.

Table S12. Some reported luminescent molecules and their optical waveguide performance

Molecular structure	Morphology	*R* (dB/μm)	Ref
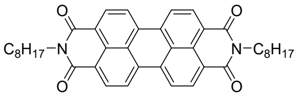	microrod	0.13	Adv. Mater. 2010, 22, 3361
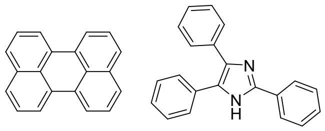	1D uniformly doped microtube	0.097	Adv. Mater. 2009, 21, 4153
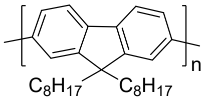	nanowire	0.48	Small, 2007, 3, 1178
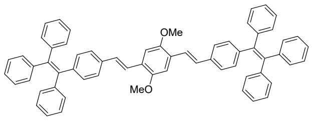	microribbon	0.012	J. Phys. Chem. Lett. 2019, 10, 679
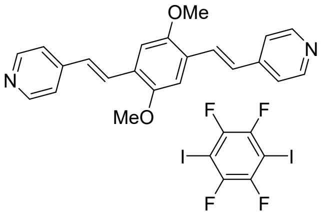	microtube, binary co-crystalline	0.0145	J. Mater. Chem. C, 2018, 6, 9594
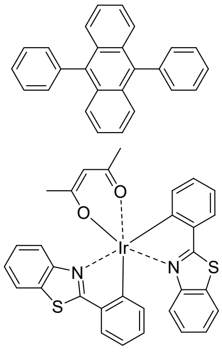	1D uniformly doped microwire	0.13	Adv. Mater. 2011, 23, 1380
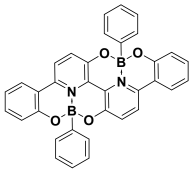	microrod	0.0139	This work
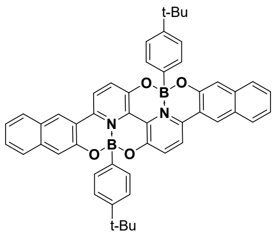	microsheet	0.00706	This work

8. NMR spectra and HRMS

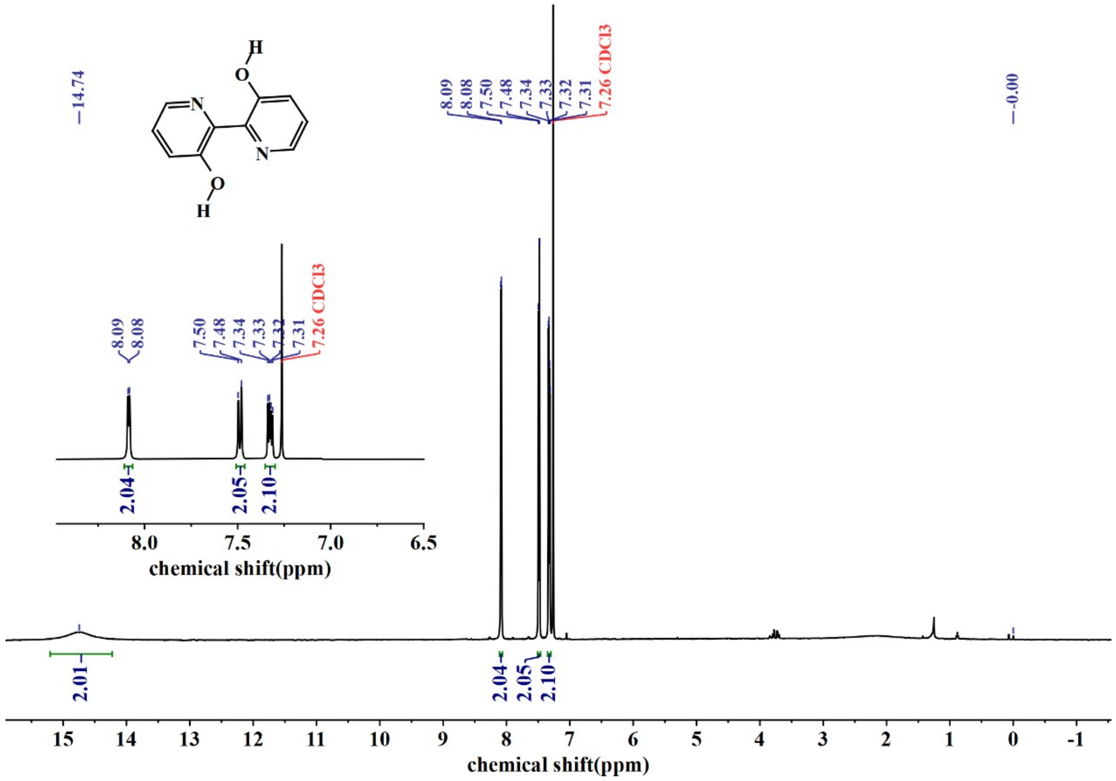

Figure S45. ^{1}HNMR spectra of BP.

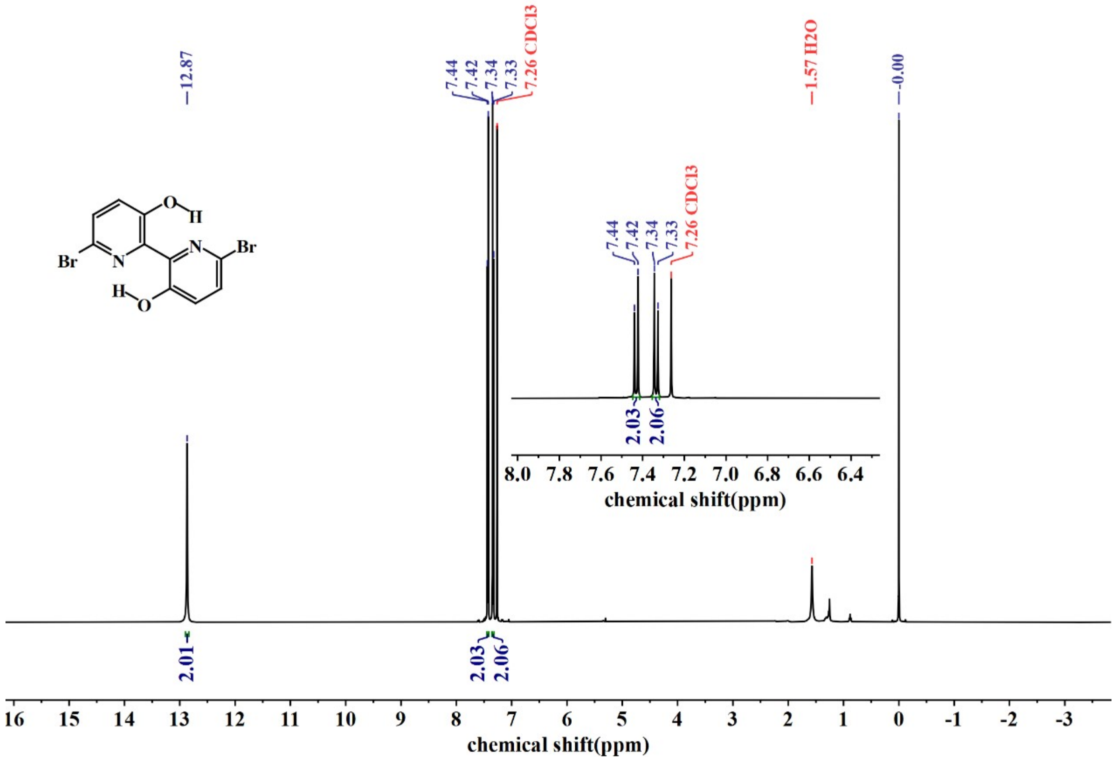

Figure S46. ^{1}HNMR spectra of BP-Br

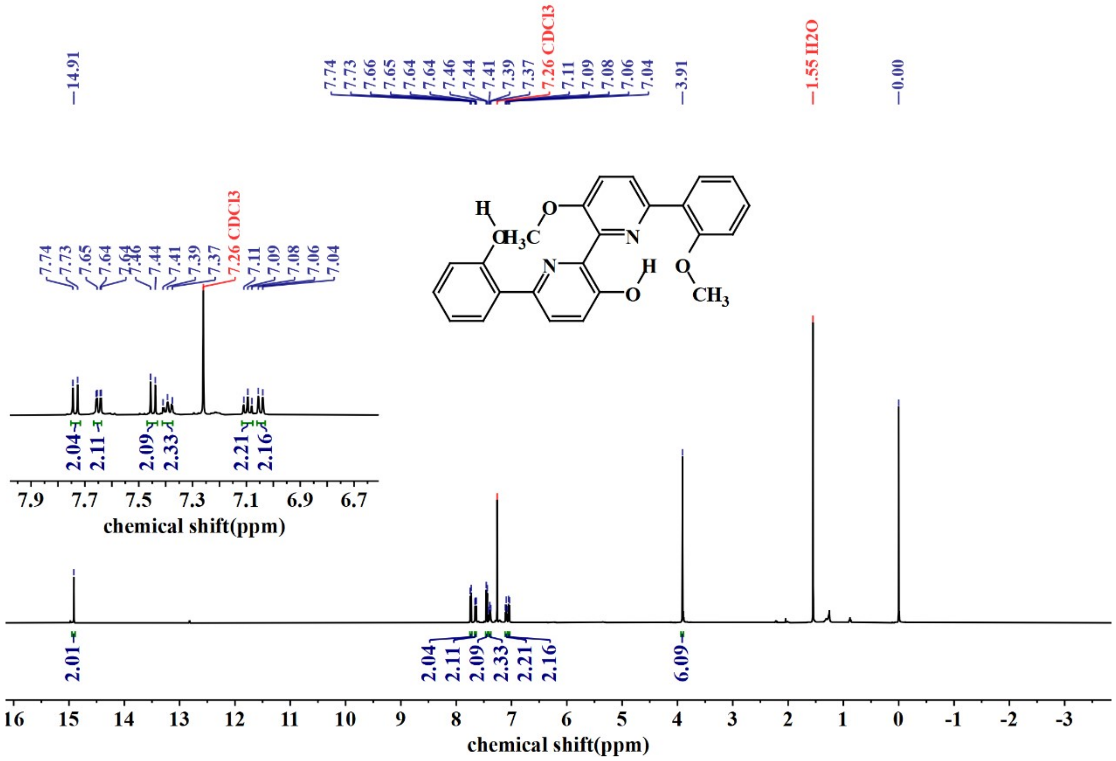

Figure S47. ^{1}HNMR spectra of BP-Ph

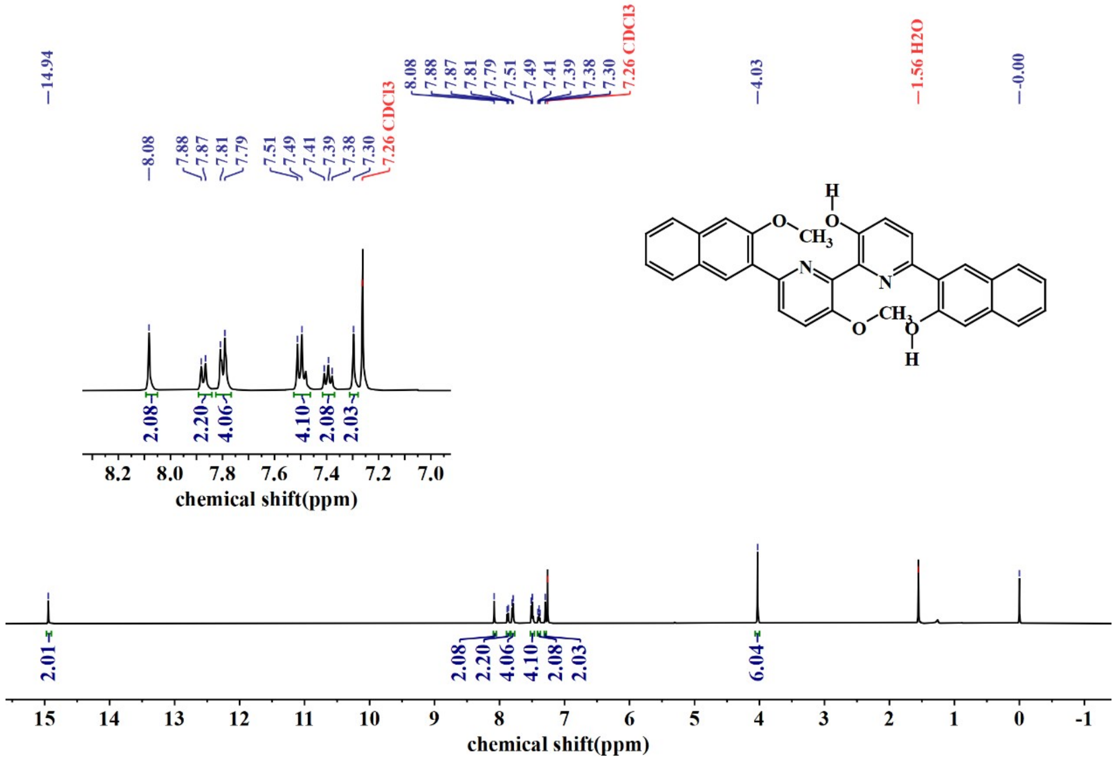

Figure S48. ^{1}HNMR spectra of BN-N.

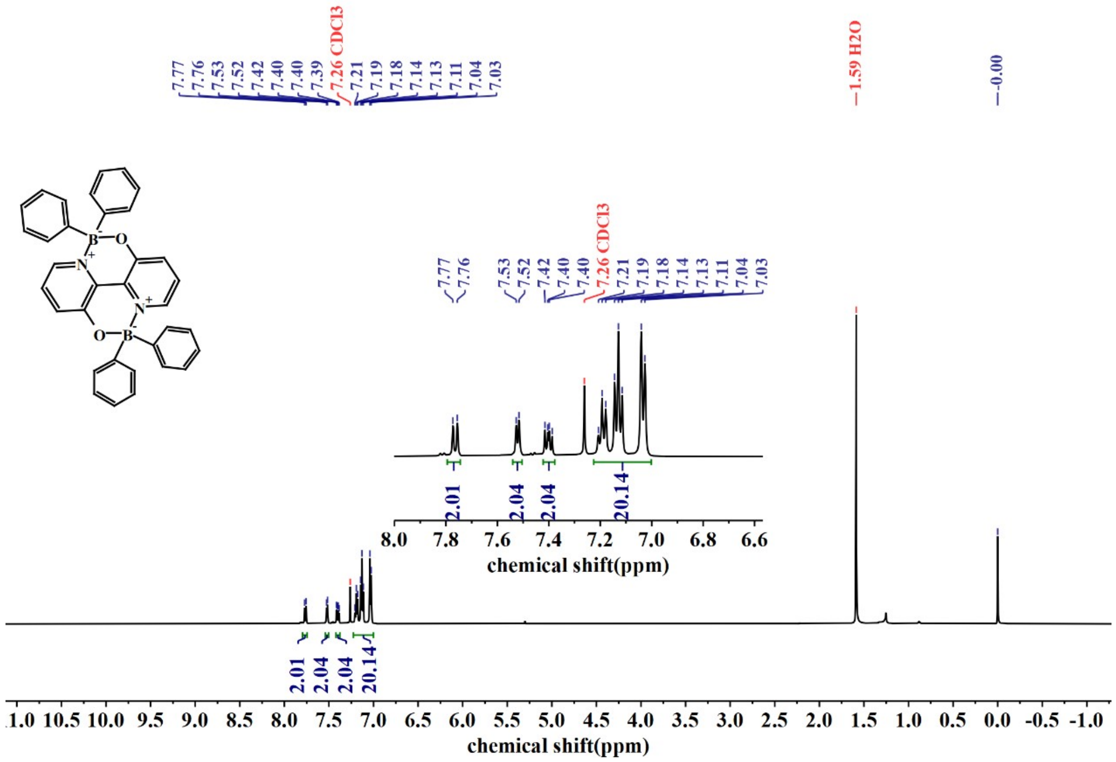

Figure S49. ^{1}HNMR spectra of OBN4-Ph

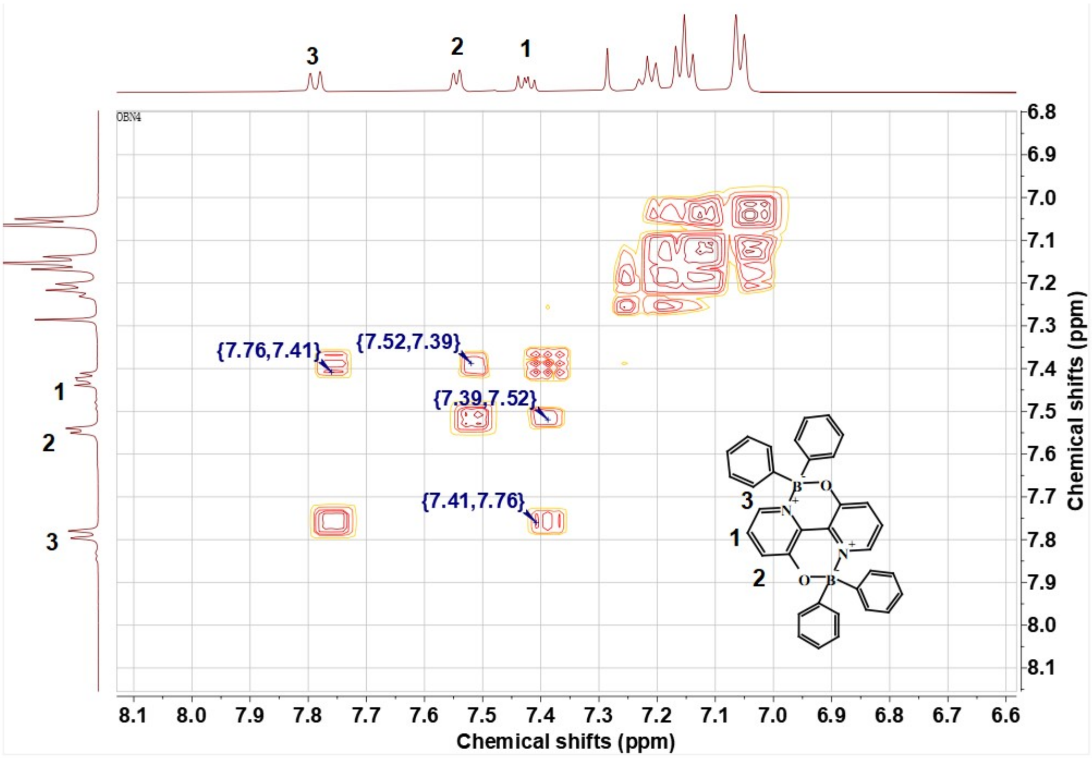

Figure S50. H-H COSY spectra of OBN4-Ph.

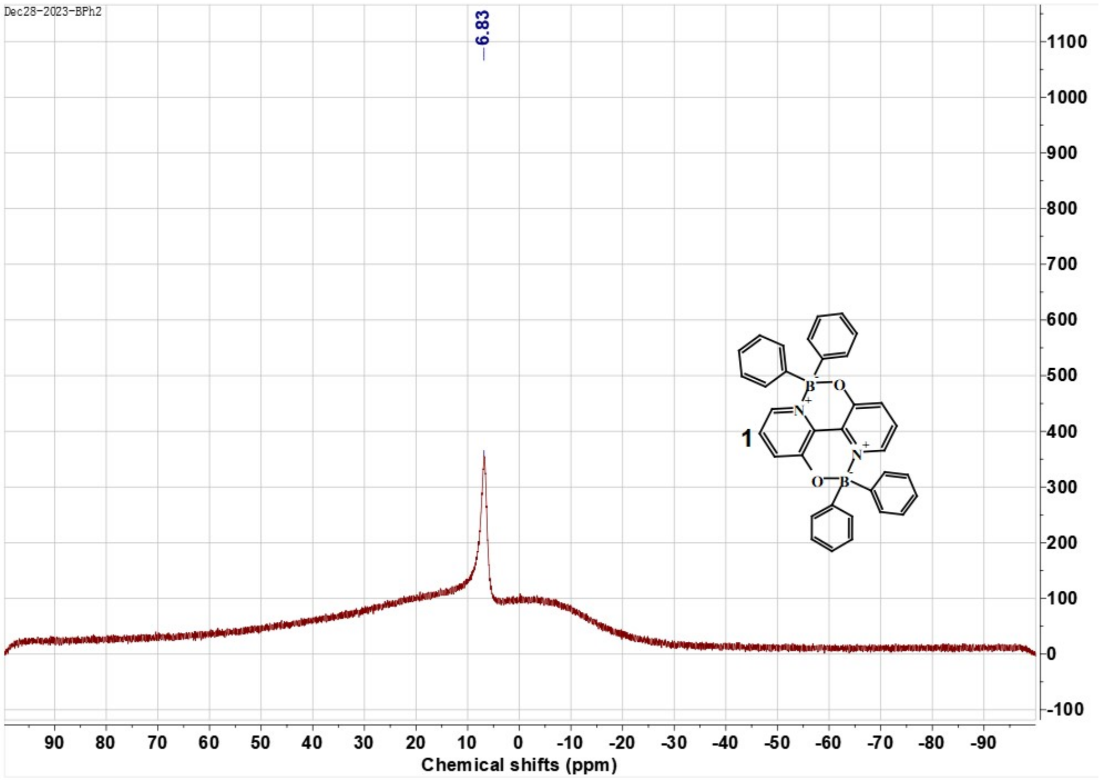

Figure S51. ^{11}BNMR spectra of OBN4-Ph

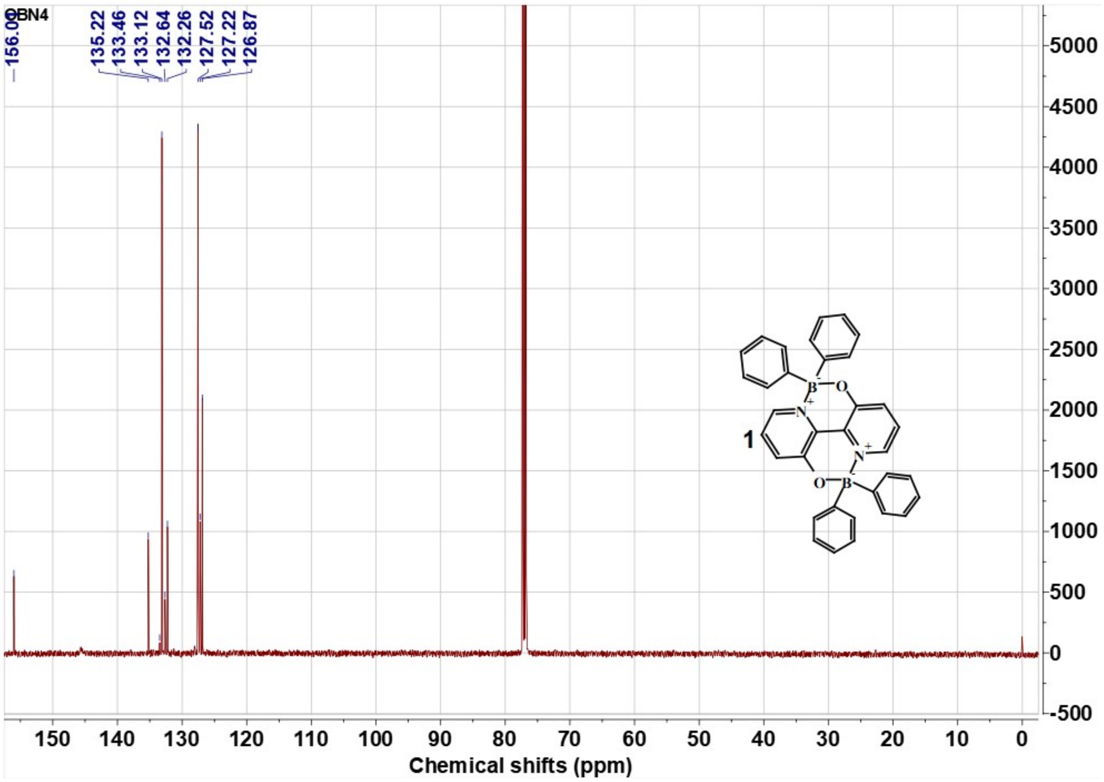

Figure S52. ^{13}CNMR spectra of OBN4-Ph

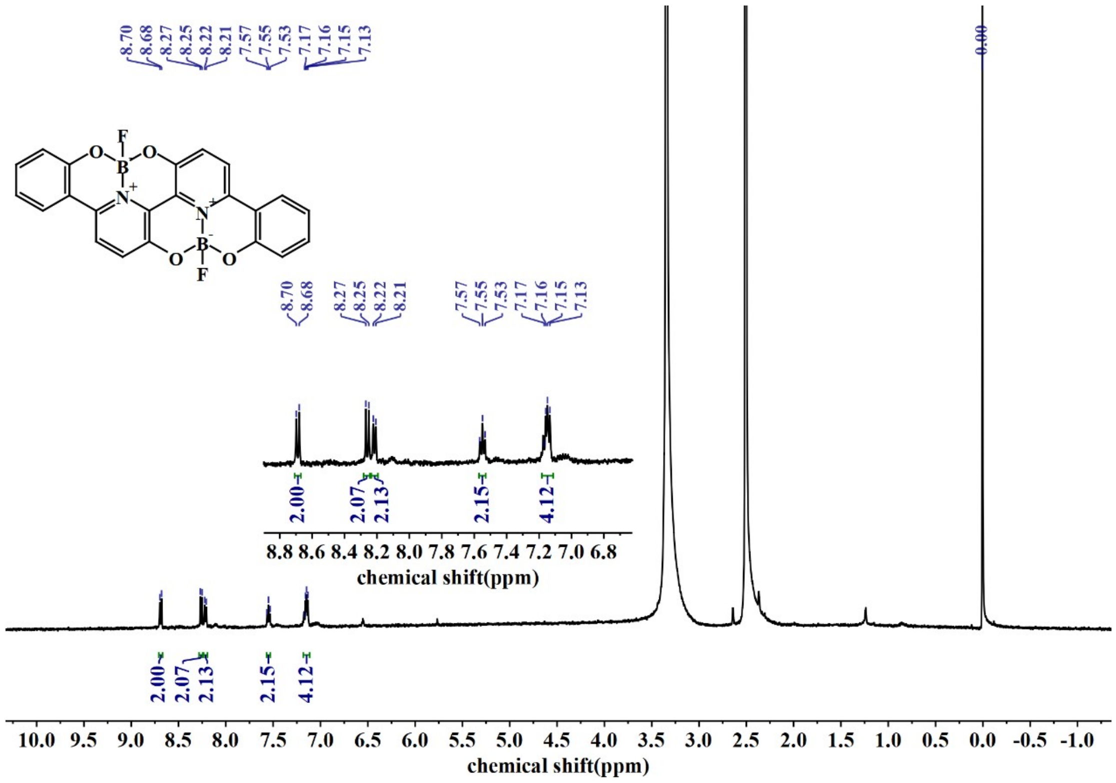

Figure S53. ^{1}HNMR spectra of OBN8-F

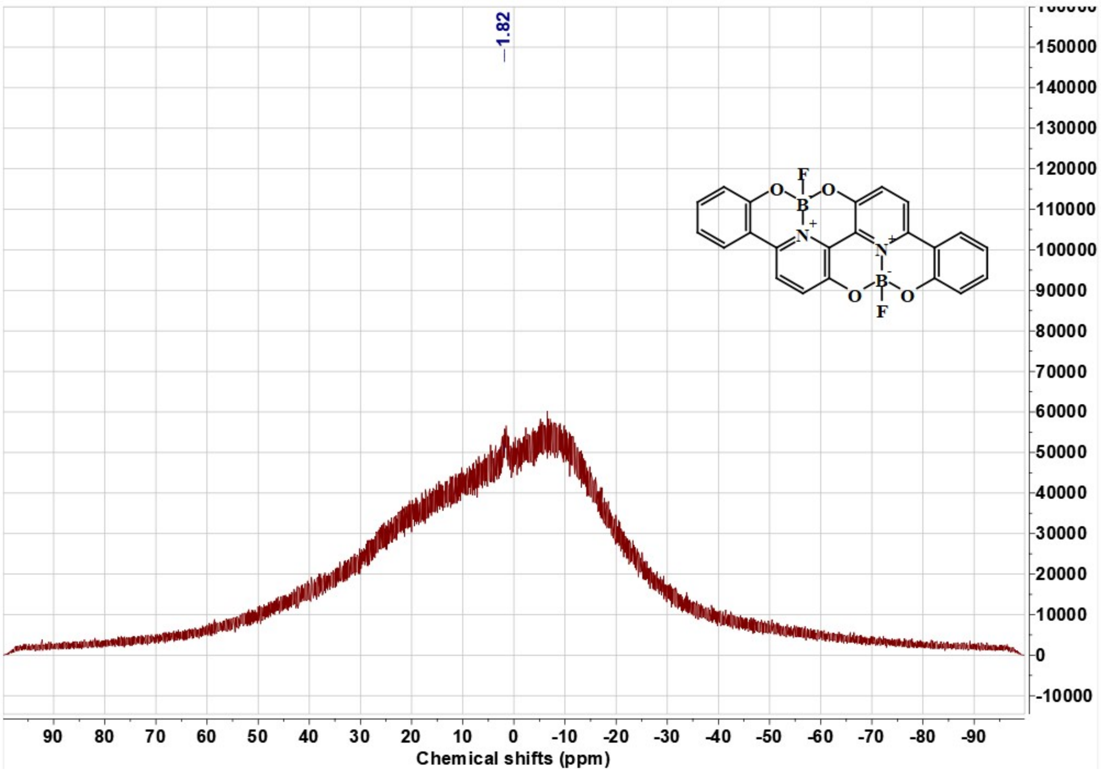

Figure S54. ^{11}BNMR spectra of OBN8-F

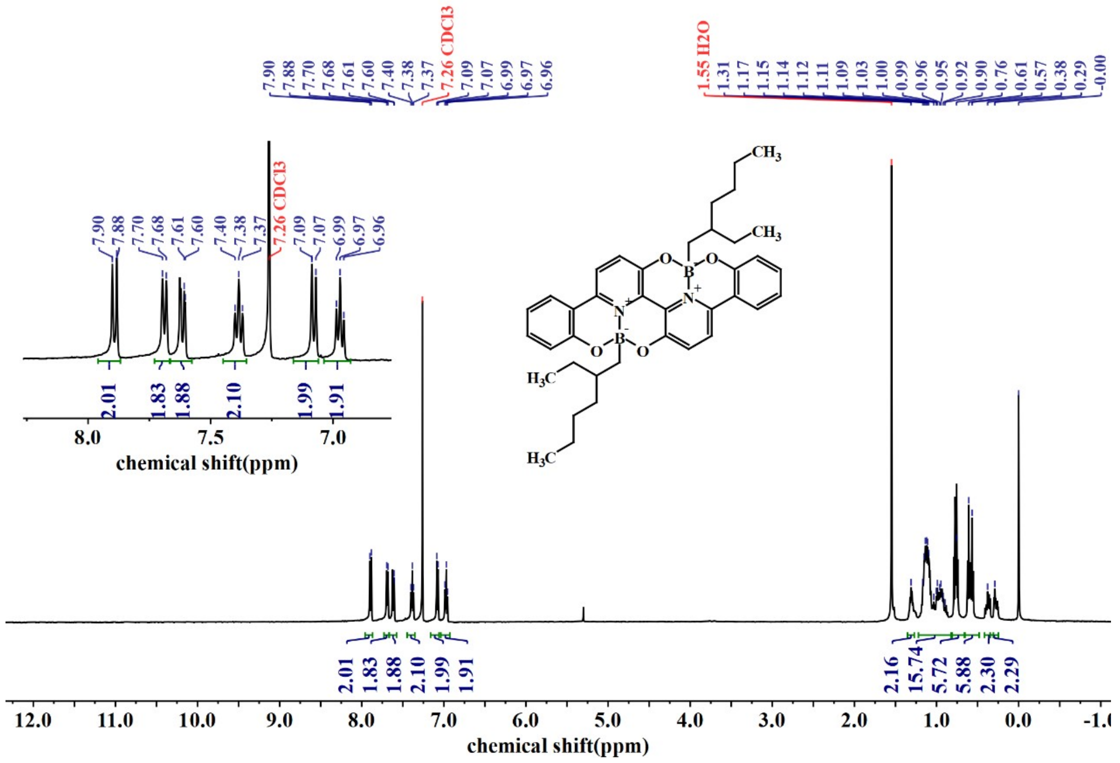

Figure S55. ^{1}HNMR spectra of OBN8-C8.

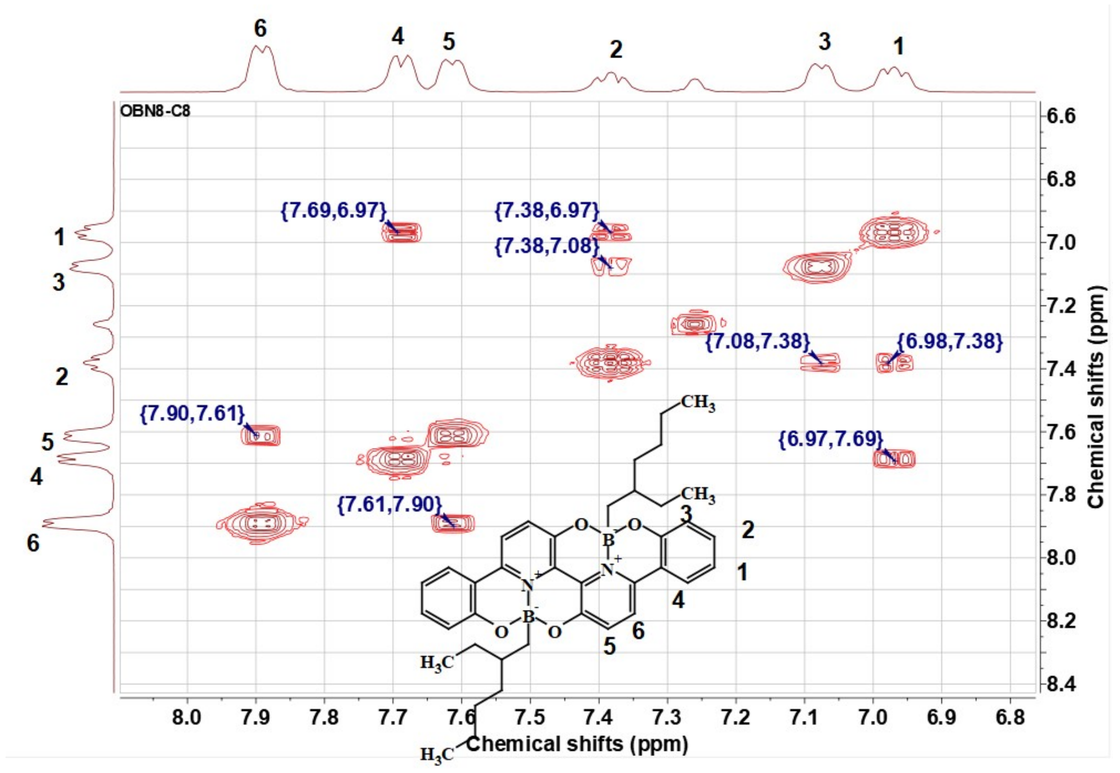

Figure S56. H-H COSY spectra of OBN8-C8

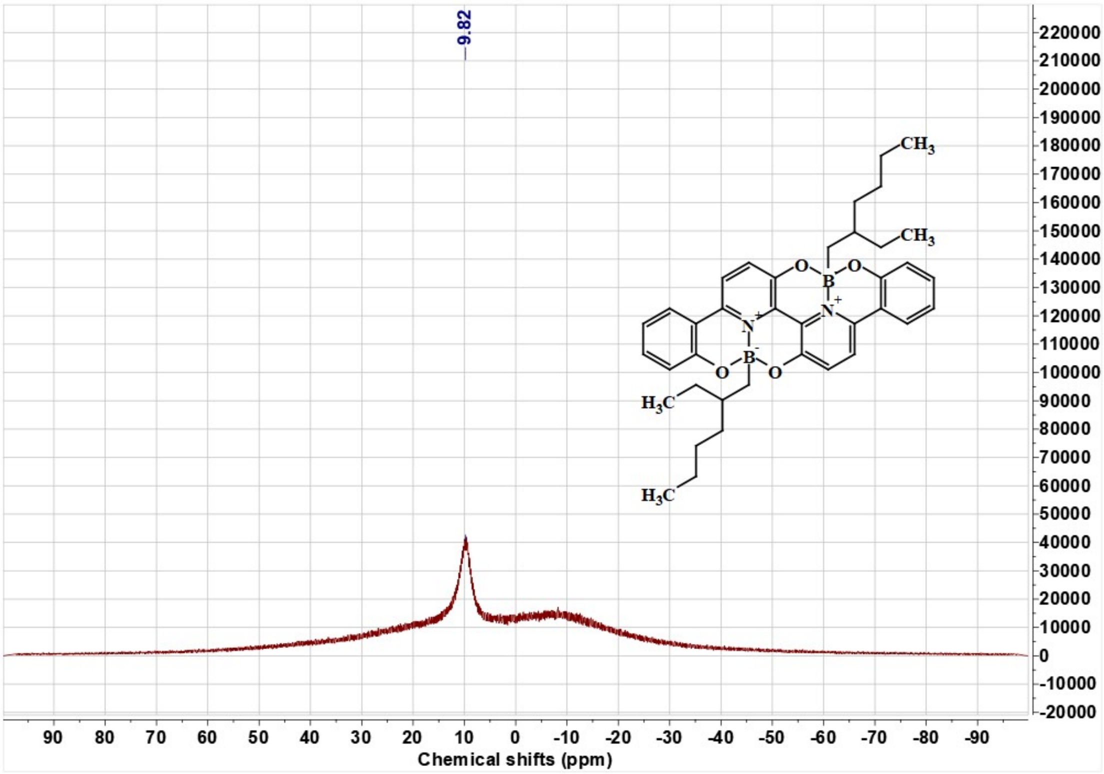

Figure S57. ^{11}BNMR spectra of OBN8-C8

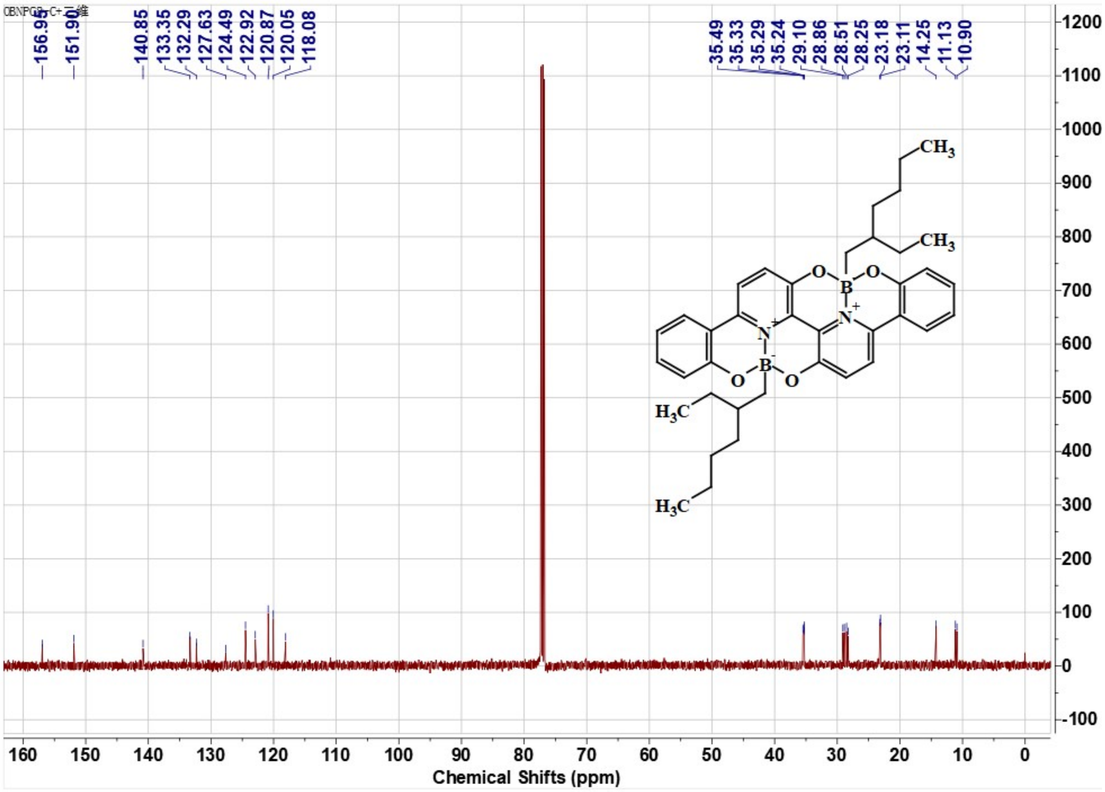

Figure S58. ^{13}CNMR spectra of OBN8-C8

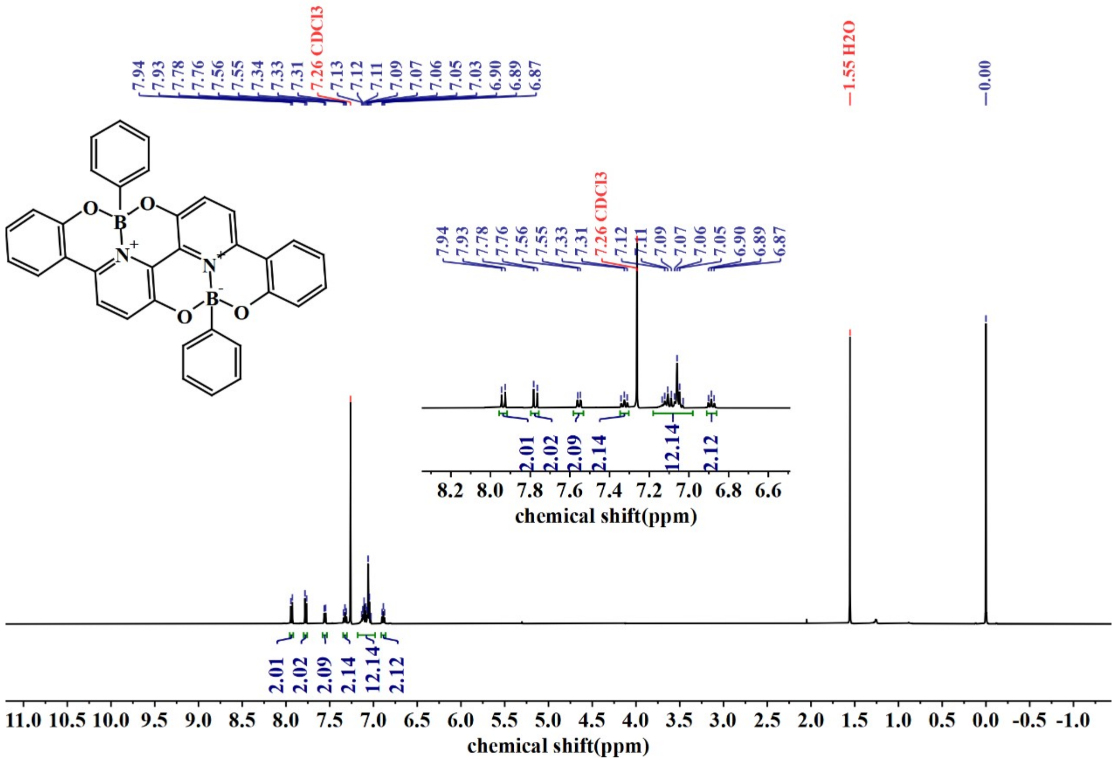

Figure S59. ^{1}HNMR spectra of OBN8-Ph

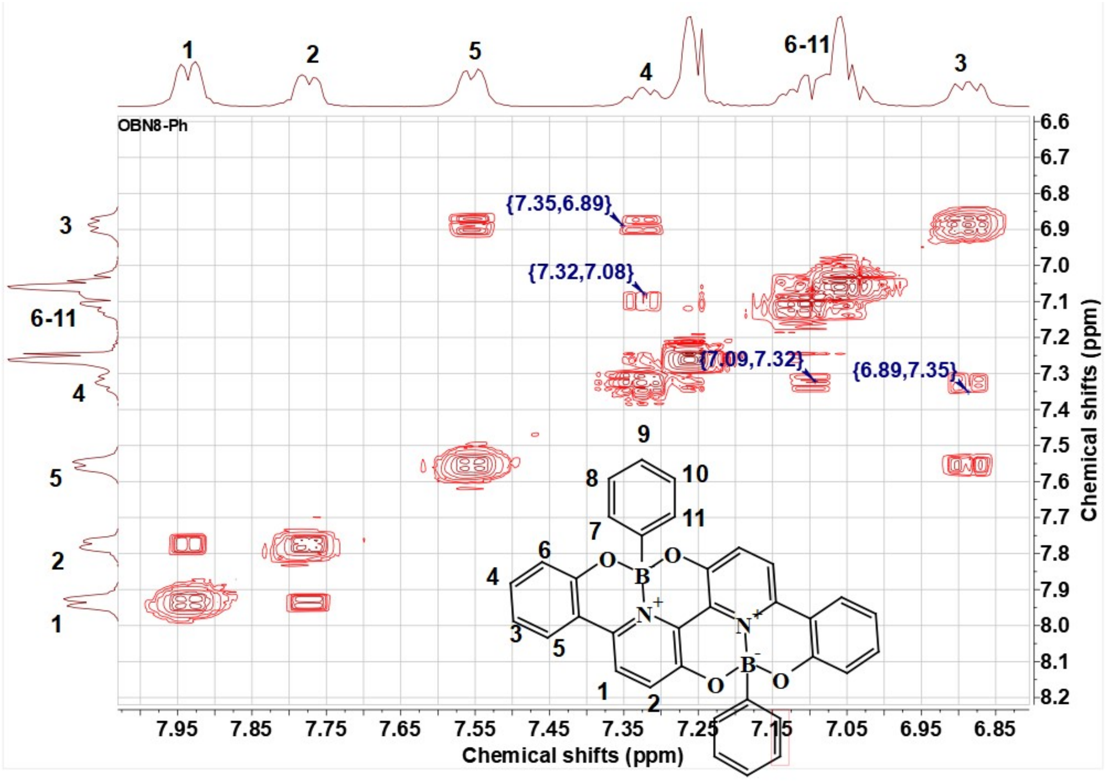

Figure S60. H-H COSY spectra of OBN8-Ph

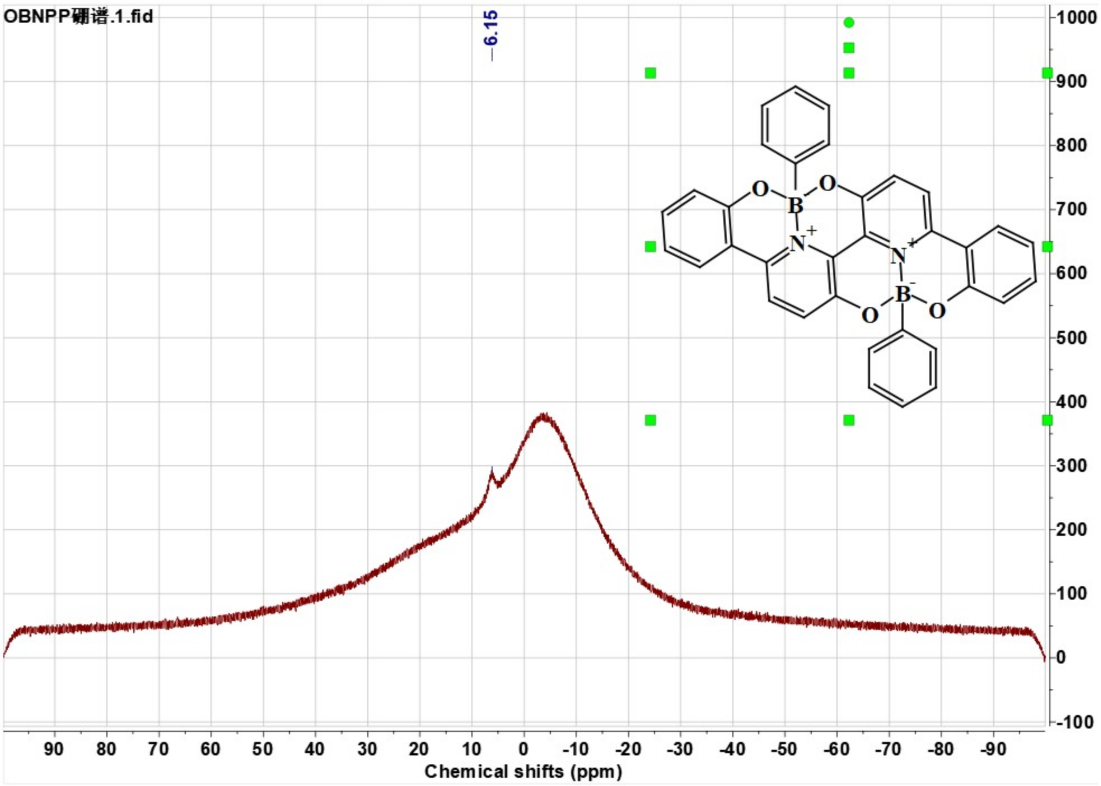

Figure S61. ^{11}BNMR spectra of OBN8-Ph

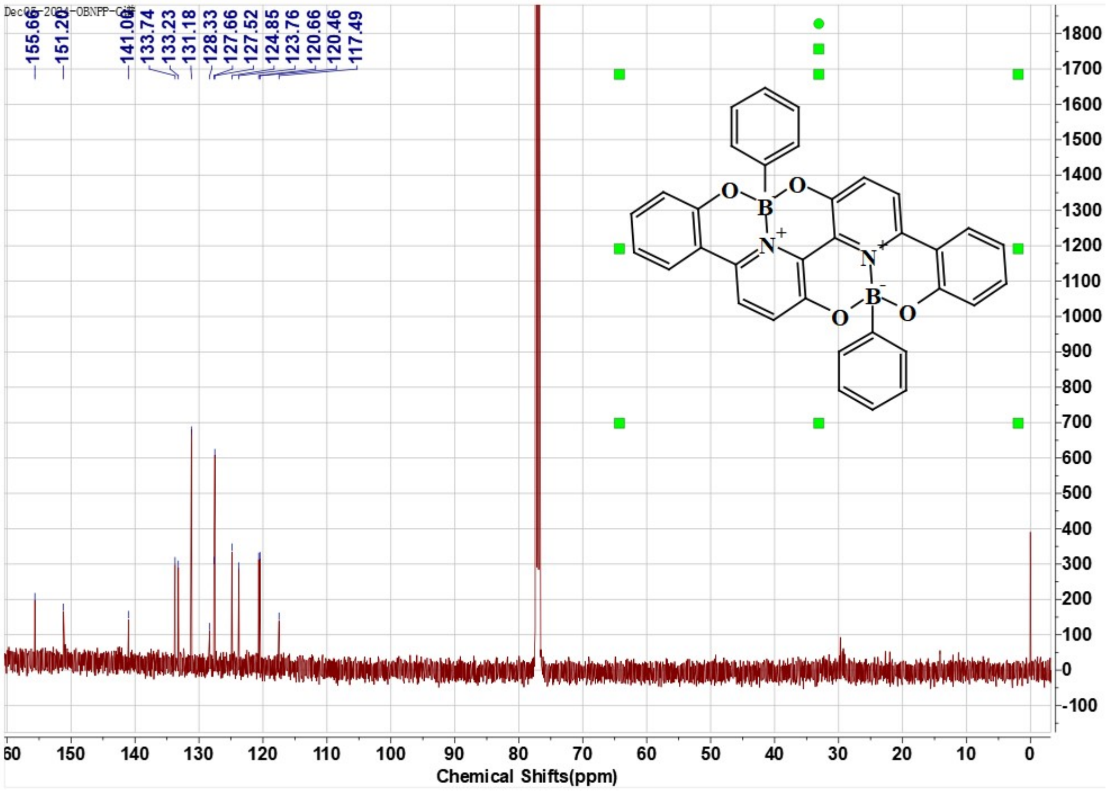

Figure S62. ^{13}CNMR spectra of OBN8-Ph

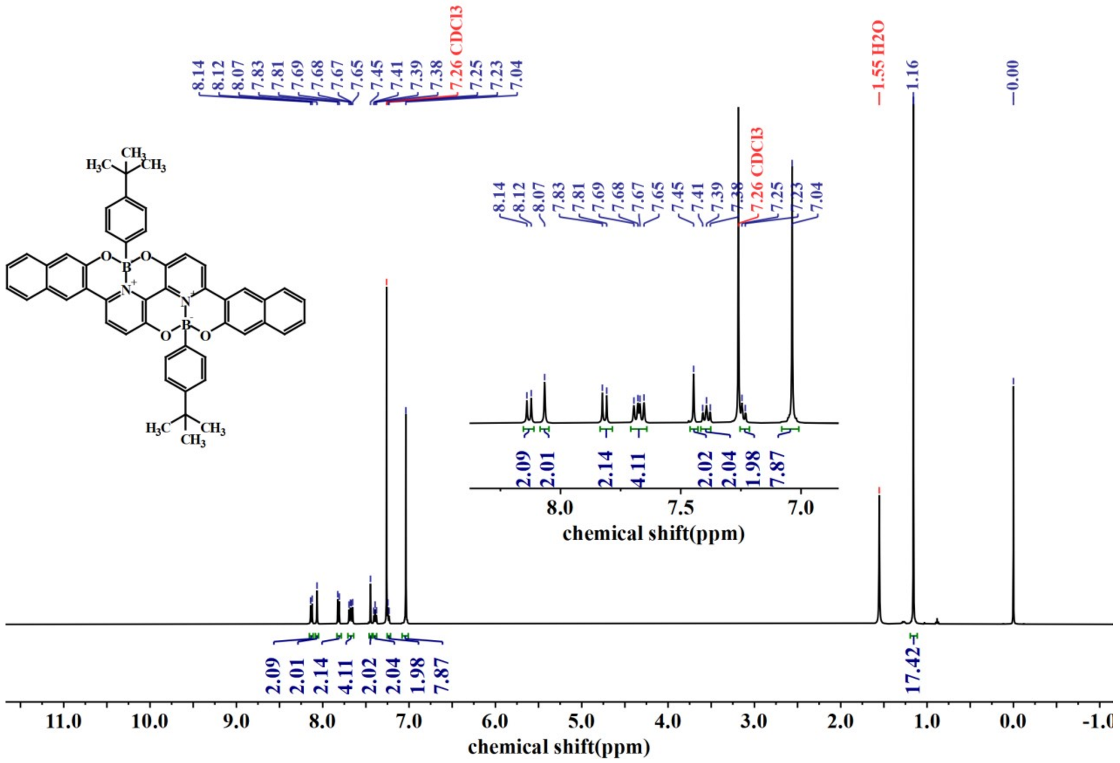

Figure S63. ^{1}HNMR spectra of OBN10-Ph

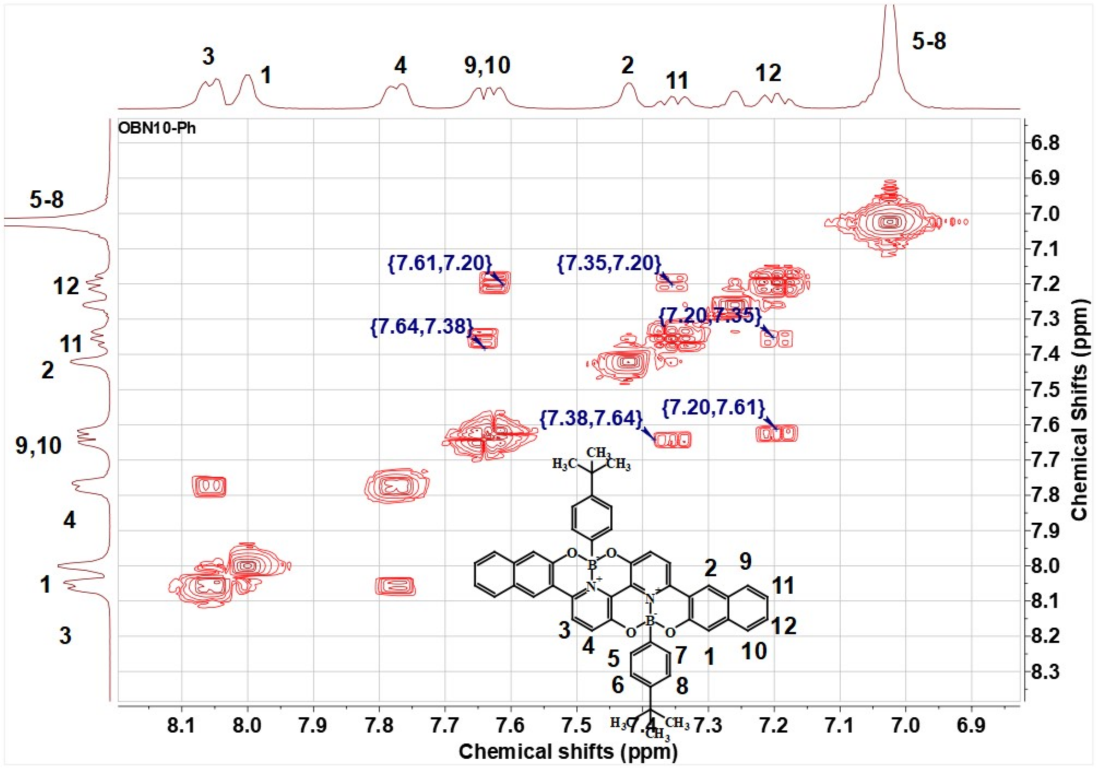

Figure S64. H-H COSY spectra of OBN10-Ph

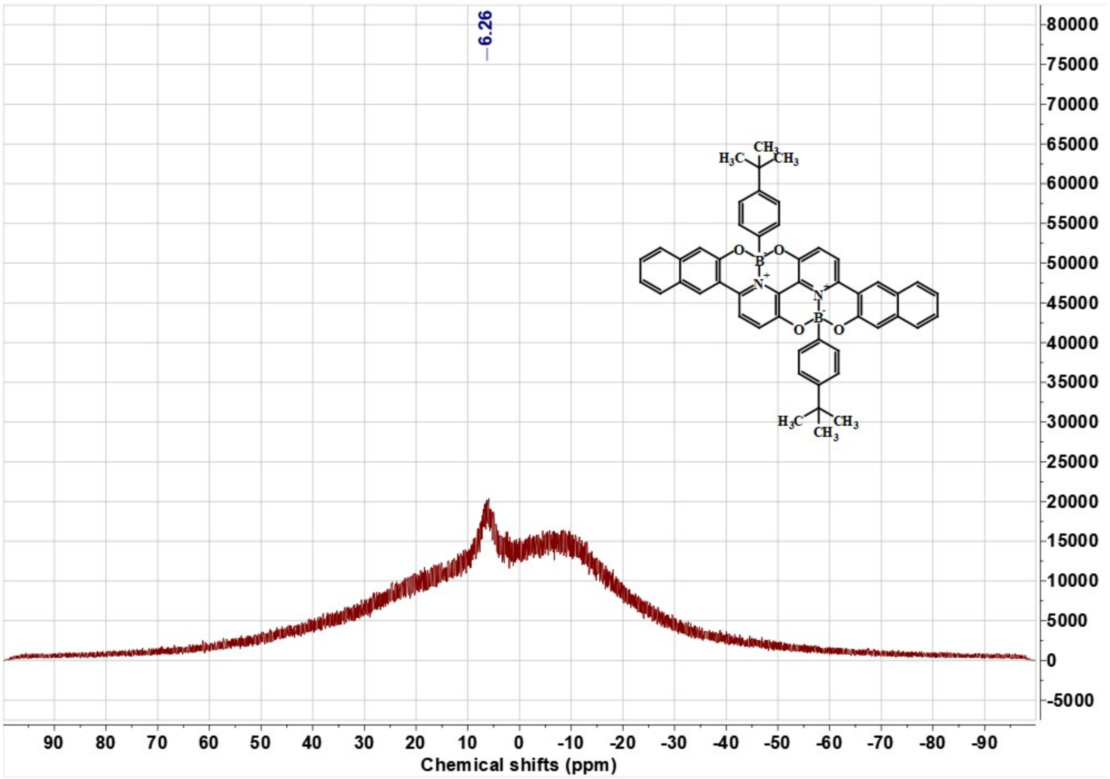

Figure S65. ^{11}BNMR spectra of OBN10-Ph

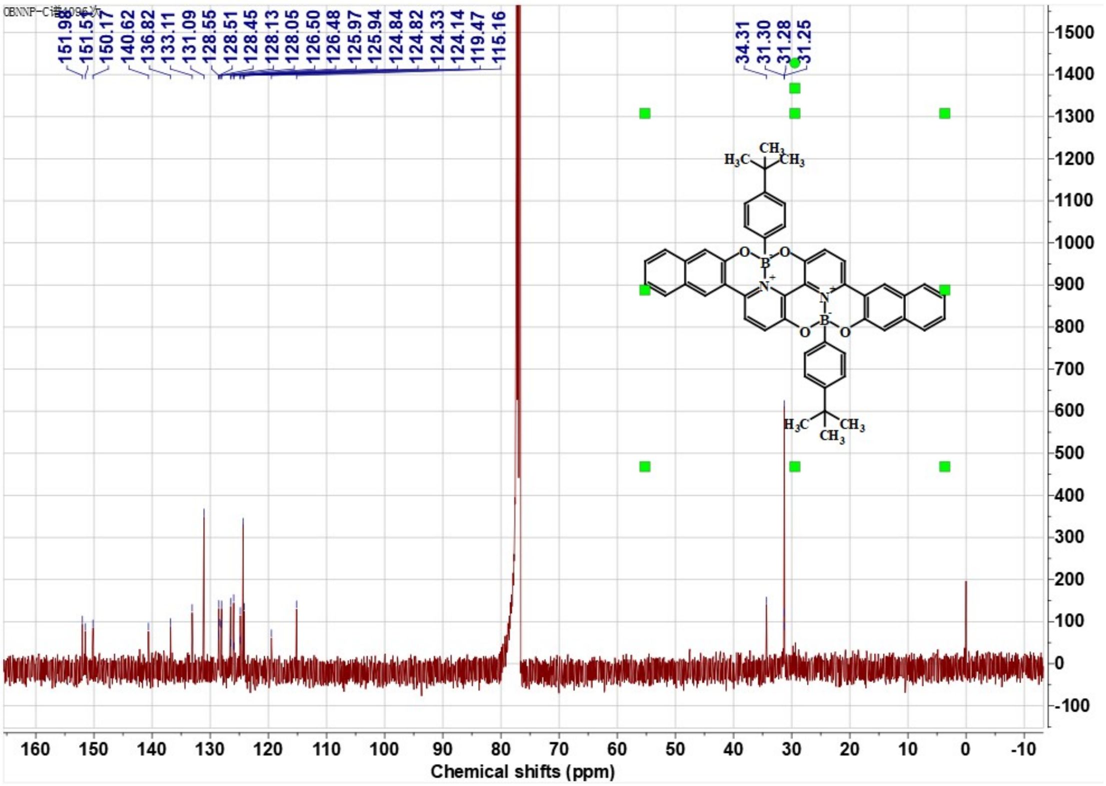

Figure S66. ^{13}CNMR spectra of OBN10-Ph

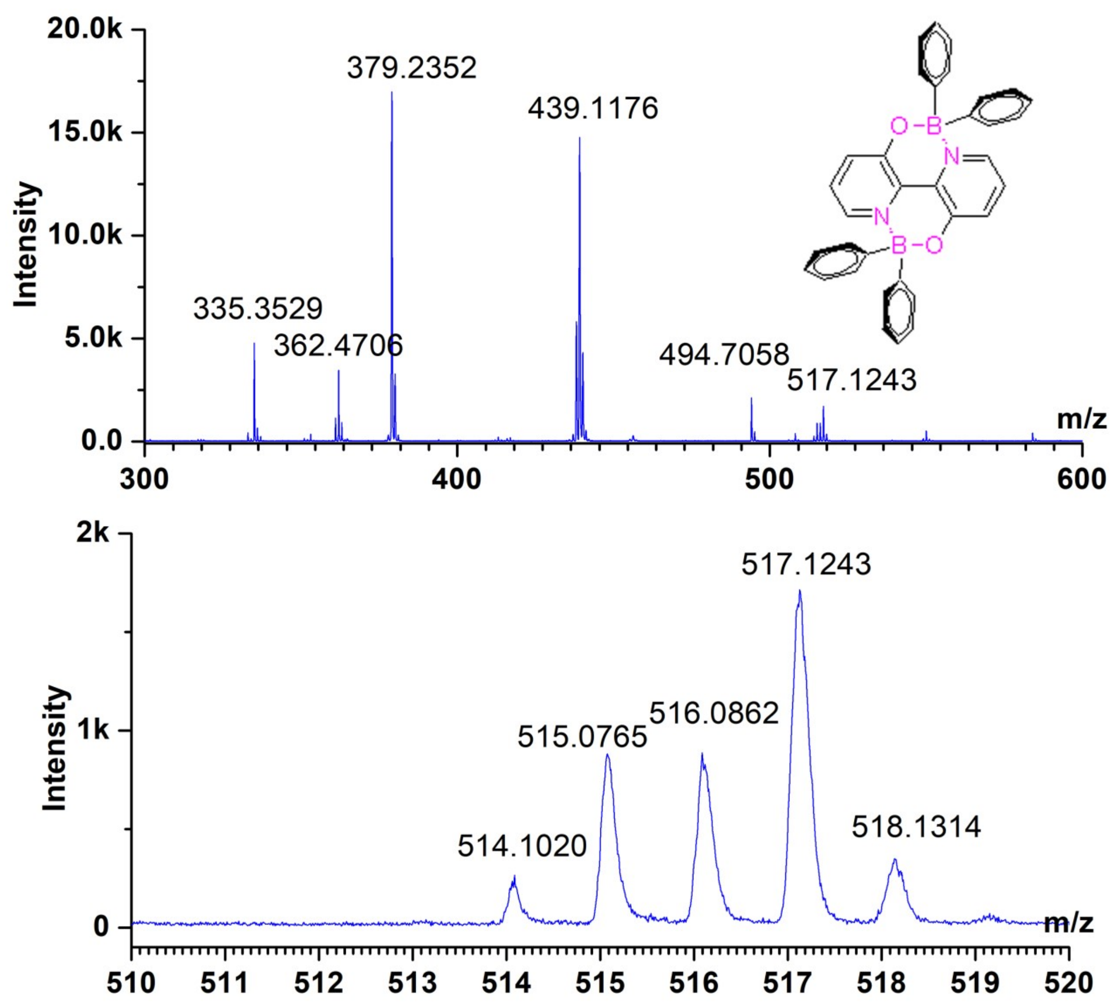

Figure S67. MALDI-TOF MS of OBN4-Ph

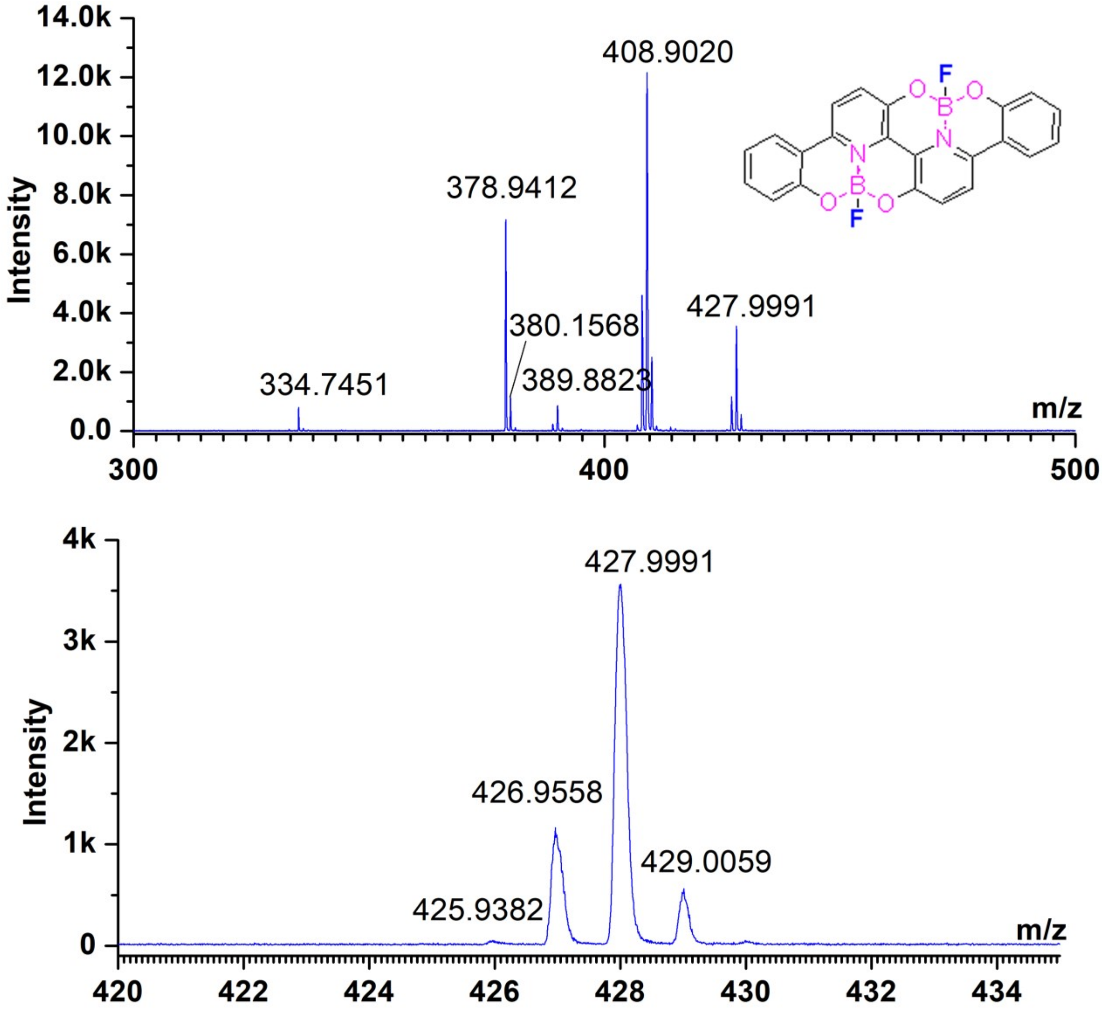

Figure S68. MALDI-TOF MS of OBN8-F

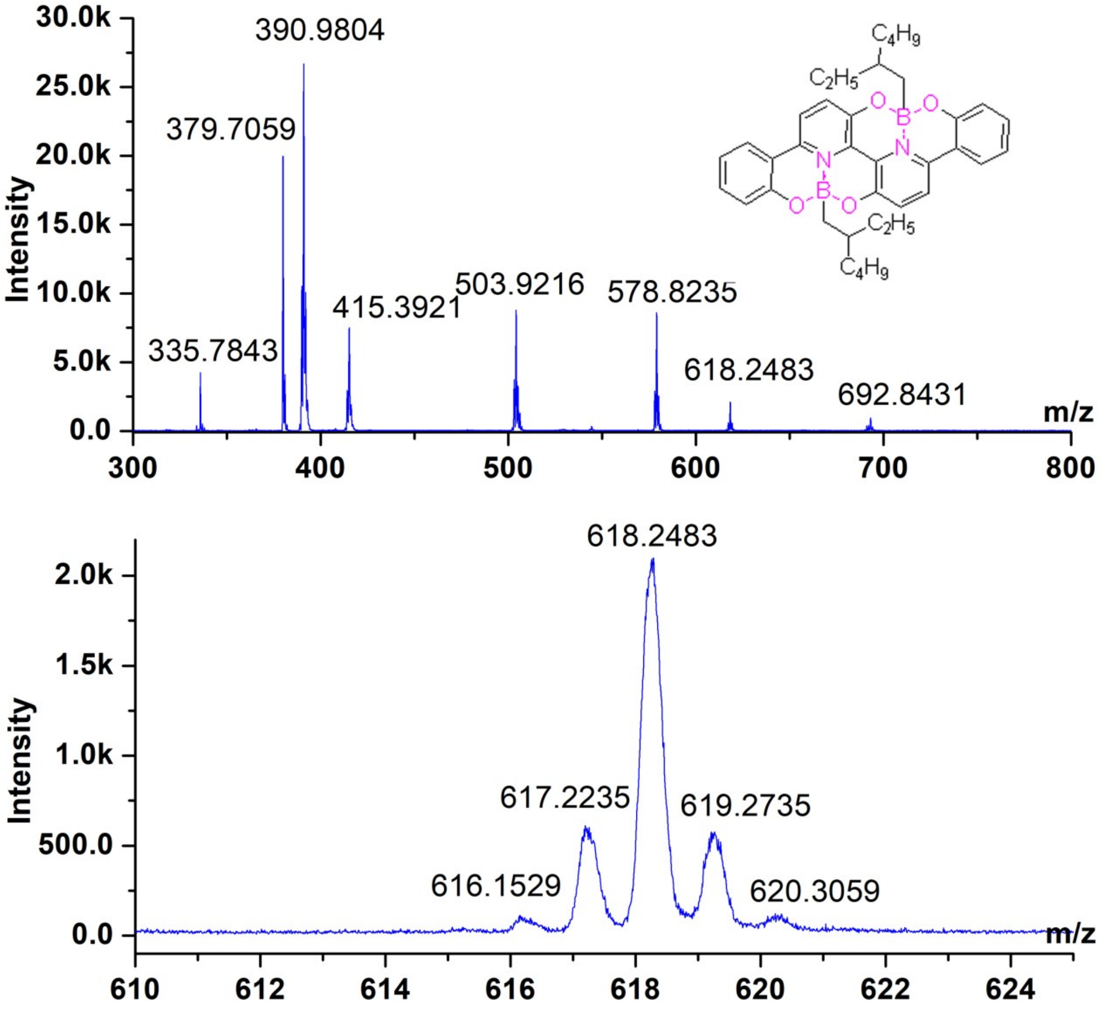

Figure S69. MALDI-TOF MS of OBN8-C8

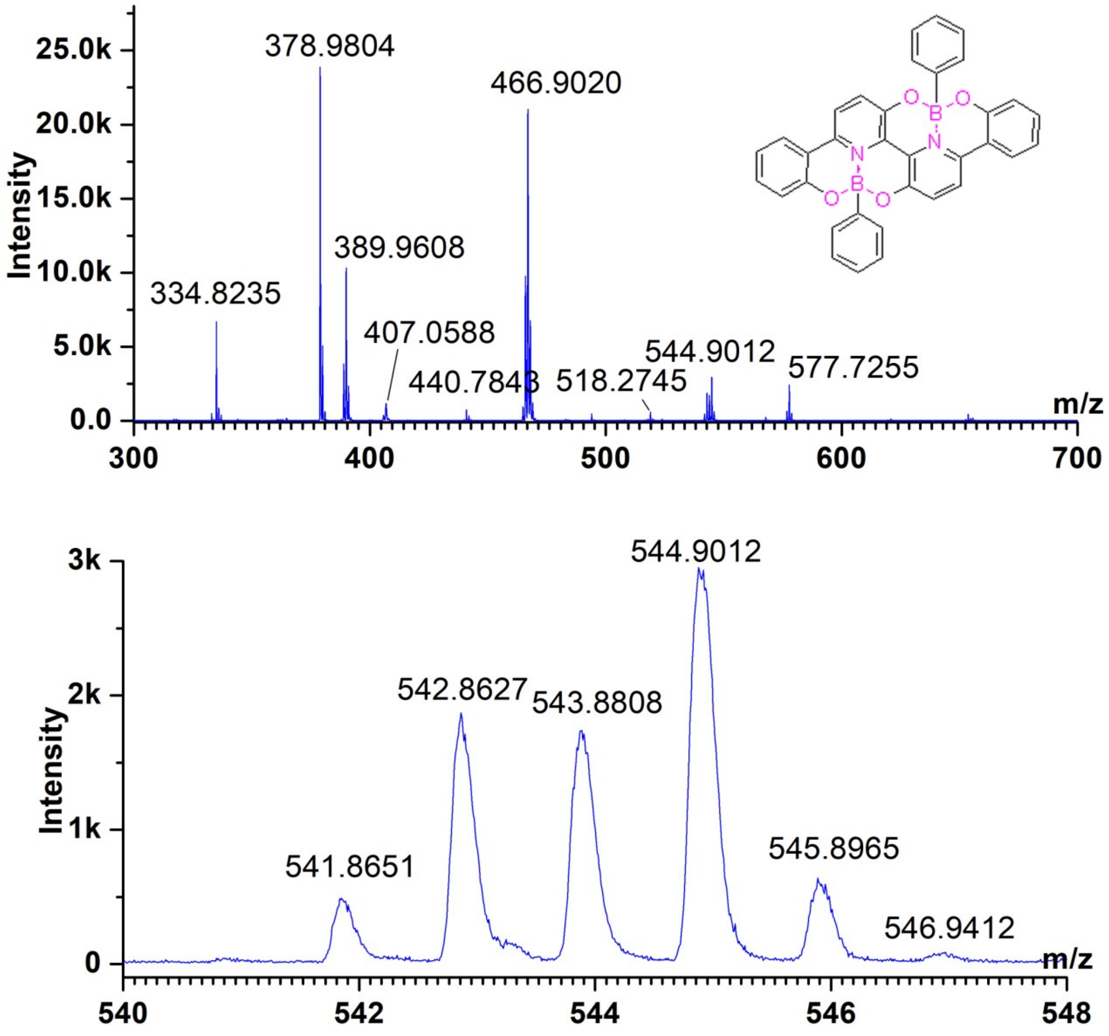

Figure S70. MALDI-TOF MS of OBN8-Ph

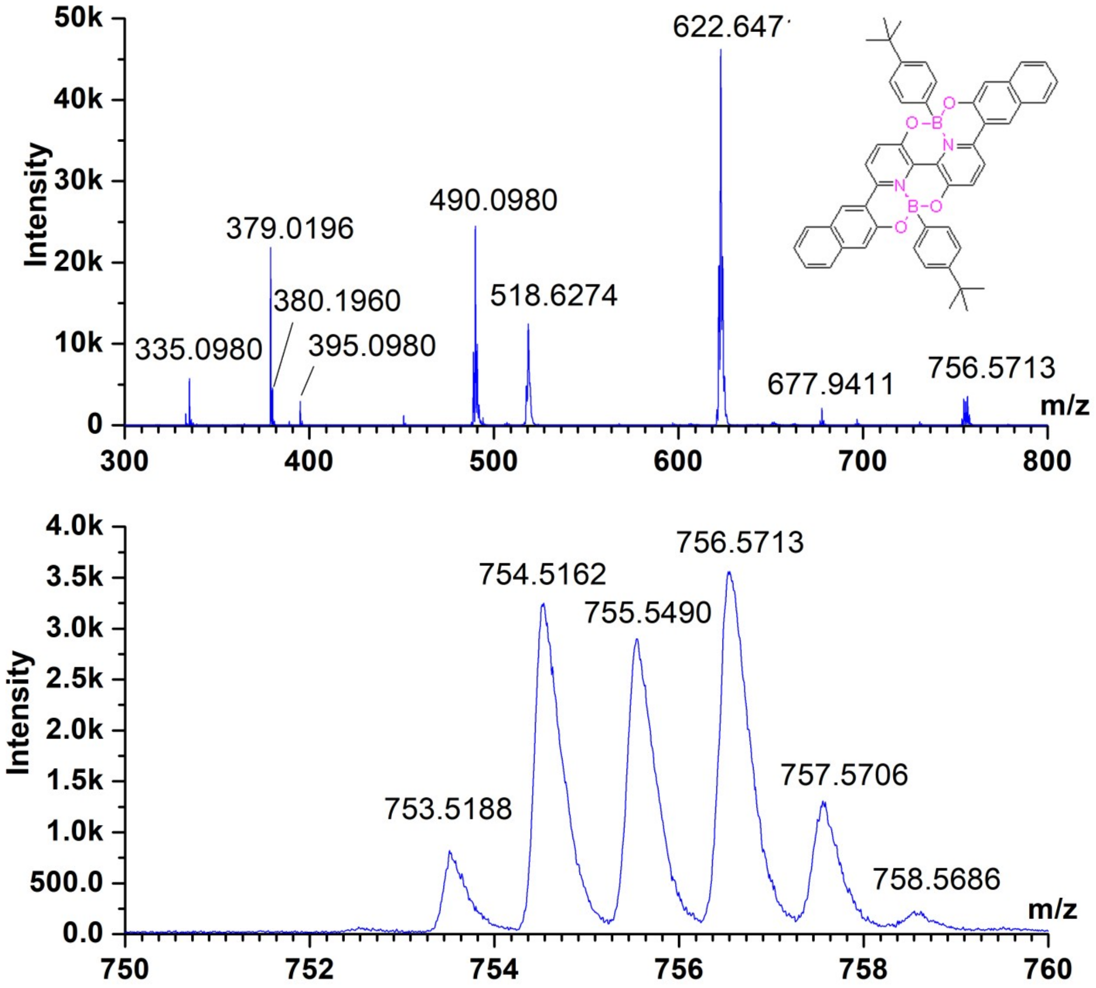

Figure S71. MALDI-TOF MS of OBN10-Ph

9. References

[1] M. J. Frisch, G. W. Trucks, H. B. Schlegel, G. E. Scuseria, M. A. Robb, J. R. Cheeseman, G. Scalmani, V. Barone, G. A. Petersson, H. Nakatsuji, X. Li, M. Caricato, A. V. Marenich, J. Bloino, B. G. Janesko, R. Gomperts, B. Mennucci, H. P. Hratchian, J. V. Ortiz, A. F. Izmaylov, J. L. Sonnenberg, Williams, F. Ding, F. Lipparini, F. Egidi, J. Goings, B. Peng, A. Petrone, T. Henderson, D. Ranasinghe, V. G. Zakrzewski, J. Gao, N. Rega, G. Zheng, W. Liang, M. Hada, M. Ehara, K. Toyota, R. Fukuda, J. Hasegawa, M. Ishida, T. Nakajima, Y. Honda, O. Kitao, H. Nakai, T. Vreven, K. Throssell, J. A. Montgomery Jr., J. E. Peralta, F. Ogliaro, M. J. Bearpark, J. J. Heyd, E. N. Brothers, K. N. Kudin, V. N. Staroverov, T. A. Keith, R. Kobayashi, J. Normand, K. Raghavachari, A. P. Rendell, J. C. Burant, S. S. Iyengar, J. Tomasi, M. Cossi, J. M. Millam, M. Klene, C. Adamo, R. Cammi, J. W. Ochterski, R. L. Martin, K. Morokuma, O. Farkas, J. B. Foresman, D. J. Fox, Gaussian 16 Rev. C.01, Wallingford, CT, 2016.

[2] a) R. Herges, D. Geuenich, *J. Phys. Chem.* A 2001, 105, 3214; b) D. Geuenich, K. Hess, F. Köhler, R. Herges, *Chem. Rev.* **2005**, *105*, 3758-3772.

[3] T. Lu, F. Chen, *J. Mol. Graph. Model.*, **2012**, *38*, 314-323.

[4] a) T. Lu, F. Chen, *J. Comput. Chem.* **2012**, *33*, 580-592; b) T. Lu, *J. Chem. Phys.*, **2024**, *161*, 082503.

[5] a) Z. Wang, "https://wongzit.github.io/program/pyaroma (accessed 18.01.2024)", py.Aroma 4; b) Y. Miyazawa, Z. Wang, M. Matsumoto, S. Hatano, I. Antol, E. Kayahara, S. Yamago, M. Abe, *J. Am. Chem. Soc.* **2021**, *143*, 7426. S122
